# Proteomic Analysis Reveals Commonly Secreted Proteins of Mesenchymal Stem Cells Derived from Bone Marrow, Adipose Tissue, and Synovial Membrane to Show Potential for Cartilage Regeneration in Knee Osteoarthritis

**DOI:** 10.1155/2021/6694299

**Published:** 2021-06-28

**Authors:** Yura Lee, Yo Seph Park, Na Young Choi, Yong Il Kim, Yong-Gon Koh

**Affiliations:** ^1^Department of Stem Cell Research, Research and Development Center, TJC Life, Seoul 08502, Republic of Korea; ^2^Department of Orthopaedic Surgery, Yonsei Sarang Hospital, Seoul 06698, Republic of Korea

## Abstract

Paracrine factors secreted by mesenchymal stem cells (MSCs) reportedly modulate inflammation and reparative processes in damaged tissues and have been explored for knee osteoarthritis (OA) therapy. Although various studies have reported the effects of paracrine factors in knee OA, it is not yet clear which paracrine factors directly affect the regeneration of damaged cartilage and which are secreted under various knee OA conditions. In this study, we cultured MSCs derived from three types of tissues and treated each type with IL-1*β* and TNF-*α* or not to obtain conditioned medium. Each conditioned medium was used to analyse the paracrine factors related to cartilage regeneration using liquid chromatography-tandem mass spectrometry. Bone marrow-, adipose tissue-, and synovial membrane-MSCs (all-MSCs) exhibited expression of 93 proteins under normal conditions and 105 proteins under inflammatory conditions. It was confirmed that the types of secreted proteins differed depending on the environmental conditions, and the proteins were validated using ELISA. The results of Gene Ontology and Kyoto Encyclopedia of Genes and Genomes pathway analysis using a list of proteins secreted by all-MSCs under each condition confirmed that the secreted proteins were closely related to cartilage repair under inflammatory conditions. Protein-protein interaction networks were confirmed to change depending on environmental differences and were found to enhance the secretion of paracrine factors related to cartilage regeneration under inflammatory conditions. In conclusion, our results demonstrated that compared with knee OA conditions, the differential expression proteins may contribute to the regeneration of damaged cartilage. In addition, the detailed information on commonly secreted proteins by all-MSCs provides a comprehensive basis for understanding the potential of paracrine factors to influence tissue repair and regeneration in knee OA.

## 1. Introduction

Mesenchymal stem cells (MSCs) can be easily obtained from different cell sources such as bone marrow (BM), adipose tissue (AT), and synovial membrane (SM). They have self-renewal and trilineage differentiation potential [1–4]. MSCs are also known to secrete various paracrine factors including cytokines, chemokines, growth factors, and extracellular vesicles. Paracrine signalling is a form of cell-to-cell communication in which a cell produces a signal to induce changes in nearby cells, altering the behaviour or differentiation of those cells. Paracrine factors secreted by MSCs induce surrounding cells to differentiate into mature cell lines and regulate tissue inflammation or recovery processes [5]. Thus, there is increasing evidence that MSCs play a role in regenerating damaged tissue through the paracrine effect on surrounding cells [2, 6, 7]. Owing to these characteristics, MSCs are widely studied for the treatment for various diseases. According to the official database of ClinicalTrials.gov, 630 MSC-based clinical trials have been reported, and 10% or more of them are actively being conducted to assess the potential of MSCs for knee osteoarthritis (OA) therapy.

Knee OA is a degenerative disease caused by various factors, including abnormal mechanical stress, ageing, obesity, and genetic factors, and results in inflammation in joints and degradation of cartilage tissue [8]. The cartilage of the knee joint where knee OA occurs is difficult to regenerate after injury due to the nature of cartilage; therefore, therapeutic treatments are limited to conservative care or artificial joint surgery [9, 10]. To overcome these therapeutic limitations and augment cartilage regeneration, various tissue-derived MSCs are being used for the development of cell therapy for knee OA treatment [11–14]. According to the results of recent studies, injecting MSC-conditioned medium (CM) into the anterior cruciate ligament transection rat model is sufficient to mediate cartilage regeneration function [11, 15–17]; thus, interest in efficacious knee OA treatments using MSC-derived paracrine factors has increased [14, 18–21]. However, although several studies have actively investigated paracrine factors secreted by each type of MSC obtained from various tissues under general culture conditions [22–24], it is still unclear which paracrine factors affect the regeneration of damaged cartilage.

To elucidate the therapeutic role of paracrine factors secreted by MSCs, it is necessary to identify the factors that are commonly secreted by MSCs under various environmental conditions, since each type of tissue-derived MSC secretes different paracrine factors [24–26]. However, little is known about how environmental conditions similar to knee OA affect paracrine factors [27, 28]. Therefore, in the present study, we aimed to investigate the major paracrine factors related to cartilage regeneration by identifying the differences in the types and characteristics of commonly secreted proteins by BM-, AT-, and SM-MSCs under various conditions.

## 2. Materials and Methods

### 2.1. Isolation and Culture of Human BM-, AT-, and SM-MSCs

The study was approved by the Institutional Review Board of Yonsei Sarang Hospital (IRB number: YSSR 2020-09-001), and informed consent was obtained from all donors.

Human BM-MSCs (*n* = 3) were purchased from the American Type Culture Collection (Manassas, VA, USA). The frozen cells were thawed and plated at a density of 5,000 cells/cm^2^ in a 75 cm^2^ culture flask with a complete medium containing alpha modification of Eagle's medium (*α*-MEM; Welgene, Daegu, Republic of Korea), 10% foetal bovine serum (FBS; HyClone, Logan, UT, USA), and 1% penicillin-streptomycin (P/S; HyClone).

Human AT (*n* = 3) were harvested by liposuction surgery. The tissue was digested with 0.3% collagenase type 1 (Worthington-Biochemical, Lakewood, NJ, USA) at 37°C for 90 min with gentle shaking. After digestion, the digested tissues were washed with phosphate-buffered saline (PBS; HyClone) and the undigested tissues were removed using a 70 *μ*m strainer (BD Biosciences, San Diego, CA, USA). The cells obtained by centrifugation at 645 × *g* for 5 min were plated at a density of 5,000 cells/cm^2^ in a 75 cm^2^ culture flask with complete medium [29].

Human SM (*n* = 3) were harvested from the knees of patients with OA during total knee arthroplasty. The tissue was placed on a petri dish and finely minced using sterile scissors. The minced tissue was digested with 0.25% Trypsin-EDTA (HyClone) at 37°C for 30 min. After digestion, 0.3% of collagenase type 1 was added and gently shaken at 37°C for 90 min. Undigested tissues were removed using a 40 *μ*m strainer (BD Biosciences), and cells were obtained by centrifugation at 645 × *g* for 5 min. The obtained cells were plated at a density of 5,000 cells/cm^2^ in a 75 cm^2^ culture flask with complete medium [30, 31].

All cells used in the experiment were cultured in a humidified incubator at 5% CO_2_ at 37°C. The medium was changed every 2–3 days, and cells were subcultured at 80–90% confluence following treatment with 0.25% Trypsin-EDTA at 37°C for 5 min. The cells were washed and harvested by centrifugation at 645 × *g* for 5 min and then plated at a density of 5,000 cells/cm^2^ [32, 33]. Passage 4 cells were used for all cell types.

### 2.2. Flow Cytometry

For flow cytometry analysis, 1 × 10^5^ cells were suspended in 100 *μ*L of PBS [33]. The following monoclonal antibodies were used to stain the cells: fluorescein isothiocyanate (FITC) mouse anti-human CD14 (clone M5E2), FITC mouse anti-human CD34 (clone 581), FITC mouse anti-human CD45 (clone HI30), phycoerythrin (PE) mouse anti-human CD79a (clone HM47), and FITC mouse anti-human leukocyte antigen-DR (HLA-DR; clone G46-6) were used as haematopoietic or endothelial cell markers. PerCP-Cy5.5 mouse anti-human CD73 (clone AD2), PerCP-Cy5.5 mouse anti-human CD90 (clone 5E10), and PerCP-Cy5.5 mouse anti-human CD105 (clone 266) were used as MSC-specific markers. As isotype controls, FITC mouse IgG1 *κ* (clone MOPC-21), FITC mouse IgG2a *κ* (clone G155-178), PE mouse IgG1 *κ* (clone MOPC-21), and PerCP-Cy5.5 mouse IgG1 *κ* (clone MOPC-21; all from BD Biosciences) were used [34]. Stained cells were acquired using FACSCalibur (BD Biosciences), and data analysis was performed using CellQuest software (BD Biosciences).

### 2.3. *In Vitro* Differentiation Assay

For adipogenic differentiation, cells were seeded into 12-well plates at a density of 3 × 10^4^ per well. After 2 days, differentiation was induced using adipogenic differentiation medium (containing *α*-MEM, 10% FBS, 1% P/S, 0.5 mM of 3-isobutyl-1-methylxanthine (Sigma-Aldrich, St. Louis, MO, USA), 1 *μ*M of dexamethasone (Sigma-Aldrich), 200 *μ*M of indomethacin (Sigma-Aldrich), and 10 *μ*g/mL of insulin (Gibco, Waltham, Massachusetts, USA)) [35]. The medium was changed every 2–3 days for 14 days, and differentiated cells were stained with Oil Red O (Sigma-Aldrich) according to the manufacturer's instructions.

For osteogenic differentiation, cells were seeded into 12-well plates at a density of 3 × 10^4^ per well. After 2 days, differentiation was induced using osteogenic differentiation medium (containing *α*-MEM, 10% FBS, 1% P/S, 0.1 *μ*M dexamethasone, 50 *μ*M of L-ascorbic acid 2-phosphate sesquimagnesium salt hydrate (Sigma-Aldrich), and 20 mM of *β*-glycerophosphate disodium salt hydrate (Sigma-Aldrich)) [36]. The medium was changed every 2–3 days for 14 days, and differentiated cells were stained with Alizarin Red S (IHC World, Woodstock, MD, USA) according to the manufacturer's instructions.

For chondrogenic differentiation, cells were seeded into 12-well plates at a density of 1.2 × 10^4^ per well. After 2 days, differentiation was induced using chondrogenic differentiation medium (containing Dulbecco's minimal EM-high glucose (HyClone), 10% FBS, 1% P/S, 0.1 *μ*M of dexamethasone, 1X insulin-transferrin-selenium (Gibco), 50 *μ*M of L-ascorbic acid 2-phosphate sesquimagnesium salt hydrate, and 5 ng/mL of transforming growth factor *β*1 (TGF-*β*1; PeproTech, Rocky Hill, NJ, USA)) [37]. The medium was changed every 2–3 days for 21 days, and differentiated cells were stained with Alcian Blue 8GX (Sigma-Aldrich) according to the manufacturer's instructions.

### 2.4. Preparation of CM

The BM-, AT-, and SM-MSCs were maintained under two conditions based on environmental differences: (i) normal condition: without inflammatory cytokines (tumour necrosis factor-*α* (TNF-*α*; PeproTech) and interleukin-1*β* (IL-1*β*; PeproTech)), and (ii) inflammatory condition: treated with 10 ng/mL of TNF-*α* and 10 ng/mL of IL-1*β* [38–41]. To prepare CM, cells were cultured in a 75 cm^2^ culture flask in *α*-MEM containing 10% FBS. When MSCs were approximately 80–90% confluent at passage 4, the cells were switched to serum-free *α*-MEM for overnight incubation and treated with conditions (i) or (ii) for 6 h. Cultures were refed with 15 mL of serum-free *α*-MEM and incubated for 48 h. Here, we used serum-free medium to avoid interference from albumin-enriched FBS. Then, the CM was collected and filtrated through a 0.2 *μ*m filter to remove cellular debris. Thereafter, the CM was concentrated 25-fold using ultrafiltration units with 3 kDa cut-off filters (Amicon Ultra; Merck Millipore, Watford, UK) at 4000 × *g* for 1 h [42]. The protein concentration of the CM was measured using the Pierce BCA Protein Assay Kit (Thermo Fisher Scientific, Waltham, MA, USA) according to the manufacturer's instructions.

### 2.5. In-Solution Digestion

Briefly, 100–200 *μ*g of protein was denatured using 8 M urea in 50 mM heavy carbonate ammonium buffer (pH 7.8) and allowed to react at room temperature for 3 h, followed by reduction using 10 mM dithiothreitol for 2 h at room temperature. The proteins were alkylated with 10 mM iodoacetamide in the dark at room temperature for 1 h and then diluted more than 10-fold using 50 mM ammonium bicarbonate solution. In-solution digestion was carried out by adding trypsin to the protein solution with an enzyme-to-protein ratio of 1 : 50 (*w*/*w*) at 37°C for 18 h. Finally, formic acid was added to each sample to stop the reaction. Samples were stored at -80°C until further analysis [43].

### 2.6. Liquid Chromatography with Tandem Mass Spectrometry

Liquid chromatography with tandem mass spectrometry (LC-MS/MS) analysis was conducted by the National Instrumentation Center for Environmental Management (NICEM; Seoul National University, Seoul, Republic of Korea). The fractionated peptide samples were analysed using the LC-MS/MS system, which was a combination of an Easy-nLC 1000 (Thermo Fisher Scientific) and EASY-Spray Ion Source (Thermo Fisher Scientific) on a Q Exactive Orbitrap Mass Spectrometer (Thermo Fisher Scientific). Peptides were separated on a two-column setup with a trap column (Thermo Fisher Scientific Acclaim PepMap 100 C18 HPLC Columns; 100 *μ*m × 2 cm, nanoViper C18, particle size of 5 *μ*m; Thermo Fisher Scientific, part number 164564) and an analytical column (Thermo Fisher Scientific; 75 *μ*m i.d.×50 cm long, 2 *μ*m C18 beads, spherical, fully porous, ultrapure). The peptide samples were separated using a 180 min linear gradient from 10% to 40% solvent B (100% acetonitrile and 0.1% formic acid) in all samples. The spray voltage was 2.2 kV in a positive ion mode, and the temperature of the heated capillary was set to 300°C. Mass spectra were acquired in a data-dependent manner using the top ten methods on the Q Exactive Mass Spectrometer. Xcalibur software version 3.1 was used to collect MS data. The Orbitrap analyser scanned precursor ions with a mass range of 350–1,800 *m*/*z* with a resolution of 70,000 at *m*/*z* 200. The automatic gain control (AGC) target value was 3 × 10^6^, and the isolation window for MS/MS was 2 *m*/*z*. Higher-energy C-trap dissociation scans were acquired at a resolution of 17,500 and normalised collision energy of 27. The AGC target value for MS/MS was 1 × 10^5^. The maximum ion injection time for the survey scan and MS/MS scan was 100 ms. Dynamic exclusion was enabled with an exclusion period of 15 s [44]. Mass data were acquired automatically using MaxQuant version 1.6 and Proteome Discoverer 2.3 (Thermo Fisher Scientific). LC-MS/MS analysis was performed thrice on the samples (triplicates for each MSC type under normal or inflammatory conditions).

### 2.7. Bioinformatics Analysis

The original MS/MS file data were acquired automatically with Proteome Discoverer 2.3 (version 2.3.0.523) for data analysis. Peptides were identified using SEQUEST-HT against the UniProtKB database (uniprot-homosapiens-201810) integrated into Proteome Discoverer. The processing workflow consisted of the following nodes: two maximum missed cleavages, peptide length range of 6–144 amino acids, precursor mass tolerance of 10 ppm, fragment mass tolerance of 0.02 Da, cysteine carbamidomethylation as a static modification, and oxidation as a dynamic modification. Peptide validation settings were identified using a target false discovery rate (FDR; strict) for peptide-spectrum match (PSM) of 0.01, target FDR (relaxed) for PSM of 0.05, and peptide filter confidence of at least high level.

The identified proteins were associated with Gene Ontology (GO) terms to determine their biological and functional properties. The three main types of annotations, namely, cellular components (CC), molecular functions (MF), and biological processes (BP), were obtained from the GO website at http://www.geneontology.org.

Protein pathways were generated using Kyoto Encyclopedia of Genes and Genomes (KEGG) analysis. In addition, the identified proteins were entered into the STRING database (https://string-db.org/) to predict and visualise the protein-protein interactions (PPI) under various environmental conditions. A representative network was obtained with high confidence in data settings with a minimum interaction score of 0.7.

### 2.8. Enzyme-Linked Immunosorbent Assay

Cell culture supernatants were collected as described in the preparation of CM. The concentration of proteins in CM was assessed using a commercially available enzyme-linked immunosorbent assay (ELISA) kit (RayBiotech, GA, USA) according to the manufacturer's instructions [45]. Assay kits for the following proteins were used: human thrombospondin 2 (TSP-2) and human TNF-inducible gene 6 (TSG-6). All measurements were performed in duplicate.

## 3. Results

### 3.1. Characterization of BM-, AT-, and SM-MSCs

**Figure 1:**
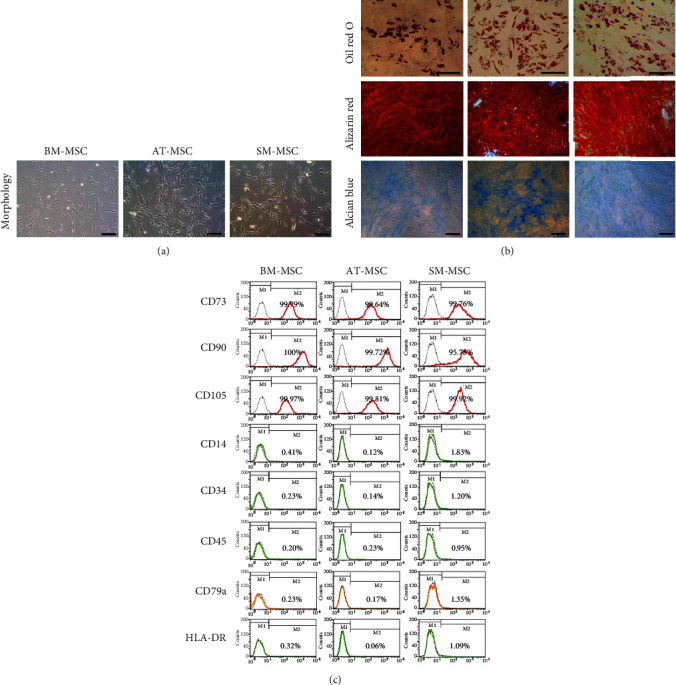
Representative characterization of bone marrow (BM), adipose tissue (AT), and synovial membrane (SM) mesenchymal stem cells (MSCs). (a) Phase-contrast microscopy images of MSCs (scale bar = 100 *μ*m). (b) Differentiation potential of MSCs. Adipogenic differentiation (Oil Red O, top), osteogenic differentiation (Alizarin Red S, middle), and chondrogenic differentiation (Alcian Blue, bottom). (c) The immunophenotyping of MSCs using flow cytometry. Positive (CD73, CD90, and CD105) and negative (CD14, CD34, CD45, CD79a, and human leukocyte antigen-DR (HLA-DR)) markers. The histogram is shown with an overlay isotype control.

The human BM-, AT-, and SM-MSCs derived from each tissue were found to grow in a spindle-shaped manner (Figure 1(a)). We performed *in vitro* differentiation assays to confirm the trilineage differentiation potential of each MSC type. As a result, each MSC type differentiated into adipocytes, osteoblasts, and chondrocytes when cultured in the appropriate differentiation medium (Figure 1(b)). Flow cytometry analysis was performed to confirm that MSCs expressed cell surface antigens. In each MSC type, the positive markers CD73, CD90, and CD105 were expressed at 95% or higher, and the negative markers CD14, CD34, CD45, CD79a, and HLA-DR were expressed at less than 2% (Figure 1(c)). These results demonstrated that the human tissue-derived cells used in the present study had typical MSC characteristics (Supplementary Figure 1).

### 3.2. Commonly Secreted Proteins among the Three Types of MSCs

**Figure 2:**
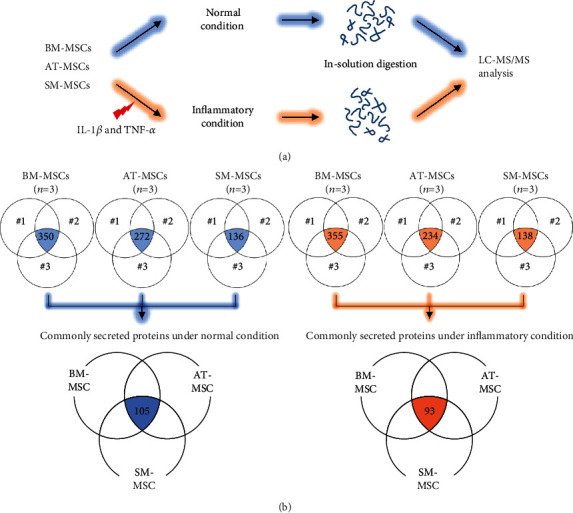
Mass spectrometry analysis of the conditioned medium for BM-, AT-, and SM-MSCs. (a) Workflow of the preparation of conditioned medium for LC-MS/MS. (b) Venn diagram of commonly secreted proteins by three donors for each MSC type. A total of 105 and 93 proteins were commonly secreted by BM-, AT-, and SM-MSCs (all-MSCs) under normal and inflammatory conditions, respectively.

LC-MS/MS (*n* = 3 per tissue source) was performed to identify the proteins secreted by each MSC type under normal or inflammatory conditions (Figure 2(a)). We confirmed that the number of proteins secreted by each MSC type under normal or inflammatory conditions was 350 and 355 in BM-MSCs, 272 and 234 in AT-MSCs, and 136 and 138 in SM-MSCs, respectively (detailed information in Supplementary Table 1). Comparison of common proteins identified 105 proteins under normal conditions and 90 proteins under inflammatory conditions to be commonly secreted by BM-, AT-, and SM-MSCs (all-MSCs; Figure 2(b) and Table 1).

### 3.3. GO Analysis

**Figure 3:**
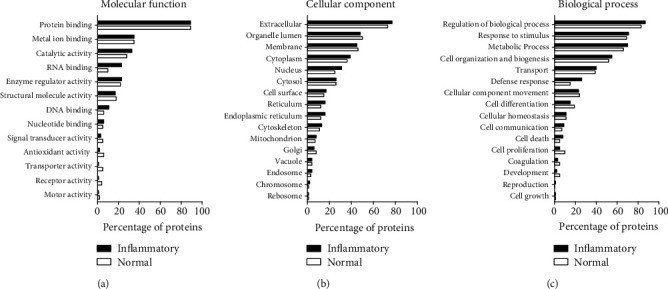
Gene Ontology (GO) annotation analysis of the commonly secreted proteins by all mesenchymal stem cells (MSCs) under normal and inflammatory conditions: (a) molecular function; (b) cellular component; (c) biological process.

GO analysis was performed to confirm the functional differences between commonly secreted proteins by all-MSCs under normal and inflammatory conditions. As a result, in MF, the percentage of “protein binding” of the secreted proteins under both conditions was confirmed to be 88% or more, while the percentages of other categories were 35% or less (Figure 3(a)). In CC, the percentage of “extracellular” of the secreted proteins under both conditions was confirmed to be 73% or more, and that of the other categories was 50% or less (Figure 3(b)). In BP, the percentage of secreted proteins under both conditions including “regulation of biological process,” “response to stimulus,” “metabolic process,” and “cell organization and biogenesis” was confirmed to be approximately 52% or more, with the percentages of these categories being higher under inflammatory conditions than under normal conditions. The percentages of other categories were confirmed to be 40% or less (Figure 3(c)).

### 3.4. KEGG Pathway Analysis

**Figure 4:**
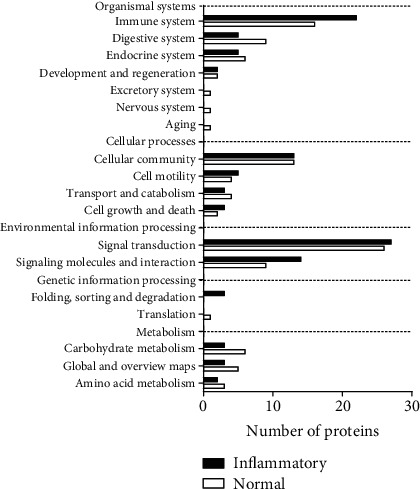
Kyoto Encyclopedia of Genes and Genomes **(**KEGG) pathway analysis of the commonly secreted proteins by all mesenchymal stem cells (MSCs) under normal and inflammatory conditions.

KEGG pathway analysis was conducted to identify signalling pathways related to proteins commonly secreted by all-MSCs under normal and inflammatory conditions. We confirmed that the pathway most closely related to proteins secreted under both conditions was “signal transduction.” The main pathways were similar under both conditions, but the levels of the secreted proteins were higher under inflammatory conditions than under normal conditions (Figure 4).

### 3.5. Changes in PPI Networks Depending on Environmental Differences

**Figure 5:**
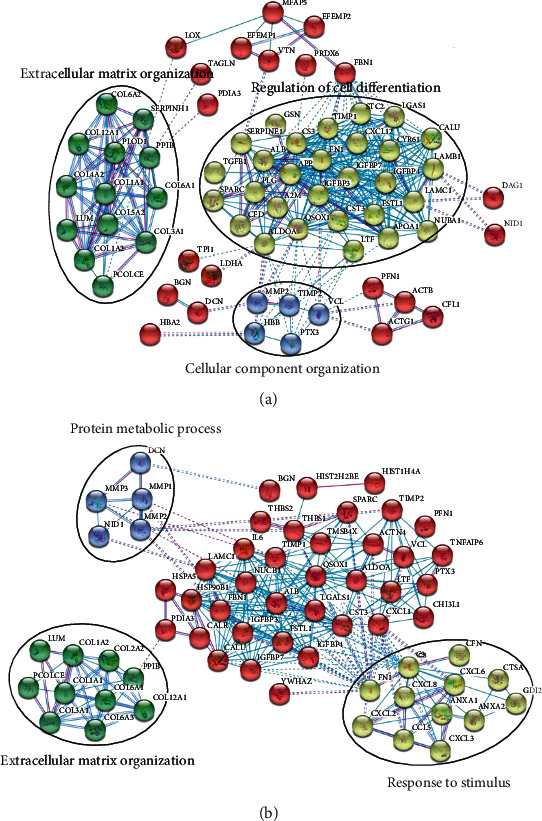
Protein-protein interaction (PPI) network analysis of commonly secreted proteins by all mesenchymal stem cells (MSCs) under normal (a) and inflammatory (b) conditions. The coloured lines represent the two types of evidence for active interaction sources. Light blue line, database evidence; purple line, experimental evidence.

PPI network analysis was performed using the STRING database to confirm how the interaction networks of commonly secreted proteins by all-MSCs were altered because of environmental differences. Thus, excluding unconnected nodes, the PPI network under normal condition was composed of 102 nodes and 368 edges. The PPI network under inflammatory condition was composed of 92 nodes and 309 edges (interaction score > 0.7), confirming three main clusters under each condition. Under normal conditions, “extracellular matrix (ECM) organization” constituted 13 proteins, primarily including the collagen family, and “regulation of cell differentiation” constituted 29 proteins, including proteins such as insulin-like growth factor-binding protein 3/4/7, gelsolin, and stanniocalcin-2. In addition, “cellular component organization” constituted 5 proteins, including vinculin (VCL) (Figure 5(a)). Under the inflammatory condition, “ECM organization” constituted 10 proteins, including those from the collagen family, and “protein metabolic process” constituted 5 proteins, including matrix metalloproteinase (MMP). Moreover, “response to stimulus” constituted 12 proteins including C-X-C motif chemokine 2/3/6/8 and annexin A1 (Figure 5(b)).

### 3.6. Identification of Proteins with Tissue Regeneration Potential

We identified proteins related to tissue regeneration among the commonly secreted proteins by all-MSCs under normal and inflammatory conditions. The categories affecting tissue regeneration were classified as anti-inflammation, anti-apoptosis, ECM-cell interactions, homeostasis, inhibition of MMPs, and regeneration of chondrocytes. Proteins related to tissue regeneration were found to be secreted under all or specific environments. Except for the category of ECM-cell interactions, the number of secreted proteins was larger under inflammatory conditions than under normal conditions. We identified that proteins such as TSG-6 and thrombospondin 1 (TSP-1) are secreted only under inflammatory conditions (Table 2).

### 3.7. Validation of Commonly Secreted Proteins by Three Types of MSCs under the Two Environmental Conditions

**Figure 6:**
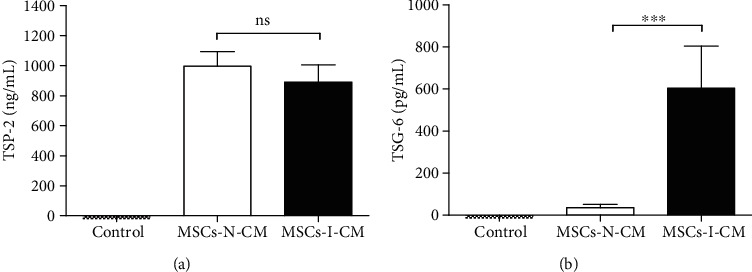
Validation experiments of key protein expression under various knee OA conditions using ELISA: (a) human TSP-2; (b) human TSG-6. ^∗∗∗^*P* < 0.0001. ns: not significant (*n* = 9, three donors for each MSC type); N-CM: normal condition-conditioned medium; I-CM: inflammatory condition-conditioned medium.

ELISA was performed to validate the proteins presented in Table 2. We confirmed that TSP-2 was secreted under both environmental conditions, whereas TSG-6 was secreted under inflammatory conditions. The expression level of TSP-2, confirmed to be secreted under the two conditions, was higher under the normal condition than under the inflammatory condition. However, the concentration of TSP-2 in the CM was not significantly different between the normal and inflammatory conditions (Figure 6(a)). The expression level of TSG-6 was surprisingly 15 times higher under the inflammatory condition than under the normal condition (Figure 6(b)). The control was serum-free medium, and no protein was detected in it.

## 4. Discussion

We investigated proteins predicted to exhibit cartilage regeneration potential by confirming the differences and characteristics of proteins commonly secreted by human BM-, AT-, and SM-MSCs, depending on environmental differences. Thus far, cartilage repair studies using MSCs derived from various tissues, including BM, AT, and SM, have reported these cells to exhibit similar cartilage regenerative properties despite differences in paracrine factors secreted by each MSC type [46–48]. Based on these results, we hypothesised that there are paracrine factors commonly secreted by MSCs derived from tissues of different origins. Therefore, MSCs were isolated from each tissue; the cultured cells were confirmed to possess MSC characteristics defined by the International Society for Cellular Therapy. The paracrine factors secreted by each MSC type were then identified using LC-MS/MS analysis. The identified proteins were validated by ELISA, finding differences in the secreted proteins depending on the tissue origin of MSCs and the presence or absence of inflammatory factors (Supplementary Table 1).

GO analysis was performed to confirm the properties of commonly secreted proteins by all-MSCs under normal or inflammatory conditions. We found that the largest proportion of MF was “protein binding,” suggesting that direct regulation of PPIs may be a major regulatory process carried out by proteins secreted by MSCs [49]. BP included “regulation of biological process,” “response to stimulus,” “metabolic process,” and “cell organization and biogenesis,” indicating that processes functionally relevant to commonly secreted proteins by all-MSCs play an important role in tissue repair [23, 49].

In addition, KEGG pathway analysis was conducted to confirm the biological function of paracrine factors commonly secreted by all-MSCs, and several pathways related to regeneration were verified. Environmental information processing included several pathways such as signal transduction, and the subcategories of signal transduction were identified in 11 pathways (data not shown). Among these pathways, the TGF-*β* signalling pathway is known to be essential for tissue regeneration by activating Smad signalling to regulate collagen and aggrecan expression [50, 51]. In addition, this pathway has been reported to maintain homeostasis and inhibit the degradation of articular chondrocytes by modulating proinflammatory cytokines, eventually repairing or regenerating damaged cartilage tissue [52]. Taken together, each MSC type secreted proteins related to the TGF-*β* signalling pathway, and these proteins may contribute to the maintenance and regeneration of damaged cartilage.

We confirmed that the processes related to repair and regeneration were more under inflammatory conditions by GO and KEGG pathway analyses. The mechanism underlying this phenomenon was studied using PPIs. We found that ECM organization was a process observed under both environmental conditions. ECM stores and appropriately supplies factors necessary for cell growth and differentiation and supports spontaneous regeneration mechanisms to recover damaged tissues, displaying potential as a therapeutic avenue [53]. Meanwhile, as expected, a “cluster of responses to stimuli” was formed under inflammatory conditions. According to several studies reporting the association of paracrine factors secreted by MSCs under inflammatory conditions, the secretion and therapeutic effects of MSCs may be enhanced by inflammatory stimuli and crosstalk [54–56]. In addition, secretion of paracrine factors is reportedly increased in MSCs stimulated with inflammatory factors [41, 57]. These findings indicate that paracrine factors contribute to the regeneration of damaged tissue under any condition. Furthermore, it was confirmed that paracrine factors secreted under inflammatory conditions are closely related to the repair and regeneration of cartilage, and several studies have reported them to regulate endogenous cellular responses and mediate cartilage regeneration in damaged tissue [6, 58]. Thus, we concluded that paracrine factors released under inflammatory conditions facilitate the recovery and regeneration of damaged cartilage.

Table 2 [6, 59–91] shows the proteins predicted to be involved in the regeneration of damaged tissue. It was confirmed that ECM-cell interactions included collagen *α*-1(I) chain, decorin, biglycan, lumican, VCL, fibronectin 1, TSP-1, and TSP-2. These proteins are primarily known to constitute cartilage ECM and provide structural support to cells and tissues. Furthermore, they modulate cellular signals that can affect tissue organization, cellular proliferation, matrix adhesion, growth factors, and cytokine responses. They also reportedly protect the surface of collagen type I and II fibres from degradation [63, 92–94]. The articular cartilage, a specialised form of hyaline cartilage, is avascular and has a poor capacity for self-repair [95]. Therefore, ECM-cell interactions are considered essential for cartilage regeneration, and the proteins identified herein are similar to those reported in other studies [94, 96].

Knee OA development has been observed to increase proinflammatory cytokines such as IL-1*β* and TNF-*α* in the joint cavity, which can trigger chondrocyte apoptosis [97, 98]. In addition, lactoferrin (LTF) was reported to inhibit IL-1*β*-mediated chondrocyte apoptosis by regulating the activity of cyclic AMP-responsive element-binding protein through protein kinase B signalling. LTF also promoted cartilage regeneration by increasing the expression of collagen type II in a rat OA model. Thus, LTF secreted by human MSCs can be anticipated to have anti-apoptotic and regenerative effects on chondrocytes in damaged cartilage. In a study of TSP-2, Jeong et al. [82] showed that intra-articular injection of human umbilical cord blood-MSCs into an osteochondral defect rat model exerts a regenerative effect on damaged cartilage through paracrine factors, of which TSP-2 was the major paracrine player. These findings demonstrate the therapeutic potential of TSP-2-mediated paracrine action of human MSCs, which can regenerate cartilage in knee OA treatment.

Furthermore, inflammation is accompanied by the breakdown of the ECM, the main component of articular cartilage [99]. The synovial fluid of knee OA patients exhibits a high level of proinflammatory cytokines as well as different types of MMPs [100]. Synovitis is mostly associated with gradual progressive damage to cartilage and severe pain. The pain in knee OA may be caused by the activation of nociceptive pathways by nerve growth factor, which in turn may occur due to inflammation. Therefore, inflammation is a major therapeutic target to relieve OA pain, and alleviation of inflammation and suppression of MMPs may be key strategies for knee OA treatment [101]. Interestingly, in our study, we found that TSG-6 and TSP-1 proteins were secreted only under inflammatory conditions. These proteins attenuate proinflammatory cytokines and MMPs by modulating the nuclear factor *κ*B pathway and enhance the production of collagen type II [65, 102–104]. TSG-6 and TSP-1 have anti-inflammatory and tissue-protective properties and can enable the regeneration of damaged cartilage.

We confirmed the level of expression depending on the environment for some of the abovementioned proteins. TSP-2 was identified in both conditions, and it was confirmed that its expression level was similar between the two conditions. This suggests that TSP-2 is a paracrine factor that is secreted by MSCs under various conditions and plays an essential role in cartilage regeneration. TSG-6, which was only secreted under the inflammatory condition, showed a dramatically higher expression level under the inflammatory condition than under the normal condition, supporting the result that TSG-6 is induced by stimulation by IL-1 and TNF-*α* [105].

The limitation of this study is that the identified proteins could not be confirmed to regenerate damaged cartilage through actual studies. However, it was meaningful to check the protein list for paracrine factors secreted by MSCs under normal culture conditions compared to those secreted under inflammatory conditions. Moreover, according to recent studies, the paracrine factors identified in this study have an effect on cartilage regeneration [62–66]. Thus, commonly secreted paracrine factors by each MSC type provide valuable information for understanding the potential of damaged knee OA regeneration.

## 5. Conclusions

We identified proteins commonly secreted by human BM-, AT-, and SM-MSCs under normal and inflammatory conditions and consequently found paracrine factors that are predicted to be closely related to cartilage regeneration under inflammatory conditions. Therefore, our study sheds light on paracrine factors as potential therapeutic options for knee OA.

## Figures and Tables

**(a):** 

Identified proteins of normal condition (105 proteins)		
Accession	Description	Gene symbol
A0A024QYT5	Serpin peptidase inhibitor, clade E	SERPINE1
A0A024R1U8	Insulin-like growth factor-binding protein 4	IGFBP4
A0A024R2W4	Dystroglycan 1	DAG1
A0A024R462	Fibronectin 1	FN1
A0A024R6R4	Matrix metallopeptidase 2	MMP2
A0A024R8V7	TIMP metallopeptidase inhibitor 2	TIMP2
A0A024RDW8	Collagen, type IV, alpha 2	COL4A2
A0A087WTA8	Collagen alpha-2(I) chain	COL1A2
A0A087X0S5	Collagen alpha-1(VI) chain	COL6A1
A0A0A0MT01	Gelsolin	GSN
A0A0F7G8J1	Plasminogen	PLG
A0A140VJI7	Testicular tissue protein Li 61	ECM1
A0A161I202	Lactoferrin	LTF
A0A172Q381	Endosialin	CD248
A1L4H1	Soluble scavenger receptor cysteine-rich domain-containing protein SSC5D	SSC5D
A4D2D2	Procollagen C-endopeptidase enhancer	PCOLCE
A6XND1	Insulin-like growth factor-binding protein 3	IGFBP3
A8K2H4	Cathepsin B	CTSB
A8K7Q1	Nucleobindin 1	NUCB1
A8KAJ3	EGF-containing fibulin-like extracellular matrix protein 1	EFEMP1
B2R582	C-type lectin domain family 3, member B	CLEC3B
B2R5M9	Procollagen-lysine, 2-oxoglutarate 5-dioxygenase	PLOD1
B2RBS8	Albumin	ALB
B2RCM5	EG-containing fibulin-like extracellular matrix protein 2	EFEMP2
B2RDW0	Calmodulin 2 (phosphorylase kinase, delta)	CALM2
B4DDQ2	Biglycan	BGN
B4DNG0	Olfactomedin-like protein 3	OLFML3
B4DPH4	Plasminogen	PLG
B4DPQ0	Complement C1r subcomponent	C1R
B4DPZ5	Polymerase I and transcript release factor	PTRF
B4DU16	Fibronectin 1	FN1
B4E3Q1	Calsyntenin-1	CLSTN1
D0PNI2	Lysyl oxidase	LOX
D1MGQ2	Alpha-2 globin chain	HBA2
D3DTX7	Collagen, type I, alpha 1	COL1A1
D3YTG3	Target of Nesh-SH3	ABI3BP
D6RF35	Vitamin D-binding protein	GC
D9ZGG2	Vitronectin	VTN
F8VR42	Dynein regulatory complex subunit 2	CCDC65
F8W6I7	Heterogeneous nuclear ribonucleoprotein A1	HNRNPA1
H0YGS3	Microfibrillar-associated protein 5	MFAP5
H7BZJ3	Protein disulfide-isomerase A3	PDIA3
H7C0V9	Amyloid-beta A4 protein	APP
I4AY87	Macrophage migration inhibitory factor	MIF
O00391	Sulfhydryl oxidase 1	QSOX1
P00338	L-Lactate dehydrogenase A chain	LDHA
P00441	Superoxide dismutase [Cu-Zn]	SOD1
P01023	Alpha-2-macroglobulin	A2M
P01024	Complement C3	C3
P01033	Metalloproteinase inhibitor 1	TIMP1
P01034	Cystatin-C	CST3
P02452	Collagen alpha-1(I) chain	COL1A1
P02461	Collagen alpha-1(III) chain	COL3A1
P02647	Apolipoprotein A-I	APOA1
P05387	60S acidic ribosomal protein P2	RPLP2
P05997	Collagen alpha-2(V) chain	COL5A2
P07585	Decorin	DCN
P07737	Profilin-1	PFN1
P07942	Laminin subunit beta-1	LAMB1
P08670	Vimentin	VIM
P09382	Galectin-1	LGALS1
P09486	SPARC	SPARC
P09871	Complement C1s subcomponent	C1S
P10599	Thioredoxin	TXN
P11047	Laminin subunit gamma-1	LAMC1
P12110	Collagen alpha-2(VI) chain	COL6A2
P14543	Nidogen-1	NID1
P18206	Vinculin	VCL
P21333	Filamin-A	FLNA
P24592	Insulin-like growth factor-binding protein 6	IGFBP6
P26022	Pentraxin-related protein PTX3	PTX3
P30041	Peroxiredoxin-6	PRDX6
P35442	Thrombospondin-2	THBS2
P35555	Fibrillin-1	FBN1
P36955	Pigment epithelium-derived factor	SERPINF1
P48061	Stromal cell-derived factor 1	CXCL12
P50454	Serpin H1	SERPINH1
P51884	Lumican	LUM
P55290	Cadherin-13	CDH13
P63261	Actin, cytoplasmic 2	ACTG1
P63313	Thymosin beta-10	TMSB10
Q01995	Transgelin	TAGLN
Q08629	Testican-1	SPOCK1
Q0Z944	Beta globin	HBB
Q12841	Follistatin-related protein 1	FSTL1
Q14767	Latent-transforming growth factor beta-binding protein 2	LTBP2
Q15582	Transforming growth factor-beta-induced protein ig-h3 1	TGFBI
Q16270	Insulin-like growth factor-binding protein 7	IGFBP7
Q16778	Histone H2B type 2-E	HIST2H2BE
Q53FA4	Cysteine-rich, angiogenic inducer, 61 variant	CYR61
Q53G99	Beta actin variant	ACTB
Q59GA0	Thy-1 cell surface antigen variant	THY1
Q5M8T4	Connective tissue growth factor	CTGF
Q6EMK4	Vasorin	VASN
Q6FHC9	STC2 protein	STC2
Q6FHW3	DF protein	CFD
Q6IAW5	CALU protein	CALU
Q6YHK3	CD109 antigen	CD109
Q8IUX7	Adipocyte enhancer-binding protein 1	AEBP1
Q99715	Collagen alpha-1(XII) chain	COL12A1
V9HWC6	Peptidyl-prolyl cis-trans isomerase	PPIB
V9HWE8	Epididymis secretory sperm binding protein Li 47e	ARHGDIA
V9HWI5	Cofilin 1 (nonmuscle)	CFL1
V9HWK1	Triosephosphate isomerase	TPI1
V9HWN7	Fructose-bisphosphate aldolase	ALDOA

**(b):** 

Identified proteins of inflammatory condition (93 proteins)		
Accession	Description	Gene symbol
A0A024R1U8	Insulin-like growth factor binding protein 4	IGFBP4
A0A024R462	Fibronectin 1	FN1
A0A024R5Z7	Annexin	ANXA2
A0A024R6R4	Matrix metallopeptidase 2	MMP2
A0A024R8V7	TIMP metallopeptidase inhibitor 2	TIMP2
A0A024R969	Chitinase 3-like 1	CHI3L1
A0A024RDA5	Multifunctional fusion protein	IL-8; CXCL8
A0A087WTA8	Collagen alpha-2(I) chain	COL1A2
A0A087X0S5	Collagen alpha-1(VI) chain	COL6A1
A0A0A0MT01	Gelsolin	GSN
A0A0S2Z3G9	Actinin alpha 4 isoform 1	ACTN4
A0A161I202	Lactoferrin	LTF
A0A1B0GU92	Uncharacterized protein	N/A
A0A1U9X7H4	CFB	CFB
A4D1W7	Inhibin, beta A (activin A, activin AB alpha polypeptide)	INHBA
A4D2D2	Procollagen C-endopeptidase enhancer	PCOLCE
A6XND1	Insulin-like growth factor binding protein 3 isoform b	IGFBP3
A8K7Q1	Nucleobindin 1	NUCB1
B2R4R0	Histone H4	HIST1H4A
B2R5J8	C-C motif chemokine	CCL5
B2RBS8	Albumin	ALB
B2RCM5	EGF-containing fibulin-like extracellular matrix protein 2	EFEMP2
B2RDW0	Calmodulin 2	CALM2
B3KQT9	Protein disulfide-isomerase	PDIA3
B4DDQ2	Biglycan	BGN
B4DLV7	Rab GDP dissociation inhibitor	GDI2
B4DMR3	Glia-derived nexin	SERPINE2
B4DPQ0	Complement C1r subcomponent	C1R
B4E324	Lysosomal protective protein	CTSA
B4E3Q1	Calsyntenin-1	CLSTN1
B5MCZ3	Interleukin-6	IL-6
D0PNI1	Epididymis luminal protein 4	YWHAZ
D3DTX7	Collagen, type I, alpha 1	COL1A1
D3YTG3	Target of Nesh-SH3	ABI3BP
D6RF92	C-X-C motif chemokine	CXCL6
D9ZGF2	Collagen, type VI, alpha 3	COL6A3
F8W6I7	Heterogeneous nuclear ribonucleoprotein A1	HNRNPA1
I4AY87	Macrophage migration inhibitory factor	MIF
O00300	Tumor necrosis factor receptor superfamily member 11B	TNFRSF11B
O00391	Sulfhydryl oxidase 1	QSOX1
P01024	Complement C3	C3
P01033	Metalloproteinase inhibitor 1	TIMP1
P01034	Cystatin-C	CST3
P02461	Collagen alpha-1(III) chain	COL3A1
P04083	Annexin A1	ANXA1
P07585	Decorin	DCN
P07737	Profilin-1	PFN1
P07996	Thrombospondin-1	THBS1
P08254	Stromelysin-1	MMP3
P08670	Vimentin	VIM
P09341	Growth-regulated alpha protein	CXCL1
P09382	Galectin-1	LGALS1
P09486	SPARC	SPARC
P09871	Complement C1s subcomponent	C1S
P10599	Thioredoxin	TXN
P11047	Laminin subunit gamma-1	LAMC1
P12110	Collagen alpha-2(VI) chain	COL6A2
P13500	C-C motif chemokine 2	CCL2
P14543	Nidogen-1	NID1
P15018	Leukemia inhibitory factor	LIF
P18206	Vinculin	VCL
P19875	C-X-C motif chemokine 2	CXCL2
P19876	C-X-C motif chemokine 3	CXCL3
P20809	Interleukin-11	IL-11
P21333	Filamin-A	FLNA
P24592	Insulin-like growth factor-binding protein 6	IGFBP6
P26022	Pentraxin-related protein PTX3	PTX3
P35442	Thrombospondin-2	THBS2
P35555	Fibrillin-1	FBN1
P48307	Tissue factor pathway inhibitor 2	TFPI2
P51884	Lumican	LUM
P62328	Thymosin beta-4	TMSB4X
P63313	Thymosin beta-10	TMSB10
P98066	Tumor necrosis factor-inducible gene 6 protein	TNFAIP6; TSG-6
Q12841	Follistatin-related protein 1	FSTL1
Q16270	Insulin-like growth factor-binding protein 7	IGFBP7
Q16778	Histone H2B type 2-E	HIST2H2BE
Q53G71	Calreticulin variant	CALR
Q53G75	Matrix metalloproteinase 1 preproprotein variant	MMP1
Q53G99	Beta actin variant	ACTB
Q53GY0	Plastin 3 variant	PLS3
Q6EMK4	Vasorin	VASN
Q6FHZ0	Malate dehydrogenase	MDH2
Q6IAW5	CALU protein	CALU
Q75MU2	Uncharacterized protein WBSCR1	EIF4H
Q86Z22	Epididymis secretory protein Li 297	SH3BGRL3
Q8IUX7	Adipocyte enhancer-binding protein 1	AEBP1
Q99715	Collagen alpha-1(XII) chain	COL12A1
V9HWB4	Epididymis secretory sperm binding protein Li 89n	HSPA5
V9HWC6	Peptidyl-prolyl cis-trans isomerase	PPIB
V9HWI5	Cofilin 1	CFL1
V9HWN7	Fructose-bisphosphate aldolase	ALDOA
V9HWP2	Epididymis luminal protein 35	HSP90B1

**Table 2:** The identification of proteins related to tissue regeneration.

Anti-inflammation	Anti-apoptosis	ECM-cell interactions	Homeostasis	Inhibition of MMPs	Regeneration of chondrocytes
Inflammatory condition					
ANXA1 [59, 60] IL-11 [61] TSP-1 [62, 64, 69] TSG-6 [6, 65, 66] NID1 [67] TMSB4X [68]	LTF [70] TMSB4X [68] LIF [71] CALR [72]	BGN [73] COL1A1 [74] IGFBP3 [75] DCN [73] LUM [73] FBN1 [63] FN1 [63, 75] VCL [76] TSP-1 [63] TSP-2 [63] TFPI2 [77]	ANXA1 [59, 60] VIM [78]	TSG-6 [6, 65, 66] IGFBP3 [75] INHBA [80] TSP-1 [62, 64, 69] TSP-2 [81, 82] SERPINE2 [91]	TSP-2 [81, 82] LTF [70] MIF [83]

Normal condition					
NID1 [67] SERPINE1 [79] A2M [84, 85]	LTF [70] ACTG1 [74] VTN [74]	CTGF [86] TGFBI [87] VTN [74] BGN [73] COL1A1 [74] IGFBP3 [75] DCN [73] LUM [73] FBN1 [63] FN1 [63, 75] VCL [76] TSP-2 [63]	VIM [78]	CYR61 [88] SOD1 [89] SPOCK1 [90] A2M [84] IGFBP3 [75] TSP-2 [81, 82]	TSP-2 [81, 82] LTF [70] MIF [83]

## Data Availability

The experimental data analysed in this study are included in this article. The data used to support the findings of this study are available from the corresponding author upon request.

## Supplementary Materials

Supplementary 1 Supplementary Figure 1: the characterizations of bone marrow (BM), adipose tissue (AT), and synovial membrane (SM) mesenchymal stem cells (MSCs). (a) Phase-contrast microscopy images of MSCs (scale bar = 100 *μ*m). (b) Differentiation potential of MSCs. Adipogenic differentiation (Oil Red O, top), osteogenic differentiation (Alizarin Red S, middle), and chondrogenic differentiation (Alcian Blue, bottom). (c) The immunophenotyping of MSCs using flow cytometry. Positive (CD73, CD90, and CD105) and negative (CD14, CD34, CD45, CD79a, and human leukocyte antigen-DR (HLA-DR)) markers. The histogram is shown with an overlay isotype control.

Supplementary 2 Supplementary Table 1: detailed information on proteins secreted by BM-, AT-, and SM-MSC under normal and inflammatory conditions, respectively.
