# Supplementary material for: Proteomic Analysis Reveals Commonly Secreted Proteins of Mesenchymal Stem Cells Derived from Bone Marrow, Adipose Tissue, and Synovial Membrane to Show Potential for Cartilage Regeneration in Knee Osteoarthritis
Source: Stem Cells Int. 2021 Jun 28;2021:6694299. doi: 10.1155/2021/6694299 (PMC8264516; doi:10.1155/2021/6694299)
Supplement: Supplementary 1 — Supplementary Figure 1: the characterizations of bone marrow (BM), adipose tissue (AT), and synovial membrane (SM) mesenchymal stem cells (MSCs). (a) Phase-contrast microscopy images of MSCs (scale bar = 100 μm). (b) Differentiation potential of MSCs. Adipogenic differentiation (Oil Red O, top), osteogenic differentiation (Alizarin Red S, middle), and chondrogenic differentiation (Alcian Blue, bottom). (c) The immunophenotyping of MSCs using flow cytometry. Positive (CD73, CD90, and CD105) and negative (CD14, CD34, CD45, CD79a, and human leukocyte antigen-DR (HLA-DR)) markers. The histogram is shown with an overlay isotype control. [file 6694299.f1.pdf]

Lee *et al.*, Fig. S1

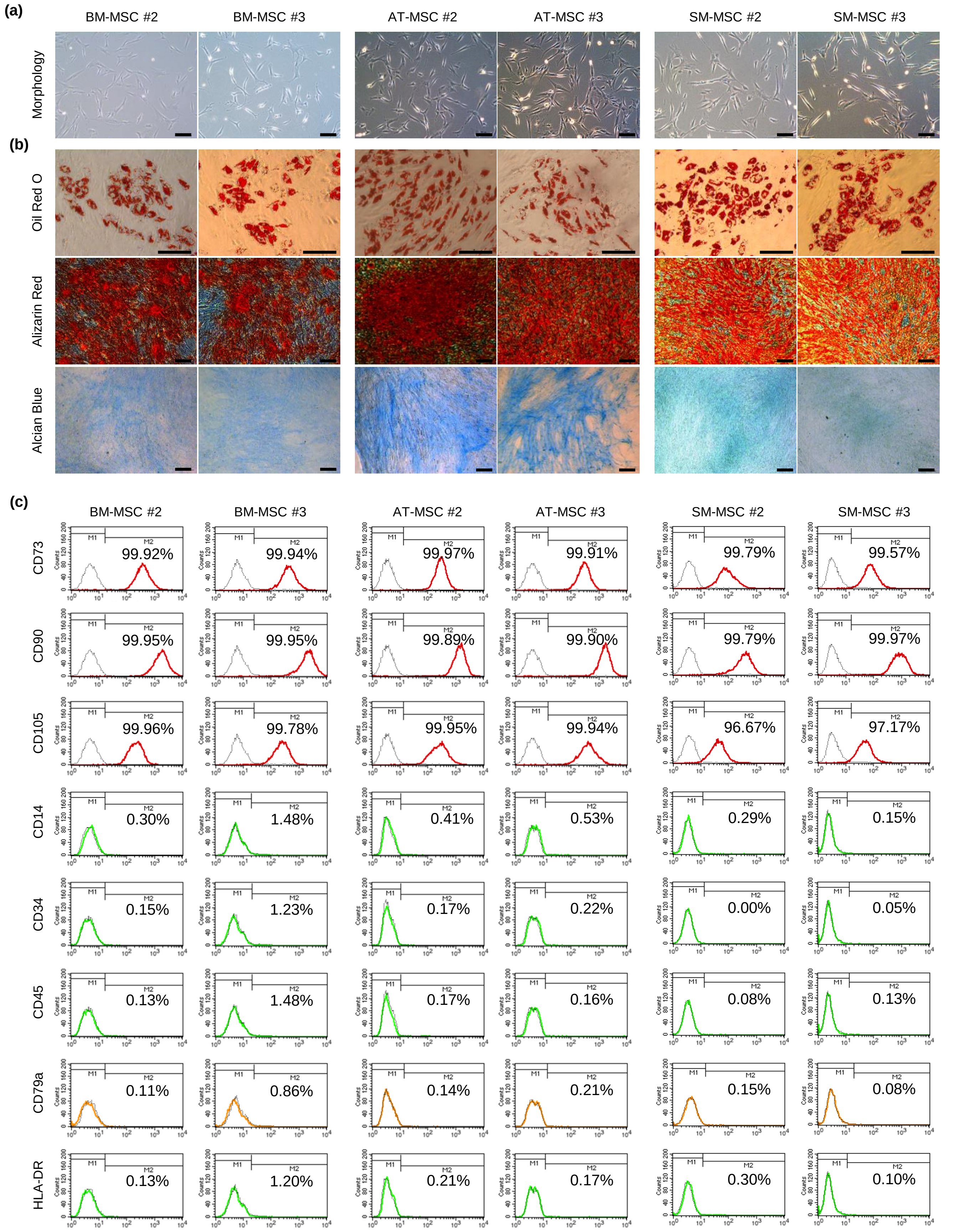

Supplementary Figure 1: The characterizations of bone marrow (BM), adipose tissue (AT), and synovial membrane (SM) mesenchymal stem cells (MSCs). (a) Phase-contrast microscopy images of MSCs (scale bar = 100 μm). (b) Differentiation potential of MSCs. Adipogenic differentiation (Oil Red O, top), osteogenic differentiation (Alizarin Red S, middle), and chondrogenic differentiation (Alcian blue, bottom). (c) The immunophenotyping of MSCs using flow cytometry. Positive (CD73, CD90, and CD105) and negative markers (CD14, CD34, CD45, CD79a, and human leukocyte antigen-DR (HLA-DR)). The histogram is shown with an overlay isotype control.
