# Supplementary material for: Proteomic Analysis Reveals Commonly Secreted Proteins of Mesenchymal Stem Cells Derived from Bone Marrow, Adipose Tissue, and Synovial Membrane to Show Potential for Cartilage Regeneration in Knee Osteoarthritis
Source: Stem Cells Int. 2021 Jun 28;2021:6694299. doi: 10.1155/2021/6694299 (PMC8264516; doi:10.1155/2021/6694299)
Supplement: Supplementary 2 — Supplementary Table 1: detailed information on proteins secreted by BM-, AT-, and SM-MSC under normal and inflammatory conditions, respectively. [file 6694299.f2.pdf]

Supplementary Table 1-1: Detailed information on commonly secreted proteins by 3 bone marrow (BM) mesenchymal stem cells (MSCs) under normal condition.

Accession	Description	Coverage [%][a]	# Peptides[b]	# PSMs[c]	# Unique Peptides[d]	# AAs[e]	MW [kDa]	Calc. pI[f]	Score Sequest HT[g]	# Peptides: Sequest HT[h]
A0A024QYT5	Serpin peptidase inhibitor, clade E (Nexin, plasminogen activator inhibitor type 1), member 1, isoform CRA_b OS=Homo sapiens OX=9606 GN=SERPINE1 PE=3 SV=1	48	13	59	13	402	45	7.2	161.94	13
A0A024QZL1	Proteoglycan 1, secretory granule, isoform CRA_a OS=Homo sapiens OX=9606 GN=PRG1 PE=4 SV=1	8	1	2	1	158	17.6	4.86	5.07	1
A0A024R0A1	Macrophage colony-stimulating factor 1 OS=Homo sapiens OX=9606 GN=CSF1 PE=4 SV=1	2	1	3	1	554	60.1	5.29	8.35	1
A0A024R0P8	Transforming growth factor, beta 1 (Camurati-Engelmann disease), isoform CRA_a OS=Homo sapiens OX=9606 GN=TGFB1 PE=3 SV=1	5	2	6	2	390	44.3	8.53	12.76	2
A0A024R1N1	Myosin, heavy polypeptide 9, non-muscle, isoform CRA_a OS=Homo sapiens OX=9606 GN=MYH9 PE=3 SV=1	22	30	72	30	1960	226.4	5.6	186.22	30
A0A024R1U8	Insulin-like growth factor binding protein 4, isoform CRA_a OS=Homo sapiens OX=9606 GN=IGFBP4 PE=4 SV=1	21	4	19	4	258	27.9	7.15	49.99	4
A0A024R1Z6	Vesicle amine transport protein 1 homolog (T californica), isoform CRA_a OS=Homo sapiens OX=9606 GN=VAT1 PE=4 SV=1	18	4	7	4	393	41.9	6.29	17.94	4
A0A024R222	Phosphoserine aminotransferase OS=Homo sapiens OX=9606 GN=PSAT1 PE=3 SV=1	12	4	6	4	370	40.4	7.66	15.12	4
A0A024R2P0	40S ribosomal protein SA OS=Homo sapiens OX=9606 GN=RPSA PE=3 SV=1	15	3	8	3	295	32.8	4.87	21.41	3
A0A024R2W4	Dystroglycan 1 (Dystrophin-associated glycoprotein 1), isoform CRA_a OS=Homo sapiens OX=9606 GN=DAG1 PE=4 SV=1	8	4	8	4	895	97.5	8.56	21.96	4
A0A024R433	Insulin-like growth factor binding protein 5, isoform CRA_a OS=Homo sapiens OX=9606 GN=IGFBP5 PE=4 SV=1	36	7	47	7	272	30.6	8.21	140.92	7
A0A024R462	Fibronectin 1, isoform CRA_n OS=Homo sapiens OX=9606 GN=FN1 PE=4 SV=1	57	83	1626	3	2355	259	5.73	5354.05	83
A0A024R5Z7	Annexin OS=Homo sapiens OX=9606 GN=ANXA2 PE=3 SV=1	40	12	39	12	339	38.6	7.75	99.1	12
A0A024R6K8	Tryptophanyl-tRNA synthetase, isoform CRA_a OS=Homo sapiens OX=9606 GN=WARS PE=3 SV=1	7	2	2	2	471	53.1	6.23	4.83	2
A0A024R6R4	Matrix metallopeptidase 2 (Gelatinase A, 72kDa gelatinase, 72kDa type IV collagenase), isoform CRA_a OS=Homo sapiens OX=9606 GN=MMP2 PE=3 SV=1	54	23	180	23	660	73.8	5.47	558.33	23
A0A024R8E5	Collagen, type V, alpha 1, isoform CRA_a OS=Homo sapiens OX=9606 GN=COL5A1 PE=4 SV=1	19	19	63	17	1838	183.4	5.06	209.65	19
A0A024R8V7	TIMP metallopeptidase inhibitor 2, isoform CRA_b OS=Homo sapiens OX=9606 GN=TIMP2 PE=4 SV=1	41	6	38	6	179	20.2	7.17	106.35	6
A0A024R969	Chitinase 3-like 1 (Cartilage glycoprotein-39), isoform CRA_a OS=Homo sapiens OX=9606 GN=CHI3L1 PE=3 SV=1	33	9	24	9	383	42.6	8.46	59.69	9
A0A024RA94	Microfibrillar-associated protein 2, isoform CRA_d OS=Homo sapiens OX=9606 GN=MFAP2 PE=4 SV=1	7	1	2	1	183	20.8	4.97	4.39	1
A0A024RAK9	Hyaluronan and proteoglycan link protein 1, isoform CRA_a OS=Homo sapiens OX=9606 GN=HAPLN1 PE=4 SV=1	27	6	20	4	354	40.1	7.42	62.6	6
A0A024RAM4	Microtubule-associated protein 1B, isoform CRA_b OS=Homo sapiens OX=9606 GN=MAP1B PE=4 SV=1	1	1	1	1	2468	270.5	4.81	1.92	1
A0A024RAQ9	Chondroitin sulfate proteoglycan 2 (Versican), isoform CRA_b OS=Homo sapiens OX=9606 GN=CSPG2 PE=4 SV=1	7	15	56	15	3396	372.6	4.51	165.93	15
A0A024RC55	Milk fat globule-EGF factor 8 protein, isoform CRA_a OS=Homo sapiens OX=9606 GN=MFGE8 PE=4 SV=1	43	11	39	11	387	43.1	8.15	108.34	11
A0A024RD80	Heat shock protein 90kDa alpha (Cytosolic), class B member 1, isoform CRA_a OS=Homo sapiens OX=9606 GN=HSP90AB1 PE=3 SV=1	10	6	9	2	724	83.2	5.03	19.27	6
A0A024RDW8	Collagen, type IV, alpha 2, isoform CRA_a OS=Homo sapiens OX=9606 GN=COL4A2 PE=4 SV=1	38	34	110	34	1712	167.4	8.66	327.45	34
A0A087WTA8	Collagen alpha-2(I) chain OS=Homo sapiens OX=9606 GN=COL1A2 PE=1 SV=1	59	43	1306	43	1364	129.1	9.01	4431.48	43
A0A087WVQ6	Clathrin heavy chain OS=Homo sapiens OX=9606 GN=CLTC PE=1 SV=1	1	2	4	2	1679	191.9	5.69	8.25	2

Accession	Description	Coverage [%][a]	# Peptides[b]	# PSMs[c]	# Unique Peptides[d]	# AAs[e]	MW [kDa]	Calc. pI[f]	Score Sequest HT[g]	# Peptides: Sequest HT[h]
A0A087X0K0	Collagen alpha-1(XV) chain OS=Homo sapiens OX=9606 GN=COL15A1 PE=1 SV=1	1	1	3	1	1374	140	4.98	6.73	1
A0A087WYD1	AP-2 complex subunit beta (Fragment) OS=Homo sapiens OX=9606 GN=AP2B1 PE=1 SV=1	3	1	1	1	639	70.9	5.06	3	1
A0A087X0S5	Collagen alpha-1(VI) chain OS=Homo sapiens OX=9606 GN=COL6A1 PE=1 SV=1	47	29	248	29	1026	108.3	5.43	802.58	29
A0A090N8G0	Glycyl-tRNA synthetase OS=Homo sapiens OX=9606 GN=GARS PE=4 SV=1	4	2	2	2	685	77.5	6.24	4.35	2
A0A090N8Y2	Protein disulfide-isomerase A4 OS=Homo sapiens OX=9606 GN=ERP70 PE=2 SV=1	2	1	3	1	645	72.9	5.07	6.74	1
A0A0A0MT01	Gelsolin OS=Homo sapiens OX=9606 GN=GSN PE=1 SV=1	19	9	20	8	767	84.7	5.83	59.11	9
A0A0A0MTS2	Glucose-6-phosphate isomerase (Fragment) OS=Homo sapiens OX=9606 GN=GPI PE=1 SV=1	5	2	4	2	573	64.8	9.04	10.05	2
A0A0F7G8J1	Plasminogen OS=Homo sapiens OX=9606 GN=PLG PE=2 SV=1	2	2	6	2	809	90.6	7.36	15.86	2
A0A0S2Z3G9	Actinin alpha 4 isoform 1 (Fragment) OS=Homo sapiens OX=9606 GN=ACTN4 PE=2 SV=1	39	27	65	17	911	104.8	5.44	165.24	27
A0A140VJI7	Testicular tissue protein Li 61 OS=Homo sapiens OX=9606 PE=2 SV=1	30	9	24	9	540	60.6	6.71	77.95	9
A0A146E5L3	Mesenchymal stromal cell-and fibroblast-expressing Linx paralogue OS=Homo sapiens OX=9606 GN=Meflin PE=2 SV=1	20	5	20	5	428	46	5.15	57.62	5
A0A161I202	Lactoferrin OS=Homo sapiens OX=9606 GN=LTF PE=2 SV=1	3	2	7	2	711	78.3	8.18	14.75	2
A0A172Q381	Endosialin (Fragment) OS=Homo sapiens OX=9606 GN=TEM1 PE=2 SV=1	7	2	4	2	668	71.2	5.01	10.9	2
A0A1B0GU92	Uncharacterized protein OS=Homo sapiens OX=9606 PE=1 SV=1	4	2	3	2	557	59.8	6.62	6.77	2
A0A1B0GUS4	Ubiquitin-conjugating enzyme E2 L5 OS=Homo sapiens OX=9606 GN=UBE2L5 PE=2 SV=1	26	2	2	2	154	17.9	7.84	5.02	2
A0A2R8Y6G6	Alpha-enolase OS=Homo sapiens OX=9606 GN=ENO1 PE=1 SV=1	44	11	29	11	434	47.3	6.99	86.4	11
A0A2U3TZL5	CD59 glycoprotein (Fragment) OS=Homo sapiens OX=9606 GN=CD59 PE=4 SV=1	10	1	3	1	120	13.3	6.25	7.82	1
A1KY36	Cell proliferation-inducing protein 41 OS=Homo sapiens OX=9606 PE=2 SV=1	26	9	20	9	745	73.4	9.61	58.17	9
A1L4H1	Soluble scavenger receptor cysteine-rich domain-containing protein SSC5D OS=Homo sapiens OX=9606 GN=SSC5D PE=1 SV=3	6	6	12	6	1573	165.6	6.13	26.99	6
A3KC71	Nuclear envelope protein okuribin OS=Homo sapiens OX=9606 GN=okuribin PE=2 SV=1	25	18	53	18	950	108.1	9.72	156.78	18
A3KPE2	Apolipoprotein C-III OS=Homo sapiens OX=9606 GN=APOC3 PE=2 SV=1	16	1	3	1	99	10.8	5.41	7.18	1
A4D1M3	Carboxypeptidase A4 OS=Homo sapiens OX=9606 GN=CPA4 PE=2 SV=1	10	3	4	3	421	47.3	6.7	8.98	3
A4D1W7	Inhibin, beta A (Activin A, activin AB alpha polypeptide) OS=Homo sapiens OX=9606 GN=INHBA PE=2 SV=1	41	14	51	14	426	47.4	8.03	155.99	14
A4D2D2	Procollagen C-endopeptidase enhancer OS=Homo sapiens OX=9606 GN=PCOLCE PE=4 SV=1	49	15	53	15	449	47.9	7.43	155.32	15
A5PLM9	Cathepsin L1 OS=Homo sapiens OX=9606 GN=CTSL1 PE=2 SV=1	4	1	2	1	333	37.5	5.45	4.32	1
A6XMH5	Beta-2-microglobulin OS=Homo sapiens OX=9606 PE=2 SV=1	24	1	6	1	92	10.4	8.02	21.21	1
A6XND1	Insulin-like growth factor binding protein 3 isoform b OS=Homo sapiens OX=9606 GN=IGFBP3 PE=1 SV=1	38	7	34	7	263	29	8.46	91.18	7
A8K2H4	cDNA FLJ78235 OS=Homo sapiens OX=9606 PE=2 SV=1	26	6	17	6	339	37.8	6.3	44.85	6

Accession	Description	Coverage [%][a]	# Peptides[b]	# PSMs[c]	# Unique Peptides[d]	# AAs[e]	MW [kDa]	Calc. pI[f]	Score Sequest HT[g]	# Peptides: Sequest HT[h]
A8K2Q6	Peptidyl-prolyl cis-trans isomerase OS=Homo sapiens OX=9606 PE=2 SV=1	6	1	1	1	212	22.7	8.4	2.15	1
A8K3K1	cDNA FLJ78096, highly similar to Homo sapiens actin, alpha, cardiac muscle (ACTC), mRNA OS=Homo sapiens OX=9606 PE=2 SV=1	20	6	29	2	377	42	5.39	73.41	6
A8K3Q2	cDNA FLJ75319, highly similar to Homo sapiens ADAMTS-like 1 (ADAMTSL1), transcript variant 2, mRNA OS=Homo sapiens OX=9606 PE=2 SV=1	11	4	6	4	525	58.4	6.57	14.14	4
A8K491	cDNA FLJ76037, highly similar to Homo sapiens matrilin 3 (MATN3), mRNA OS=Homo sapiens OX=9606 PE=2 SV=1	3	1	2	1	486	52.8	6.7	4.35	1
A8K6C9	cDNA FLJ78037, highly similar to Homo sapiens insulin-like growth factor 2 (somatomedin A), mRNA OS=Homo sapiens OX=9606 PE=2 SV=1	14	2	4	2	180	20.2	9.32	8.33	2
A8K7Q1	cDNA FLJ77770, highly similar to Homo sapiens nucleobindin 1 (NUCB1), mRNA OS=Homo sapiens OX=9606 PE=2 SV=1	36	11	36	11	461	53.9	5.25	98.37	11
A8K7T4	cDNA FLJ75774, highly similar to Homo sapiens lectin, mannose-binding 2 (LMAN2), mRNA OS=Homo sapiens OX=9606 PE=2 SV=1	8	2	3	2	356	40.2	6.95	8.14	2
A8KAJ3	cDNA FLJ77823, highly similar to Homo sapiens EGF-containing fibulin-like extracellular matrix protein 1, transcript variant 3, mRNA OS=Homo sapiens OX=9606 PE=2 SV=1	36	11	38	11	493	54.6	5.14	110.35	11
B1AKZ4	Phosphoprotein enriched in astrocytes 15, isoform CRA_a OS=Homo sapiens OX=9606 GN=PEA15 PE=2 SV=1	9	1	3	1	130	15	5.02	7.65	1
B2R582	cDNA, FLJ92374, highly similar to Homo sapiens C-type lectin domain family 3, member B (CLEC3B), mRNA OS=Homo sapiens OX=9606 PE=2 SV=1	41	7	21	1	202	22.5	6.04	53.32	7
B2R5H0	Protein S100 OS=Homo sapiens OX=9606 PE=2 SV=1	15	1	4	1	105	11.7	7.18	10.77	1
B2R5M9	cDNA, FLJ92537, highly similar to Homo sapiens procollagen-lysine, 2-oxoglutarate 5-dioxygenase (lysine hydroxylase, Ehlers-Danlos syndrome type VI) (PLOD), mRNA OS=Homo sapiens OX=9606 PE=2 SV=1	17	9	23	9	727	83.5	7.02	56.51	9
B2R7T8	cDNA, FLJ93598, highly similar to Homo sapiens capping protein (actin filament) muscle Z-line, beta (CAPZB), mRNA OS=Homo sapiens OX=9606 PE=2 SV=1	10	2	4	2	272	30.6	6	9.07	2
B2RBS8	cDNA, FLJ95666, highly similar to Homo sapiens albumin (ALB), mRNA OS=Homo sapiens OX=9606 PE=2 SV=1	2	2	20	2	609	69.3	6.28	55.23	2
B2RCM5	cDNA, FLJ96160, highly similar to Homo sapiens EGF-containing fibulin-like extracellular matrix protein 2 (EFEMP2), mRNA OS=Homo sapiens OX=9606 PE=2 SV=1	26	8	18	8	443	49.3	4.94	46.16	8
B2RDW0	cDNA, FLJ96792, highly similar to Homo sapiens calmodulin 2 (phosphorylase kinase, delta) (CALM2), mRNA OS=Homo sapiens OX=9606 PE=2 SV=1	21	2	4	2	149	16.8	4.22	12.4	2
B3KQQ9	cDNA PSEC0048 fis, clone NT2RP2000028, highly similar to Serine protease 23 OS=Homo sapiens OX=9606 PE=2 SV=1	25	5	14	5	383	43	9.36	39.99	5
B3KQT9	Protein disulfide-isomerase OS=Homo sapiens OX=9606 PE=2 SV=1	18	6	11	6	480	54.1	7.21	25.16	6
B3KY04	cDNA FLJ46506 fis, clone THYMU3030752, highly similar to BTB/POZ domain-containing protein KCTD12 OS=Homo sapiens OX=9606 PE=2 SV=1	22	5	5	5	325	35.7	5.64	10.44	5
B4DDQ2	Biglycan OS=Homo sapiens OX=9606 PE=2 SV=1	55	13	118	13	334	37.6	7.97	363.13	13
B4DE78	cDNA FLJ52141, highly similar to 14-3-3 protein gamma OS=Homo sapiens OX=9606 PE=2 SV=1	12	2	3	1	207	23.5	4.82	8.03	2
B4DF70	cDNA FLJ60461, highly similar to Peroxiredoxin-2 OS=Homo sapiens OX=9606 PE=2 SV=1	21	3	6	2	183	20.1	8.78	13.4	3
B4DFL3	cDNA FLJ56661, highly similar to Proteasome subunit beta type 4 OS=Homo sapiens OX=9606 PE=2 SV=1	13	1	2	1	167	18.3	9.35	5.74	1
B4DJ30	cDNA FLJ61290, highly similar to Neutral alpha-glucosidase AB OS=Homo sapiens OX=9606 PE=2 SV=1	6	4	4	4	995	112.9	6.06	9.26	4
B4DJI4	cDNA FLJ58065, highly similar to LIM and SH3 domain protein 1 OS=Homo sapiens OX=9606 PE=2 SV=1	6	1	2	1	220	24.5	8.54	3.93	1
B4DKJ4	cDNA FLJ57738, highly similar to Translationally-controlled tumor protein OS=Homo sapiens OX=9606 PE=2 SV=1	10	1	1	1	142	16.1	4.68	2.47	1
B4DKZ9	cDNA FLJ55705, highly similar to Threonyl-tRNA synthetase, cytoplasmic OS=Homo sapiens OX=9606 PE=2 SV=1	2	1	1	1	602	70.3	8.56	2.41	1

Accession	Description	Coverage [%][a]	# Peptides[b]	# PSMs[c]	# Unique Peptides[d]	# AAs[e]	MW [kDa]	Calc. pI[f]	Score Sequest HT[g]	# Peptides: Sequest HT[h]
B4DLV7	Rab GDP dissociation inhibitor OS=Homo sapiens OX=9606 PE=2 SV=1	34	10	15	10	449	51.1	8.18	37.21	10
B4DM79	cDNA FLJ53848, highly similar to Inter-alpha-trypsin inhibitor heavy chain H2 OS=Homo sapiens OX=9606 PE=2 SV=1	8	4	8	4	543	60.7	5.35	19.91	4
B4DNG0	cDNA FLJ58142, highly similar to Olfactomedin-like protein 3 OS=Homo sapiens OX=9606 GN=OLFML3 PE=1 SV=1	30	7	18	7	345	39	6.11	43.42	7
B4DNH1	Profilin OS=Homo sapiens OX=9606 PE=2 SV=1	7	1	2	1	188	20.8	7.83	5.85	1
B4DPH4	cDNA FLJ58778, highly similar to Plasminogen OS=Homo sapiens OX=9606 PE=2 SV=1	4	1	3	1	407	44.9	7.46	9.41	1
B4DPQ0	Complement C1r subcomponent OS=Homo sapiens OX=9606 GN=C1R PE=1 SV=1	44	19	71	19	719	81.8	6.37	229.77	19
B4DPZ5	cDNA FLJ53495, highly similar to Polymerase I and transcript release factor OS=Homo sapiens OX=9606 PE=2 SV=1	8	2	3	2	363	40.5	5.25	6.78	2
B4DRV4	cDNA FLJ55667, highly similar to Secreted protein acidic and rich in cysteine OS=Homo sapiens OX=9606 PE=2 SV=1	70	13	326	1	212	24.7	5.62	1183.62	13
B4DU16	cDNA FLJ54550, highly similar to Homo sapiens fibronectin 1 (FN1), transcript variant 6, mRNA OS=Homo sapiens OX=9606 PE=2 SV=1	68	40	716	2	894	98.1	6.24	2346.26	40
B4DU62	cDNA FLJ54154, highly similar to EH-domain-containing protein 2 OS=Homo sapiens OX=9606 PE=2 SV=1	6	1	2	1	185	20.6	5.08	4.96	1
B4DUQ1	cDNA FLJ54552, highly similar to Heterogeneous nuclear ribonucleoprotein K OS=Homo sapiens OX=9606 PE=2 SV=1	7	2	3	2	439	48.5	5.92	6.12	2
B4DWC4	Chloride intracellular channel protein OS=Homo sapiens OX=9606 PE=2 SV=1	22	4	13	4	245	27.8	6.38	33.05	4
B4E1N8	cDNA FLJ51183, highly similar to Extracellular matrix protein 2 OS=Homo sapiens OX=9606 PE=2 SV=1	5	3	8	3	618	70.7	6.04	19.78	3
B4E3Q1	cDNA FLJ61580, highly similar to Calsyntenin-1 OS=Homo sapiens OX=9606 PE=2 SV=1	14	9	22	9	962	107.7	4.97	63.39	9
B7Z1Y2	Fructose-bisphosphate aldolase OS=Homo sapiens OX=9606 PE=2 SV=1	15	2	5	1	345	37.5	7.12	13.79	2
C9JIZ6	Prosaposin OS=Homo sapiens OX=9606 GN=PSAP PE=1 SV=2	7	3	8	3	527	58.4	5.17	19.93	3
D0PNI1	Epididymis luminal protein 4 OS=Homo sapiens OX=9606 GN=YWHAZ PE=2 SV=1	29	5	16	4	245	27.7	4.79	37.84	5
D0PNI2	Lysyl oxidase OS=Homo sapiens OX=9606 GN=LOX PE=4 SV=1	30	8	24	8	417	46.9	8.09	63.7	8
D1MGQ2	Alpha-2 globin chain OS=Homo sapiens OX=9606 GN=HBA2 PE=3 SV=1	35	4	18	4	142	15.2	8.68	46.14	4
D3DT71	Collagen, type XI, alpha 1, isoform CRA_b OS=Homo sapiens OX=9606 GN=COL11A1 PE=4 SV=1	7	6	20	4	1767	176.5	5.38	59.26	6
D3DTX7	Collagen, type I, alpha 1, isoform CRA_a OS=Homo sapiens OX=9606 GN=COL1A1 PE=4 SV=1	64	32	489	2	885	84.7	6.24	1620.85	32
D3YTG3	Target of Nesh-SH3 OS=Homo sapiens OX=9606 GN=ABI3BP PE=1 SV=1	6	8	18	8	1777	195.2	9.73	46.01	8
D6RER5	Septin-11 OS=Homo sapiens OX=9606 GN=SEPT11 PE=1 SV=1	3	1	3	1	432	49.8	6.68	6.36	1
D6RF35	Vitamin D-binding protein OS=Homo sapiens OX=9606 GN=GC PE=1 SV=1	8	2	8	2	476	53	5.52	24.26	2
D6RH06	PDZ and LIM domain protein 7 (Fragment) OS=Homo sapiens OX=9606 GN=PDLIM7 PE=1 SV=1	4	1	1	1	287	30.5	9.25	2.02	1
D9ZGF2	Collagen, type VI, alpha 3 OS=Homo sapiens OX=9606 GN=COL6A3 PE=4 SV=1	34	68	204	68	3177	343.5	6.68	563.85	68
D9ZGG2	Vitronectin OS=Homo sapiens OX=9606 GN=VTN PE=4 SV=1	3	1	3	1	478	54.3	5.8	7.57	1
E7ETU9	Procollagen-lysine,2-oxoglutarate 5-dioxygenase 2 OS=Homo sapiens OX=9606 GN=PLOD2 PE=1 SV=1	2	1	2	1	703	81.1	6.65	4.23	1

Accession	Description	Coverage [%][a]	# Peptides[b]	# PSMs[c]	# Unique Peptides[d]	# AAs[e]	MW [kDa]	Calc. pI[f]	Score Sequest HT[g]	# Peptides: Sequest HT[h]
E9PDU6	Calponin (Fragment) OS=Homo sapiens OX=9606 GN=CNN3 PE=1 SV=1	6	1	3	1	185	20.2	9.42	6.34	1
E9PHK0	Tetranectin OS=Homo sapiens OX=9606 GN=CLEC3B PE=1 SV=1	52	7	19	1	160	17.8	5.05	46.92	7
E9PL57	NEDD8-MDP1 readthrough (Fragment) OS=Homo sapiens OX=9606 GN=NEDD8-MDP1 PE=4 SV=1	8	1	1	1	170	19.5	7.43	1.94	1
E9PRJ8	Tetraspanin (Fragment) OS=Homo sapiens OX=9606 GN=CD81 PE=1 SV=1	21	2	3	2	209	22.5	6.15	10.37	2
F5H365	Protein transport protein SEC23 OS=Homo sapiens OX=9606 GN=SEC23A PE=1 SV=1	2	1	1	1	736	82.9	7.46	2.76	1
F6RFD5	Destrin OS=Homo sapiens OX=9606 GN=DSTN PE=1 SV=1	26	3	7	3	135	15.4	8.59	16.72	3
F6WIT2	Serine/threonine-protein phosphatase 2A activator OS=Homo sapiens OX=9606 GN=PTPA PE=1 SV=1	3	1	1	1	288	32.9	6.93	2.05	1
F8VR42	Dynein regulatory complex subunit 2 (Fragment) OS=Homo sapiens OX=9606 GN=CCDC65 PE=1 SV=1	3	1	3	1	373	44	9.25	5.78	1
F8W6I7	Heterogeneous nuclear ribonucleoprotein A1 OS=Homo sapiens OX=9606 GN=HNRNPA1 PE=1 SV=2	3	1	3	1	307	33.1	9.13	7.46	1
G3V3A0	Alpha-1-antichymotrypsin OS=Homo sapiens OX=9606 GN=SERPINA3 PE=1 SV=1	5	1	1	1	205	23.4	5.64	2.1	1
G3V3E8	NPC intracellular cholesterol transporter 2 OS=Homo sapiens OX=9606 GN=NPC2 PE=1 SV=1	22	2	6	2	174	19.2	8.44	14.37	2
G3V4U0	Fibulin-5 OS=Homo sapiens OX=9606 GN=FBLN5 PE=1 SV=1	5	2	2	2	453	50.8	4.77	3.86	2
H0UI49	Laminin, alpha 4, isoform CRA_b OS=Homo sapiens OX=9606 GN=LAMA4 PE=4 SV=1	11	13	30	13	1816	201.7	6.3	78.78	13
H0Y8C6	Importin-5 (Fragment) OS=Homo sapiens OX=9606 GN=IPO5 PE=1 SV=1	4	3	5	3	1099	123.8	5.06	11.19	3
H0YEK9	UPF0598 protein C8orf82 (Fragment) OS=Homo sapiens OX=9606 GN=C8orf82 PE=4 SV=1	14	1	1	1	134	13.8	10.92	2.2	1
H0YGS3	Microfibrillar-associated protein 5 (Fragment) OS=Homo sapiens OX=9606 GN=MFAP5 PE=1 SV=1	34	2	9	2	79	9.4	9.47	25.69	2
H3BT71	RNA-binding motif protein, X chromosome OS=Homo sapiens OX=9606 GN=RBMX PE=1 SV=1	5	1	2	1	296	32.2	9.91	3.92	1
H6VRF8	Keratin 1 OS=Homo sapiens OX=9606 GN=KRT1 PE=3 SV=1	9	5	9	5	644	66	8.12	19.26	5
H7BZJ3	Protein disulfide-isomerase A3 (Fragment) OS=Homo sapiens OX=9606 GN=PDIA3 PE=1 SV=1	11	1	3	1	123	13.5	7.3	6.48	1
H7C0V9	Amyloid-beta A4 protein (Fragment) OS=Homo sapiens OX=9606 GN=APP PE=1 SV=1	4	1	1	1	485	55.1	4.82	2.42	1
I3L1J9	Tumor necrosis factor receptor superfamily member 12A OS=Homo sapiens OX=9606 GN=TNFRSF12A PE=1 SV=1	25	1	2	1	80	8.7	8.31	8.72	1
I3L397	Eukaryotic translation initiation factor 5A (Fragment) OS=Homo sapiens OX=9606 GN=EIF5A PE=1 SV=8	37	3	3	3	146	16	5	7.92	3
I4AY87	Macrophage migration inhibitory factor (Fragment) OS=Homo sapiens OX=9606 GN=MIF PE=1 SV=1	18	2	6	2	115	12.5	7.88	14.83	2
K7ENT6	Tropomyosin alpha-4 chain OS=Homo sapiens OX=9606 GN=TPM4 PE=1 SV=2	26	7	29	1	247	28.5	4.63	73.53	7
K9JA46	Epididymis luminal secretory protein 52 OS=Homo sapiens OX=9606 GN=EL52 PE=2 SV=1	11	5	8	2	732	84.6	5.02	17.52	5
M0R261	6-phosphogluconolactonase (Fragment) OS=Homo sapiens OX=9606 GN=PGLS PE=1 SV=1	13	2	2	2	216	23	5.86	4.71	2
M1VE83	Tyrosine-protein kinase receptor OS=Homo sapiens OX=9606 GN=SDC4-ROS1_S4;R34 PE=2 SV=1	2	1	2	1	643	71.6	4.81	4.77	1
O00245	EXTL2 (Fragment) OS=Homo sapiens OX=9606 GN=EXTL2 PE=2 SV=1	9	1	1	1	129	14.7	8.43	1.93	1

Accession	Description	Coverage [%][a]	# Peptides[b]	# PSMs[c]	# Unique Peptides[d]	# AAs[e]	MW [kDa]	Calc. pI[f]	Score Sequest HT[g]	# Peptides: Sequest HT[h]
O00300	Tumor necrosis factor receptor superfamily member 11B OS=Homo sapiens OX=9606 GN=TNFRSF11B PE=1 SV=3	15	4	8	4	401	46	8.29	18.01	4
O00391	Sulfhydryl oxidase 1 OS=Homo sapiens OX=9606 GN=QSOX1 PE=1 SV=3	44	24	81	24	747	82.5	8.92	256.16	24
O00462	Beta-mannosidase OS=Homo sapiens OX=9606 GN=MANBA PE=2 SV=3	1	1	1	1	879	100.8	5.52	1.96	1
O00468	Agrin OS=Homo sapiens OX=9606 GN=AGRN PE=1 SV=6	3	4	8	4	2068	217.2	6.39	17.4	4
O14950	Myosin regulatory light chain 12B OS=Homo sapiens OX=9606 GN=MYL12B PE=1 SV=2	28	4	9	1	172	19.8	4.84	22.04	4
O15511	Actin-related protein 2/3 complex subunit 5 OS=Homo sapiens OX=9606 GN=ARPC5 PE=1 SV=3	21	2	4	2	151	16.3	5.67	8.83	2
O43854	EGF-like repeat and discoidin I-like domain-containing protein 3 OS=Homo sapiens OX=9606 GN=EDIL3 PE=1 SV=1	20	6	11	6	480	53.7	7.28	27.64	6
O60565	Gremlin-1 OS=Homo sapiens OX=9606 GN=GREM1 PE=1 SV=1	7	1	1	1	184	20.7	9.39	2.28	1
O60568	Procollagen-lysine,2-oxoglutarate 5-dioxygenase 3 OS=Homo sapiens OX=9606 GN=PLOD3 PE=1 SV=1	10	5	8	5	738	84.7	6.05	18.91	5
O60664	Perilipin-3 OS=Homo sapiens OX=9606 GN=PLIN3 PE=1 SV=3	11	3	7	3	434	47	5.44	15.5	3
O75095	Multiple epidermal growth factor-like domains protein 6 OS=Homo sapiens OX=9606 GN=MEGF6 PE=1 SV=4	4	4	10	1	1541	161.1	6.38	29.4	4
O75326	Semaphorin-7A OS=Homo sapiens OX=9606 GN=SEMA7A PE=1 SV=1	11	4	11	4	666	74.8	7.64	30.33	4
O95450	A disintegrin and metalloproteinase with thrombospondin motifs 2 OS=Homo sapiens OX=9606 GN=ADAMTS2 PE=2 SV=2	4	3	7	3	1211	134.7	7.15	17.76	3
P00338	L-lactate dehydrogenase A chain OS=Homo sapiens OX=9606 GN=LDHA PE=1 SV=2	29	7	18	7	332	36.7	8.27	45.05	7
P00441	Superoxide dismutase [Cu-Zn] OS=Homo sapiens OX=9606 GN=SOD1 PE=1 SV=2	23	2	5	2	154	15.9	6.13	18.54	2
P01008	Antithrombin-III OS=Homo sapiens OX=9606 GN=SERPINC1 PE=1 SV=1	2	1	3	1	464	52.6	6.71	7.48	1
P01023	Alpha-2-macroglobulin OS=Homo sapiens OX=9606 GN=A2M PE=1 SV=3	2	3	7	2	1474	163.2	6.46	16.46	3
P01024	Complement C3 OS=Homo sapiens OX=9606 GN=C3 PE=1 SV=2	5	6	13	6	1663	187	6.4	28.86	6
P01033	Metalloproteinase inhibitor 1 OS=Homo sapiens OX=9606 GN=TIMP1 PE=1 SV=1	61	8	67	8	207	23.2	8.1	219.81	8
P01034	Cystatin-C OS=Homo sapiens OX=9606 GN=CST3 PE=1 SV=1	56	6	30	6	146	15.8	8.75	84.86	6
P02452	Collagen alpha-1(I) chain OS=Homo sapiens OX=9606 GN=COL1A1 PE=1 SV=5	57	44	572	14	1464	138.9	5.8	1865.96	44
P02461	Collagen alpha-1(III) chain OS=Homo sapiens OX=9606 GN=COL3A1 PE=1 SV=4	30	24	98	24	1466	138.5	6.61	294.53	24
P02462	Collagen alpha-1(IV) chain OS=Homo sapiens OX=9606 GN=COL4A1 PE=1 SV=4	11	9	33	9	1669	160.5	8.28	96.41	9
P02545	Prelamin-A/C OS=Homo sapiens OX=9606 GN=LMNA PE=1 SV=1	30	15	48	15	664	74.1	7.02	126.85	15
P02647	Apolipoprotein A-I OS=Homo sapiens OX=9606 GN=APOA1 PE=1 SV=1	6	1	3	1	267	30.8	5.76	10.35	1
P04083	Annexin A1 OS=Homo sapiens OX=9606 GN=ANXA1 PE=1 SV=2	34	8	11	8	346	38.7	7.02	27.91	8
P04085	Platelet-derived growth factor subunit A OS=Homo sapiens OX=9606 GN=PDGFA PE=1 SV=1	9	1	2	1	211	24	9.39	4.22	1
P04792	Heat shock protein beta-1 OS=Homo sapiens OX=9606 GN=HSPB1 PE=1 SV=2	23	2	4	2	205	22.8	6.4	10.44	2

Accession	Description	Coverage [%][a]	# Peptides[b]	# PSMs[c]	# Unique Peptides[d]	# AAs[e]	MW [kDa]	Calc. pI[f]	Score Sequest HT[g]	# Peptides: Sequest HT[h]
P05387	60S acidic ribosomal protein P2 OS=Homo sapiens OX=9606 GN=RPLP2 PE=1 SV=1	70	4	5	4	115	11.7	4.54	11.72	4
P05997	Collagen alpha-2(V) chain OS=Homo sapiens OX=9606 GN=COL5A2 PE=1 SV=3	37	29	83	29	1499	144.8	6.46	227.45	29
P07585	Decorin OS=Homo sapiens OX=9606 GN=DCN PE=1 SV=1	45	13	87	13	359	39.7	8.54	258.75	13
P07737	Profilin-1 OS=Homo sapiens OX=9606 GN=PFN1 PE=1 SV=2	40	4	20	4	140	15	8.27	52.31	4
P07942	Laminin subunit beta-1 OS=Homo sapiens OX=9606 GN=LAMB1 PE=1 SV=2	15	16	30	16	1786	197.9	4.94	71.58	16
P08670	Vimentin OS=Homo sapiens OX=9606 GN=VIM PE=1 SV=4	42	20	103	20	466	53.6	5.12	284.53	20
P09382	Galectin-1 OS=Homo sapiens OX=9606 GN=LGALS1 PE=1 SV=2	35	3	26	3	135	14.7	5.5	75.92	3
P09486	SPARC OS=Homo sapiens OX=9606 GN=SPARC PE=1 SV=1	56	14	376	2	303	34.6	4.84	1328.13	14
P09496-2	Isoform Non-brain of Clathrin light chain A OS=Homo sapiens OX=9606 GN=CLTA	10	1	1	1	218	23.6	4.53	1.95	1
P09871	Complement C1s subcomponent OS=Homo sapiens OX=9606 GN=C1S PE=1 SV=1	40	17	53	17	688	76.6	4.96	148.73	17
P10599	Thioredoxin OS=Homo sapiens OX=9606 GN=TXN PE=1 SV=3	23	2	6	2	105	11.7	4.92	13.29	2
P10909	Clusterin OS=Homo sapiens OX=9606 GN=CLU PE=1 SV=1	26	10	33	5	449	52.5	6.27	87.79	10
P11047	Laminin subunit gamma-1 OS=Homo sapiens OX=9606 GN=LAMC1 PE=1 SV=3	20	22	48	22	1609	177.5	5.12	123.7	22
P12110	Collagen alpha-2(VI) chain OS=Homo sapiens OX=9606 GN=COL6A2 PE=1 SV=4	26	18	58	3	1019	108.5	6.21	163.68	18
P13489	Ribonuclease inhibitor OS=Homo sapiens OX=9606 GN=RNH1 PE=1 SV=2	15	5	6	5	461	49.9	4.82	15.15	5
P13639	Elongation factor 2 OS=Homo sapiens OX=9606 GN=EEF2 PE=1 SV=4	7	5	7	5	858	95.3	6.83	15.06	5
P14543	Nidogen-1 OS=Homo sapiens OX=9606 GN=NID1 PE=1 SV=3	16	12	27	12	1247	136.3	5.29	71.69	12
P15291	Beta-1,4-galactosyltransferase 1 OS=Homo sapiens OX=9606 GN=B4GALT1 PE=1 SV=5	20	4	14	4	398	43.9	8.65	36.2	4
P18065	Insulin-like growth factor-binding protein 2 OS=Homo sapiens OX=9606 GN=IGFBP2 PE=1 SV=2	38	8	40	8	325	34.8	7.5	121.86	8
P18206	Vinculin OS=Homo sapiens OX=9606 GN=VCL PE=1 SV=4	23	20	45	20	1134	123.7	5.66	110.67	20
P19021	Peptidyl-glycine alpha-amidating monooxygenase OS=Homo sapiens OX=9606 GN=PAM PE=1 SV=2	5	2	3	2	973	108.3	6.42	8.17	2
P19022	Cadherin-2 OS=Homo sapiens OX=9606 GN=CDH2 PE=1 SV=4	11	7	13	7	906	99.7	4.81	30.8	7
P19320	Vascular cell adhesion protein 1 OS=Homo sapiens OX=9606 GN=VCAM1 PE=1 SV=1	12	7	13	7	739	81.2	5.22	32.85	7
P21333	Filamin-A OS=Homo sapiens OX=9606 GN=FLNA PE=1 SV=4	35	58	169	55	2647	280.6	6.06	476.01	58
P21781	Fibroblast growth factor 7 OS=Homo sapiens OX=9606 GN=FGF7 PE=1 SV=1	5	1	1	1	194	22.5	9.14	2.3	1
P23142	Fibulin-1 OS=Homo sapiens OX=9606 GN=FBLN1 PE=1 SV=4	30	13	41	13	703	77.2	5.22	113.42	13
P23526	Adenosylhomocysteinase OS=Homo sapiens OX=9606 GN=AHCY PE=1 SV=4	11	4	8	4	432	47.7	6.34	17.72	4
P24592	Insulin-like growth factor-binding protein 6 OS=Homo sapiens OX=9606 GN=IGFBP6 PE=1 SV=1	18	3	12	3	240	25.3	7.81	40.35	3

Accession	Description	Coverage [%][a]	# Peptides[b]	# PSMs[c]	# Unique Peptides[d]	# AAs[e]	MW [kDa]	Calc. pI[f]	Score Sequest HT[g]	# Peptides: Sequest HT[h]
P24844	Myosin regulatory light polypeptide 9 OS=Homo sapiens OX=9606 GN=MYL9 PE=1 SV=4	40	5	9	2	172	19.8	4.92	22.53	5
P26022	Pentraxin-related protein PTX3 OS=Homo sapiens OX=9606 GN=PTX3 PE=1 SV=3	39	11	51	11	381	41.9	5.01	142.85	11
P26038	Moesin OS=Homo sapiens OX=9606 GN=MSN PE=1 SV=3	26	11	21	8	577	67.8	6.4	49.42	11
P26641	Elongation factor 1-gamma OS=Homo sapiens OX=9606 GN=EEF1G PE=1 SV=3	6	2	5	2	437	50.1	6.67	12.22	2
P27816	Microtubule-associated protein 4 OS=Homo sapiens OX=9606 GN=MAP4 PE=1 SV=3	4	3	3	3	1152	120.9	5.43	6.68	3
P28072	Proteasome subunit beta type-6 OS=Homo sapiens OX=9606 GN=PSMB6 PE=1 SV=4	5	1	1	1	239	25.3	4.92	2.31	1
P29692	Elongation factor 1-delta OS=Homo sapiens OX=9606 GN=EEF1D PE=1 SV=5	9	1	1	1	281	31.1	5.01	2.95	1
P30040	Endoplasmic reticulum resident protein 29 OS=Homo sapiens OX=9606 GN=ERP29 PE=1 SV=4	5	1	1	1	261	29	7.31	1.91	1
P30041	Peroxiredoxin-6 OS=Homo sapiens OX=9606 GN=PRDX6 PE=1 SV=3	14	2	4	2	224	25	6.38	9.36	2
P30044	Peroxiredoxin-5, mitochondrial OS=Homo sapiens OX=9606 GN=PRDX5 PE=1 SV=4	13	2	4	2	214	22.1	8.7	8.68	2
P30046	D-dopachrome decarboxylase OS=Homo sapiens OX=9606 GN=DDT PE=1 SV=3	12	1	1	1	118	12.7	7.3	2.35	1
P30050	60S ribosomal protein L12 OS=Homo sapiens OX=9606 GN=RPL12 PE=1 SV=1	28	3	4	3	165	17.8	9.42	8.92	3
P30530	Tyrosine-protein kinase receptor UFO OS=Homo sapiens OX=9606 GN=AXL PE=1 SV=4	6	4	7	4	894	98.3	5.39	17.28	4
P31946	14-3-3 protein beta/alpha OS=Homo sapiens OX=9606 GN=YWHAB PE=1 SV=3	17	3	6	2	246	28.1	4.83	13.5	3
P35052	Glypican-1 OS=Homo sapiens OX=9606 GN=GPC1 PE=1 SV=2	19	7	16	7	558	61.6	7.3	46.56	7
P35442	Thrombospondin-2 OS=Homo sapiens OX=9606 GN=THBS2 PE=1 SV=2	31	24	73	22	1172	129.9	4.83	201.73	24
P35555	Fibrillin-1 OS=Homo sapiens OX=9606 GN=FBN1 PE=1 SV=3	26	49	148	49	2871	312	4.93	404.21	49
P36955	Pigment epithelium-derived factor OS=Homo sapiens OX=9606 GN=SERPINF1 PE=1 SV=4	38	11	41	11	418	46.3	6.38	113.88	11
P37802	Transgelin-2 OS=Homo sapiens OX=9606 GN=TAGLN2 PE=1 SV=3	45	7	19	7	199	22.4	8.25	50.28	7
P40925	Malate dehydrogenase, cytoplasmic OS=Homo sapiens OX=9606 GN=MDH1 PE=1 SV=4	17	4	5	4	334	36.4	7.36	12.65	4
P41091	Eukaryotic translation initiation factor 2 subunit 3 OS=Homo sapiens OX=9606 GN=EIF2S3 PE=1 SV=3	5	2	2	2	472	51.1	8.4	4.65	2
P41222	Prostaglandin-H2 D-isomerase OS=Homo sapiens OX=9606 GN=PTGDS PE=1 SV=1	17	2	2	2	190	21	7.8	4.46	2
P43251	Biotinidase OS=Homo sapiens OX=9606 GN=BTD PE=1 SV=2	7	3	5	3	543	61.1	6.25	10.73	3
P48061	Stromal cell-derived factor 1 OS=Homo sapiens OX=9606 GN=CXCL12 PE=1 SV=1	24	2	4	2	93	10.7	9.88	11.26	2
P49721	Proteasome subunit beta type-2 OS=Homo sapiens OX=9606 GN=PSMB2 PE=1 SV=1	5	1	2	1	201	22.8	7.02	4.02	1
P49767	Vascular endothelial growth factor C OS=Homo sapiens OX=9606 GN=VEGFC PE=1 SV=1	3	1	2	1	419	46.9	7.52	5.96	1
P50454	Serpin H1 OS=Homo sapiens OX=9606 GN=SERPINH1 PE=1 SV=2	44	13	34	13	418	46.4	8.69	94.42	13
P51884	Lumican OS=Homo sapiens OX=9606 GN=LUM PE=1 SV=2	32	10	69	10	338	38.4	6.61	207.09	10

Accession	Description	Coverage [%][a]	# Peptides[b]	# PSMs[c]	# Unique Peptides[d]	# AAs[e]	MW [kDa]	Calc. pI[f]	Score Sequest HT[g]	# Peptides: Sequest HT[h]
P51991	Heterogeneous nuclear ribonucleoprotein A3 OS=Homo sapiens OX=9606 GN=HNRNPA3 PE=1 SV=2	3	1	2	1	378	39.6	9.01	3.96	1
P52943	Cysteine-rich protein 2 OS=Homo sapiens OX=9606 GN=CRIP2 PE=1 SV=1	15	1	1	1	208	22.5	8.72	2.93	1
P55058	Phospholipid transfer protein OS=Homo sapiens OX=9606 GN=PLTP PE=1 SV=1	5	2	3	2	493	54.7	7.01	6.35	2
P55072	Transitional endoplasmic reticulum ATPase OS=Homo sapiens OX=9606 GN=VCP PE=1 SV=4	11	6	9	6	806	89.3	5.26	20.58	6
P55290	Cadherin-13 OS=Homo sapiens OX=9606 GN=CDH13 PE=1 SV=1	7	4	7	4	713	78.2	4.98	17.22	4
P58546	Myotrophin OS=Homo sapiens OX=9606 GN=MTPN PE=1 SV=2	14	1	1	1	118	12.9	5.52	2.64	1
P61086	Ubiquitin-conjugating enzyme E2 K OS=Homo sapiens OX=9606 GN=UBE2K PE=1 SV=3	6	1	1	1	200	22.4	5.44	2.02	1
P62328	Thymosin beta-4 OS=Homo sapiens OX=9606 GN=TMSB4X PE=1 SV=2	27	1	1	1	44	5.1	5.06	2.13	1
P62937	Peptidyl-prolyl cis-trans isomerase A OS=Homo sapiens OX=9606 GN=PPIA PE=1 SV=2	32	3	8	3	165	18	7.81	19.19	3
P62942	Peptidyl-prolyl cis-trans isomerase FKBP1A OS=Homo sapiens OX=9606 GN=FKBP1A PE=1 SV=2	71	4	8	4	108	11.9	8.16	22.32	4
P63261	Actin, cytoplasmic 2 OS=Homo sapiens OX=9606 GN=ACTG1 PE=1 SV=1	56	13	81	1	375	41.8	5.48	246.11	13
P63313	Thymosin beta-10 OS=Homo sapiens OX=9606 GN=TMSB10 PE=1 SV=2	34	1	3	1	44	5	5.36	9.66	1
P67936	Tropomyosin alpha-4 chain OS=Homo sapiens OX=9606 GN=TPM4 PE=1 SV=3	31	8	37	2	248	28.5	4.69	93.08	8
P78539	Sushi repeat-containing protein SRPX OS=Homo sapiens OX=9606 GN=SRPX PE=1 SV=1	2	1	2	1	464	51.5	8.66	4.08	1
P80303	Nucleobindin-2 OS=Homo sapiens OX=9606 GN=NUCB2 PE=1 SV=3	8	2	4	2	420	50.2	5.12	11.32	2
Q01995	Transgelin OS=Homo sapiens OX=9606 GN=TAGLN PE=1 SV=4	44	7	46	7	201	22.6	8.84	119.56	7
Q05DH1	Proteasome subunit alpha type (Fragment) OS=Homo sapiens OX=9606 GN=PSMA7 PE=2 SV=1	5	1	2	1	238	26.7	8.87	3.98	1
Q06830	Peroxiredoxin-1 OS=Homo sapiens OX=9606 GN=PRDX1 PE=1 SV=1	48	6	13	5	199	22.1	8.13	36.61	6
Q07092	Collagen alpha-1(XVI) chain OS=Homo sapiens OX=9606 GN=COL16A1 PE=1 SV=2	4	4	7	4	1604	157.7	7.84	18.43	4
Q07954	Prolow-density lipoprotein receptor-related protein 1 OS=Homo sapiens OX=9606 GN=LRP1 PE=1 SV=2	1	5	12	5	4544	504.3	5.39	32.18	5
Q08397	Lysyl oxidase homolog 1 OS=Homo sapiens OX=9606 GN=LOXL1 PE=1 SV=2	10	4	9	4	574	63.1	7.52	22.47	4
Q08629	Testican-1 OS=Homo sapiens OX=9606 GN=SPOCK1 PE=1 SV=1	19	5	14	5	439	49.1	6.1	36.84	5
Q09666	Neuroblast differentiation-associated protein AHNAK OS=Homo sapiens OX=9606 GN=AHNAK PE=1 SV=2	11	21	37	21	5890	628.7	6.15	86.05	21
Q0QEN7	ATP synthase subunit beta (Fragment) OS=Homo sapiens OX=9606 GN=ATP5B PE=2 SV=1	4	1	1	1	445	48.1	5.07	1.94	1
Q0Z944	Beta globin (Fragment) OS=Homo sapiens OX=9606 GN=HBB PE=3 SV=1	10	1	3	1	105	11.5	6.37	6.82	1
Q10471	Polypeptide N-acetylgalactosaminyltransferase 2 OS=Homo sapiens OX=9606 GN=GALNT2 PE=1 SV=1	5	3	6	3	571	64.7	8.35	13.15	3
Q12841	Follistatin-related protein 1 OS=Homo sapiens OX=9606 GN=FSTL1 PE=1 SV=1	40	10	78	10	308	35	5.52	249.28	10
Q13219	Pappalysin-1 OS=Homo sapiens OX=9606 GN=PAPPA PE=1 SV=3	1	1	3	1	1627	180.9	6.18	6.06	1

Accession	Description	Coverage [%][a]	# Peptides[b]	# PSMs[c]	# Unique Peptides[d]	# AAs[e]	MW [kDa]	Calc. pI[f]	Score Sequest HT[g]	# Peptides: Sequest HT[h]
Q13561	Dynactin subunit 2 OS=Homo sapiens OX=9606 GN=DCTN2 PE=1 SV=4	4	1	2	1	401	44.2	5.21	4.86	1
Q13740	CD166 antigen OS=Homo sapiens OX=9606 GN=ALCAM PE=1 SV=2	5	2	3	2	583	65.1	6.25	6.83	2
Q14315	Filamin-C OS=Homo sapiens OX=9606 GN=FLNC PE=1 SV=3	5	8	15	4	2725	290.8	5.97	35.95	8
Q14393	Growth arrest-specific protein 6 OS=Homo sapiens OX=9606 GN=GAS6 PE=1 SV=3	19	9	14	9	678	74.9	5.69	37.46	9
Q14766	Latent-transforming growth factor beta-binding protein 1 OS=Homo sapiens OX=9606 GN=LTBP1 PE=1 SV=4	11	14	33	14	1721	186.7	5.96	83.41	14
Q14767	Latent-transforming growth factor beta-binding protein 2 OS=Homo sapiens OX=9606 GN=LTBP2 PE=1 SV=3	17	18	67	18	1821	194.9	5.19	194.23	18
Q15063-4	Isoform 4 of Periostin OS=Homo sapiens OX=9606 GN=POSTN	57	27	105	2	751	83.8	8.18	331.57	27
Q15063-5	Isoform 5 of Periostin OS=Homo sapiens OX=9606 GN=POSTN	57	29	116	4	809	90.4	7.39	366.34	29
Q15084	Protein disulfide-isomerase A6 OS=Homo sapiens OX=9606 GN=PDIA6 PE=1 SV=1	6	2	2	2	440	48.1	5.08	4.25	2
Q15149	Plectin OS=Homo sapiens OX=9606 GN=PLEC PE=1 SV=3	6	23	42	23	4684	531.5	5.96	96.88	23
Q15293	Reticulocalbin-1 OS=Homo sapiens OX=9606 GN=RCN1 PE=1 SV=1	13	3	7	3	331	38.9	5	19.51	3
Q15582	Transforming growth factor-beta-induced protein ig-h3 OS=Homo sapiens OX=9606 GN=TGFBI PE=1 SV=1	49	20	85	20	683	74.6	7.71	261.38	20
Q15828	Cystatin-M OS=Homo sapiens OX=9606 GN=CST6 PE=1 SV=1	14	1	2	1	149	16.5	8.09	6.77	1
Q16270	Insulin-like growth factor-binding protein 7 OS=Homo sapiens OX=9606 GN=IGFBP7 PE=1 SV=1	47	11	86	11	282	29.1	7.9	266.28	11
Q16394	Exostosin-1 OS=Homo sapiens OX=9606 GN=EXT1 PE=1 SV=2	7	4	5	4	746	86.2	9.04	11.89	4
Q16658	Fascin OS=Homo sapiens OX=9606 GN=FSCN1 PE=1 SV=3	5	2	5	2	493	54.5	7.24	17.72	2
Q16706	Alpha-mannosidase 2 OS=Homo sapiens OX=9606 GN=MAN2A1 PE=1 SV=2	1	1	1	1	1144	131.1	7.58	1.94	1
Q16777	Histone H2A type 2-C OS=Homo sapiens OX=9606 GN=HIST2H2AC PE=1 SV=4	7	1	1	1	129	14	10.9	2.09	1
Q16778	Histone H2B type 2-E OS=Homo sapiens OX=9606 GN=HIST2H2BE PE=1 SV=3	12	1	3	1	126	13.9	10.32	8.55	1
Q2HIZ0	Thrombospondin 3 OS=Homo sapiens OX=9606 GN=THBS3 PE=2 SV=1	7	4	10	4	956	104.1	4.65	26.36	4
Q3B7B1	PENK protein (Fragment) OS=Homo sapiens OX=9606 GN=PENK PE=2 SV=1	29	3	6	3	189	21.5	6.77	15.63	3
Q4ZHG4	Fibronectin type III domain-containing protein 1 OS=Homo sapiens OX=9606 GN=FNDC1 PE=2 SV=4	7	9	13	9	1894	205.4	9.32	30.5	9
Q53FA4	Cysteine-rich, angiogenic inducer, 61 variant (Fragment) OS=Homo sapiens OX=9606 PE=2 SV=1	18	4	6	4	381	42	8.31	16.93	4
Q53G99	Beta actin variant (Fragment) OS=Homo sapiens OX=9606 PE=2 SV=1	56	13	83	1	375	41.7	5.59	254.31	13
Q53GN4	WD repeat domain 1, isoform CRA_a (Fragment) OS=Homo sapiens OX=9606 GN=WDR1 PE=2 SV=1	10	3	3	3	606	66.1	6.65	7.65	3
Q53GY0	Plastin 3 variant (Fragment) OS=Homo sapiens OX=9606 PE=2 SV=1	24	11	19	11	630	70.7	5.68	44.04	11
Q53HQ7	Elongation factor 1-alpha (Fragment) OS=Homo sapiens OX=9606 PE=2 SV=1	16	4	10	4	462	50.2	8.94	28.23	4
Q53R94	Uncharacterized protein RTN4 (Fragment) OS=Homo sapiens OX=9606 GN=RTN4 PE=4 SV=1	7	1	1	1	185	19.3	4.13	1.97	1

Accession	Description	Coverage [%][a]	# Peptides[b]	# PSMs[c]	# Unique Peptides[d]	# AAs[e]	MW [kDa]	Calc. pI[f]	Score Sequest HT[g]	# Peptides: Sequest HT[h]
Q53XB4	Full-length cDNA clone CS0DF032YM23 of Fetal brain of Homo sapiens (human) OS=Homo sapiens OX=9606 GN=RAB1 PE=2 SV=1	21	2	4	2	147	16.8	9.03	11.82	2
Q59GA0	Thy-1 cell surface antigen variant (Fragment) OS=Homo sapiens OX=9606 PE=2 SV=1	20	2	5	2	145	15.9	9	13.4	2
Q5JRP2	Disintegrin and metalloproteinase domain-containing protein 12 (Fragment) OS=Homo sapiens OX=9606 GN=ADAM12 PE=1 SV=1	6	1	1	1	278	31.1	9.11	2.13	1
Q5M8T4	Connective tissue growth factor OS=Homo sapiens OX=9606 GN=CTGF PE=2 SV=1	44	11	26	11	349	38	7.94	63.85	11
Q5T7F0	Neuropilin OS=Homo sapiens OX=9606 GN=NRP1 PE=1 SV=1	5	2	3	2	704	79	5.86	6.95	2
Q5TZP7	DNA-(apurinic or apyrimidinic site) lyase OS=Homo sapiens OX=9606 GN=APEX1 PE=2 SV=1	7	2	2	2	318	35.5	8.12	3.97	2
Q5U000	Cathepsin Z OS=Homo sapiens OX=9606 PE=2 SV=1	3	1	1	1	303	33.8	7.11	1.99	1
Q5UGI6	Serine/cysteine proteinase inhibitor clade G member 1 splice variant 2 (Fragment) OS=Homo sapiens OX=9606 GN=SERPING1 PE=2 SV=1	20	6	19	6	333	37.3	8	54.38	6
Q63HR1	Uncharacterized protein DKFZp686P17171 OS=Homo sapiens OX=9606 GN=DKFZp686P17171 PE=2 SV=1	4	1	1	1	387	42.4	8.44	2.6	1
Q6DEN2	DPYSL3 protein OS=Homo sapiens OX=9606 GN=DPYSL3 PE=2 SV=1	4	2	2	2	684	73.9	6.55	4.87	2
Q6EMK4	Vasorin OS=Homo sapiens OX=9606 GN=VASN PE=1 SV=1	14	6	16	6	673	71.7	7.39	42.33	6
Q6FGD7	Tubulin-specific chaperone A OS=Homo sapiens OX=9606 GN=TBCA PE=2 SV=1	10	1	3	1	108	12.8	5.29	8.24	1
Q6FH49	NNMT protein OS=Homo sapiens OX=9606 GN=NNMT PE=2 SV=1	3	1	1	1	264	29.6	5.74	1.97	1
Q6FHC9	STC2 protein (Fragment) OS=Homo sapiens OX=9606 GN=STC2 PE=2 SV=1	24	3	18	3	302	33.2	7.3	65.11	3
Q6FHE1	FST protein OS=Homo sapiens OX=9606 GN=FST PE=2 SV=1	9	1	3	1	317	34.8	7.65	10.48	1
Q6FHU2	Phosphoglycerate mutase (Fragment) OS=Homo sapiens OX=9606 GN=PGAM1 PE=2 SV=1	26	3	3	3	254	28.8	7.18	7.63	3
Q6FHU3	PSME1 protein (Fragment) OS=Homo sapiens OX=9606 GN=PSME1 PE=2 SV=1	3	1	1	1	249	28.7	6.02	1.94	1
Q6FHW3	DF protein OS=Homo sapiens OX=9606 GN=DF PE=2 SV=1	18	2	3	2	228	24.4	7.24	7.61	2
Q6FHZ0	Malate dehydrogenase OS=Homo sapiens OX=9606 GN=MDH2 PE=2 SV=1	15	3	5	3	338	35.5	8.68	13.03	3
Q6IAW5	CALU protein OS=Homo sapiens OX=9606 GN=CALU PE=2 SV=1	38	10	28	10	315	37.1	4.64	75.17	10
Q6NUL1	Exostoses (Multiple) 2 OS=Homo sapiens OX=9606 GN=EXT2 PE=2 SV=1	11	5	10	5	718	82.1	6.55	26.37	5
Q6NUR7	Ezrin OS=Homo sapiens OX=9606 GN=EZR PE=2 SV=1	9	6	10	3	586	69.2	6.27	22.1	6
Q6NVI1	MARCKS protein (Fragment) OS=Homo sapiens OX=9606 GN=MARCKS PE=2 SV=1	20	1	3	1	157	14.9	4.79	10.25	1
Q6UVK1	Chondroitin sulfate proteoglycan 4 OS=Homo sapiens OX=9606 GN=CSPG4 PE=1 SV=2	3	4	7	4	2322	250.4	5.47	17.44	4
Q6UWH4	Protein FAM198B OS=Homo sapiens OX=9606 GN=FAM198B PE=2 SV=1	3	1	2	1	519	57.5	9.7	4.58	1
Q6YHK3	CD109 antigen OS=Homo sapiens OX=9606 GN=CD109 PE=1 SV=2	8	7	12	7	1445	161.6	5.85	29.22	7
Q76LA1	CSTB protein OS=Homo sapiens OX=9606 GN=CSTB PE=2 SV=1	12	1	2	1	98	11.1	7.56	4.05	1
Q7Z2W2	Extracellular sulfatase OS=Homo sapiens OX=9606 GN=DKFZp686F13142 PE=2 SV=1	3	2	4	2	871	101	9.09	10.29	2

Accession	Description	Coverage [%][a]	# Peptides[b]	# PSMs[c]	# Unique Peptides[d]	# AAs[e]	MW [kDa]	Calc. pI[f]	Score Sequest HT[g]	# Peptides: Sequest HT[h]
Q7Z7M9	Polypeptide N-acetylgalactosaminyltransferase 5 OS=Homo sapiens OX=9606 GN=GALNT5 PE=1 SV=1	3	2	6	2	940	106.2	9.47	16.76	2
Q86SR1	Polypeptide N-acetylgalactosaminyltransferase 10 OS=Homo sapiens OX=9606 GN=GALNT10 PE=1 SV=2	2	1	1	1	603	68.9	8.59	2.47	1
Q86X91	Inactive tyrosine-protein kinase 7 OS=Homo sapiens OX=9606 GN=PTK7 PE=1 SV=1	8	3	5	3	459	50.4	6.65	11.57	3
Q86Z22	Epididymis secretory protein Li 297 OS=Homo sapiens OX=9606 GN=HEL-S-297 PE=2 SV=1	8	2	4	2	226	23.8	9.06	12.06	2
Q8IUX7	Adipocyte enhancer-binding protein 1 OS=Homo sapiens OX=9606 GN=AEBP1 PE=1 SV=1	17	13	43	13	1158	130.8	5.11	122.52	13
Q8N7G1	Purine nucleoside phosphorylase OS=Homo sapiens OX=9606 PE=2 SV=1	12	2	5	2	293	32.5	7.21	12.47	2
Q8NBJ4	Golgi membrane protein 1 OS=Homo sapiens OX=9606 GN=GOLM1 PE=1 SV=1	3	1	2	1	401	45.3	4.97	5.21	1
Q8WUJ3	Cell migration-inducing and hyaluronan-binding protein OS=Homo sapiens OX=9606 GN=CEMIP PE=1 SV=2	3	3	5	3	1361	152.9	7.85	10.95	3
Q92520	Protein FAM3C OS=Homo sapiens OX=9606 GN=FAM3C PE=1 SV=1	7	1	3	1	227	24.7	8.29	7.55	1
Q92626	Peroxidasin homolog OS=Homo sapiens OX=9606 GN=PXDN PE=1 SV=2	31	27	45	27	1479	165.2	7.17	126.98	27
Q969H8	Myeloid-derived growth factor OS=Homo sapiens OX=9606 GN=MYDGF PE=1 SV=1	9	1	4	1	173	18.8	6.68	10	1
Q96AY3	Peptidyl-prolyl cis-trans isomerase FKBP10 OS=Homo sapiens OX=9606 GN=FKBP10 PE=1 SV=1	25	11	27	11	582	64.2	5.62	69.5	11
Q96CE4	Stathmin OS=Homo sapiens OX=9606 GN=STMN1 PE=1 SV=1	9	1	3	1	149	17.3	5.97	7.87	1
Q96HC4	PDZ and LIM domain protein 5 OS=Homo sapiens OX=9606 GN=PDLIM5 PE=1 SV=5	10	4	5	4	596	63.9	8.21	12.52	4
Q99715	Collagen alpha-1(XII) chain OS=Homo sapiens OX=9606 GN=COL12A1 PE=1 SV=2	36	70	240	70	3063	332.9	5.53	670.93	70
Q99985	Semaphorin-3C OS=Homo sapiens OX=9606 GN=SEMA3C PE=2 SV=2	6	3	3	3	751	85.2	8.69	6.97	3
Q9BRK3	Matrix remodeling-associated protein 8 OS=Homo sapiens OX=9606 GN=MXRA8 PE=1 SV=1	7	3	7	3	442	49.1	7.23	15.62	3
Q9BS26	Endoplasmic reticulum resident protein 44 OS=Homo sapiens OX=9606 GN=ERP44 PE=1 SV=1	6	1	1	1	406	46.9	5.26	2.68	1
Q9NS15	Latent-transforming growth factor beta-binding protein 3 OS=Homo sapiens OX=9606 GN=LTBP3 PE=1 SV=4	2	2	2	2	1303	139.3	6.07	4.68	2
Q9P1F3	Costars family protein ABRACL OS=Homo sapiens OX=9606 GN=ABRACL PE=1 SV=1	20	1	1	1	81	9.1	6.29	2.63	1
Q9UKP4	A disintegrin and metalloproteinase with thrombospondin motifs 7 OS=Homo sapiens OX=9606 GN=ADAMTS7 PE=1 SV=2	2	3	5	3	1686	184	6.2	12.29	3
Q9UNN8	Endothelial protein C receptor OS=Homo sapiens OX=9606 GN=PROCR PE=1 SV=1	7	1	3	1	238	26.7	7.18	9.2	1
Q9Y240	C-type lectin domain family 11 member A OS=Homo sapiens OX=9606 GN=CLEC11A PE=1 SV=1	12	3	5	3	323	35.7	5.16	13.43	3
Q9Y490	Talin-1 OS=Homo sapiens OX=9606 GN=TLN1 PE=1 SV=3	12	19	32	19	2541	269.6	6.07	74.66	19
Q9Y4K0	Lysyl oxidase homolog 2 OS=Homo sapiens OX=9606 GN=LOXL2 PE=1 SV=1	11	4	8	4	774	86.7	6.38	23.39	4
Q9Y646	Carboxypeptidase Q OS=Homo sapiens OX=9606 GN=CPQ PE=1 SV=1	7	3	8	3	472	51.9	6.18	17.04	3
Q9Y6C2	EMILIN-1 OS=Homo sapiens OX=9606 GN=EMILIN1 PE=1 SV=3	19	11	16	11	1016	106.6	5.17	42.25	11
S4R3N1	HSPE1-MOB4 readthrough OS=Homo sapiens OX=9606 GN=HSPE1-MOB4 PE=3 SV=1	5	1	3	1	261	29.7	6.16	8.55	1

Accession	Description	Coverage [%][a]	# Peptides[b]	# PSMs[c]	# Unique Peptides[d]	# AAs[e]	MW [kDa]	Calc. pI[f]	Score Sequest HT[g]	# Peptides: Sequest HT[h]
V9H1C1	Gelsolin exon 4 (Fragment) OS=Homo sapiens OX=9606 PE=4 SV=1	35	2	4	1	69	8	5.78	11.76	2
V9HW22	Epididymis luminal protein 33 OS=Homo sapiens OX=9606 GN=HEL-S-72p PE=2 SV=1	20	11	30	9	646	70.9	5.52	77.46	11
V9HW48	SH3 domain-binding glutamic acid-rich-like protein OS=Homo sapiens OX=9606 GN=HEL-S-115 PE=2 SV=1	11	1	1	1	114	12.8	5.25	1.93	1
V9HW74	Ubiquitin carboxyl-terminal hydrolase OS=Homo sapiens OX=9606 GN=HEL-117 PE=2 SV=1	8	1	3	1	223	24.8	5.48	8.4	1
V9HW92	Epididymis secretory protein Li 112 OS=Homo sapiens OX=9606 GN=HEL-S-112 PE=2 SV=1	4	1	1	1	329	36	7.02	2.12	1
V9HW98	Epididymis luminal protein 2 OS=Homo sapiens OX=9606 GN=HEL2 PE=1 SV=1	35	7	14	6	255	29.2	4.74	35.53	7
V9HWB4	Epididymis secretory sperm binding protein Li 89n OS=Homo sapiens OX=9606 GN=HEL-S-89n PE=2 SV=1	32	15	39	14	654	72.3	5.16	101.7	15
V9HWB8	Pyruvate kinase OS=Homo sapiens OX=9606 GN=HEL-S-30 PE=1 SV=1	44	18	51	18	531	57.9	7.84	136.18	18
V9HWC6	Peptidyl-prolyl cis-trans isomerase OS=Homo sapiens OX=9606 GN=HEL-S-39 PE=2 SV=1	35	6	14	6	208	22.7	9.32	36.64	6
V9HWE8	Epididymis secretory sperm binding protein Li 47e OS=Homo sapiens OX=9606 GN=HEL-S-47e PE=2 SV=1	41	5	12	5	204	23.2	5.11	32.21	5
V9HWE9	Epididymis secretory protein Li 22 OS=Homo sapiens OX=9606 GN=HEL-S-22 PE=2 SV=1	37	5	8	3	210	23.3	5.64	18.86	5
V9HWF4	Phosphoglycerate kinase OS=Homo sapiens OX=9606 GN=HEL-S-68p PE=2 SV=1	19	5	9	5	417	44.6	8.1	24.63	5
V9HWI5	Cofilin 1 (Non-muscle), isoform CRA_b OS=Homo sapiens OX=9606 GN=HEL-S-15 PE=2 SV=1	57	7	14	7	166	18.5	8.09	35.57	7
V9HWK1	Triosephosphate isomerase OS=Homo sapiens OX=9606 GN=HEL-S-49 PE=2 SV=1	50	10	30	10	249	26.7	6.9	89.05	10
V9HWN7	Fructose-bisphosphate aldolase OS=Homo sapiens OX=9606 GN=HEL-S-87p PE=2 SV=1	38	8	20	7	364	39.4	8.09	52.69	8
V9HWP2	Epididymis luminal protein 35 OS=Homo sapiens OX=9606 GN=HEL-S-125m PE=2 SV=1	6	4	12	3	803	92.4	4.84	32.19	4

[a] Coverage is the percentage of the sequence covered by identifications (PSMs only) from the included searches. [b] # Peptides is the number of distinct peptide sequences in the protein. [c] # PSMs is number of identified peptide spectrum matches identified from all included searches, including those redundantly identified. [d] # Unique peptide is the total number of distinct peptide sequences unique to the protein group. [e] #AAs is length of the protein sequence. [f] Calc pI (isoelectric point) number is theoretically calculated isoelectric point for the protein that is, the pH at which a particular molecule carries no net electrical charge. [g] Score Sequest HT is a protein score calculated by summing the individual scores for each peptide. The higher this score, the higher the individual score of the peptide, allowing for better identification. [h] # Peptides: Sequest HT is the number of distinct peptide sequences in the identified protein.

Supplementary Table 1-2: Detailed information on commonly secreted proteins by 3 bone marrow (BM) mesenchymal stem cells (MSCs) under inflammatory condition.

Accession	Description	Coverage [%][a]	# Peptides[b]	# PSMs[c]	# Unique Peptides[d]	# AAs[e]	MW [kDa]	Calc. pI[f]	Score Sequest HT[g]	# Peptides: Sequest HT[h]
A0A024QYX3	RNA binding motif (RNP1, RRM) protein 3, isoform CRA_c OS=Homo sapiens OX=9606 GN=RBM3 PE=4 SV=1	11	1	2	1	157	17.2	8.91	5.46	1
A0A024QZL1	Proteoglycan 1, secretory granule, isoform CRA_a OS=Homo sapiens OX=9606 GN=PRG1 PE=4 SV=1	17	2	7	2	158	17.6	4.86	18.9	2
A0A024R0A1	Macrophage colony-stimulating factor 1 OS=Homo sapiens OX=9606 GN=CSF1 PE=4 SV=1	11	4	9	4	554	60.1	5.29	23.73	4
A0A024R0P8	Transforming growth factor, beta 1 (Camurati-Engelmann disease), isoform CRA_a OS=Homo sapiens OX=9606 GN=TGFB1 PE=3 SV=1	3	1	3	1	390	44.3	8.53	6.09	1
A0A024R1N1	Myosin, heavy polypeptide 9, non-muscle, isoform CRA_a OS=Homo sapiens OX=9606 GN=MYH9 PE=3 SV=1	9	13	23	13	1960	226.4	5.6	54.95	13
A0A024R1T9	ATP-citrate synthase OS=Homo sapiens OX=9606 GN=ACLY PE=3 SV=1	4	3	3	3	1101	120.8	7.33	6.61	3
A0A024R1U8	Insulin-like growth factor binding protein 4, isoform CRA_a OS=Homo sapiens OX=9606 GN=IGFBP4 PE=4 SV=1	25	5	38	5	258	27.9	7.15	117.93	5
A0A024R222	Phosphoserine aminotransferase OS=Homo sapiens OX=9606 GN=PSAT1 PE=3 SV=1	9	3	4	3	370	40.4	7.66	10.2	3
A0A024R2P0	40S ribosomal protein SA OS=Homo sapiens OX=9606 GN=RPSA PE=3 SV=1	9	2	4	2	295	32.8	4.87	9.77	2
A0A024R2W4	Dystroglycan 1 (Dystrophin-associated glycoprotein 1), isoform CRA_a OS=Homo sapiens OX=9606 GN=DAG1 PE=4 SV=1	6	3	8	3	895	97.5	8.56	23.29	3
A0A024R433	Insulin-like growth factor binding protein 5, isoform CRA_a OS=Homo sapiens OX=9606 GN=IGFBP5 PE=4 SV=1	25	5	14	5	272	30.6	8.21	35.34	5
A0A024R462	Fibronectin 1, isoform CRA_n OS=Homo sapiens OX=9606 GN=FN1 PE=4 SV=1	52	69	924	39	2355	259	5.73	2884.47	69
A0A024R509	Arylsulfatase A, isoform CRA_a OS=Homo sapiens OX=9606 GN=ARSA PE=4 SV=1	3	1	1	1	509	53.8	6.07	2.07	1
A0A024R5Z7	Annexin OS=Homo sapiens OX=9606 GN=ANXA2 PE=3 SV=1	42	13	52	13	339	38.6	7.75	140.59	13
A0A024R6K8	Tryptophanyl-tRNA synthetase, isoform CRA_a OS=Homo sapiens OX=9606 GN=WARS PE=3 SV=1	7	2	3	2	471	53.1	6.23	6.82	2
A0A024R6R4	Matrix metallopeptidase 2 (Gelatinase A, 72kDa gelatinase, 72kDa type IV collagenase), isoform CRA_a OS=Homo sapiens OX=9606 GN=MMP2 PE=3 SV=1	58	25	194	25	660	73.8	5.47	593.2	25
A0A024R8E5	Collagen, type V, alpha 1, isoform CRA_a OS=Homo sapiens OX=9606 GN=COL5A1 PE=4 SV=1	12	12	38	12	1838	183.4	5.06	105.82	12
A0A024R8S5	Protein disulfide-isomerase OS=Homo sapiens OX=9606 GN=P4HB PE=2 SV=1	25	8	16	8	508	57.1	4.87	44.7	8
A0A024R8V7	TIMP metallopeptidase inhibitor 2, isoform CRA_b OS=Homo sapiens OX=9606 GN=TIMP2 PE=4 SV=1	32	4	22	4	179	20.2	7.17	65.85	4
A0A024R969	Chitinase 3-like 1 (Cartilage glycoprotein-39), isoform CRA_a OS=Homo sapiens OX=9606 GN=CHI3L1 PE=3 SV=1	49	13	70	13	383	42.6	8.46	197.22	13
A0A024RA94	Microfibrillar-associated protein 2, isoform CRA_d OS=Homo sapiens OX=9606 GN=MFAP2 PE=4 SV=1	17	2	3	2	183	20.8	4.97	6.96	2
A0A024RAI1	ARP3 actin-related protein 3 homolog (Yeast), isoform CRA_a OS=Homo sapiens OX=9606 GN=ACTR3 PE=3 SV=1	14	4	7	4	418	47.3	5.88	16.8	4
A0A024RAK9	Hyaluronan and proteoglycan link protein 1, isoform CRA_a OS=Homo sapiens OX=9606 GN=HAPLN1 PE=4 SV=1	9	2	6	2	354	40.1	7.42	20.18	2
A0A024RAM4	Microtubule-associated protein 1B, isoform CRA_b OS=Homo sapiens OX=9606 GN=MAP1B PE=4 SV=1	1	1	1	1	2468	270.5	4.81	2.28	1
A0A024RAQ9	Chondroitin sulfate proteoglycan 2 (Versican), isoform CRA_b OS=Homo sapiens OX=9606 GN=CSPG2 PE=4 SV=1	5	12	47	12	3396	372.6	4.51	128.69	12
A0A024RC55	Milk fat globule-EGF factor 8 protein, isoform CRA_a OS=Homo sapiens OX=9606 GN=MFGE8 PE=4 SV=1	40	10	27	1	387	43.1	8.15	79.01	10
A0A024RD80	Heat shock protein 90kDa alpha (Cytosolic), class B member 1, isoform CRA_a OS=Homo sapiens OX=9606 GN=HSP90AB1 PE=3 SV=1	16	8	15	7	724	83.2	5.03	35.18	8

Accession	Description	Coverage [%][a]	# Peptides[b]	# PSMs[c]	# Unique Peptides[d]	# AAs[e]	MW [kDa]	Calc. pI[f]	Score Sequest HT[g]	# Peptides: Sequest HT[h]
A0A024RDA4	C-X-C motif chemokine OS=Homo sapiens OX=9606 GN=CXCL10 PE=3 SV=1	27	2	4	2	98	10.9	9.86	10	2
A0A024RDA5	Multifunctional fusion protein OS=Homo sapiens OX=9606 GN=IL8 PE=3 SV=1	16	1	11	1	99	11.1	8.84	30.71	1
A0A024RDW8	Collagen, type IV, alpha 2, isoform CRA_a OS=Homo sapiens OX=9606 GN=COL4A2 PE=4 SV=1	29	28	85	28	1712	167.4	8.66	228.2	28
A0A087WTA8	Collagen alpha-2(I) chain OS=Homo sapiens OX=9606 GN=COL1A2 PE=1 SV=1	60	43	809	43	1364	129.1	9.01	2579.52	43
A0A087WVN4	Farnesyl pyrophosphate synthase (Fragment) OS=Homo sapiens OX=9606 GN=FDPS PE=1 SV=1	7	1	1	1	274	31.8	7.28	2.31	1
A0A087WXI7	Integrin beta-like protein 1 OS=Homo sapiens OX=9606 GN=ITGBL1 PE=1 SV=1	17	5	8	5	401	43.7	5.27	20.19	5
A0A087WZ51	Tumor protein D54 OS=Homo sapiens OX=9606 GN=TPD52L2 PE=1 SV=1	13	1	2	1	152	16.3	4.92	4.6	1
A0A087X0S5	Collagen alpha-1(VI) chain OS=Homo sapiens OX=9606 GN=COL6A1 PE=1 SV=1	44	26	179	26	1026	108.3	5.43	541.22	26
A0A090N8G0	Glycyl-tRNA synthetase OS=Homo sapiens OX=9606 GN=GARS PE=4 SV=1	2	1	3	1	685	77.5	6.24	9.34	1
A0A090N8Y2	Protein disulfide-isomerase A4 OS=Homo sapiens OX=9606 GN=ERP70 PE=2 SV=1	4	2	4	2	645	72.9	5.07	10.24	2
A0A0A0MT01	Gelsolin OS=Homo sapiens OX=9606 GN=GSN PE=1 SV=1	20	9	21	9	767	84.7	5.83	54.74	9
A0A0A0MTS2	Glucose-6-phosphate isomerase (Fragment) OS=Homo sapiens OX=9606 GN=GPI PE=1 SV=1	8	3	3	3	573	64.8	9.04	6.54	3
A0A0D9SF54	Spectrin alpha chain, non-erythrocytic 1 OS=Homo sapiens OX=9606 GN=SPTAN1 PE=1 SV=1	3	4	6	4	2457	282.7	5.34	13.65	4
A0A0H3W617	Stanniocalcin 1 OS=Homo sapiens OX=9606 GN=stc1 PE=2 SV=1	8	1	3	1	178	20.5	8.72	7.49	1
A0A0S2Z3G9	Actinin alpha 4 isoform 1 (Fragment) OS=Homo sapiens OX=9606 GN=ACTN4 PE=2 SV=1	38	26	67	16	911	104.8	5.44	169	26
A0A0S2Z3Y1	Lectin galactoside-binding soluble 3 binding protein isoform 1 (Fragment) OS=Homo sapiens OX=9606 GN=LGALS3BP PE=2 SV=1	18	6	17	6	585	65.3	5.27	44.32	6
A0A0U1RRM8	Fermitin family homolog 2 (Fragment) OS=Homo sapiens OX=9606 GN=FERMT2 PE=1 SV=1	3	1	1	1	545	61.9	5.81	2.32	1
A0A140VJI7	Testicular tissue protein Li 61 OS=Homo sapiens OX=9606 PE=2 SV=1	26	9	16	9	540	60.6	6.71	40.26	9
A0A140VJZ1	Ubiquitin carboxyl-terminal hydrolase OS=Homo sapiens OX=9606 PE=2 SV=1	2	1	1	1	858	95.7	5.03	2.08	1
A0A146E5L3	Mesenchymal stromal cell-and fibroblast-expressing Linx paralogue OS=Homo sapiens OX=9606 GN=Meflin PE=2 SV=1	15	5	20	5	428	46	5.15	53.75	5
A0A161I202	Lactoferrin OS=Homo sapiens OX=9606 GN=LTF PE=2 SV=1	2	1	1	1	711	78.3	8.18	2.06	1
A0A172Q3A0	Fibroblast activation protein (Fragment) OS=Homo sapiens OX=9606 PE=2 SV=1	2	1	1	1	733	84.8	6.55	2.03	1
A0A1B0GTB0	Renin receptor (Fragment) OS=Homo sapiens OX=9606 GN=ATP6AP2 PE=1 SV=1	6	1	1	1	294	33	6.07	1.99	1
A0A1B0GU92	Uncharacterized protein OS=Homo sapiens OX=9606 PE=1 SV=1	11	4	8	4	557	59.8	6.62	19.03	4
A0A1U9X7H4	CFB OS=Homo sapiens OX=9606 PE=3 SV=1	20	10	27	10	764	85.5	6.96	74.55	10
A0A2U3TZL5	CD59 glycoprotein (Fragment) OS=Homo sapiens OX=9606 GN=CD59 PE=4 SV=1	10	1	1	1	120	13.3	6.25	3.05	1
A0A2Z6ATB6	Drebrin A OS=Homo sapiens OX=9606 GN=DBN1 PE=2 SV=1	5	2	4	2	695	76.3	4.51	9.68	2
A1KY36	Cell proliferation-inducing protein 41 OS=Homo sapiens OX=9606 PE=2 SV=1	21	8	21	8	745	73.4	9.61	61.23	8

Accession	Description	Coverage [%][a]	# Peptides[b]	# PSMs[c]	# Unique Peptides[d]	# AAs[e]	MW [kDa]	Calc. pI[f]	Score Sequest HT[g]	# Peptides: Sequest HT[h]
A1L4H1	Soluble scavenger receptor cysteine-rich domain-containing protein SSC5D OS=Homo sapiens OX=9606 GN=SSC5D PE=1 SV=3	2	1	2	1	1573	165.6	6.13	4.81	1
A4D1W7	Inhibin, beta A (Activin A, activin AB alpha polypeptide) OS=Homo sapiens OX=9606 GN=INHBA PE=2 SV=1	33	12	67	12	426	47.4	8.03	203.71	12
A4D275	Actin-related protein 2/3 complex subunit OS=Homo sapiens OX=9606 GN=ARPC1B PE=2 SV=1	6	2	4	2	372	40.9	8.35	9.12	2
A4D2D2	Procollagen C-endopeptidase enhancer OS=Homo sapiens OX=9606 GN=PCOLCE PE=4 SV=1	25	7	18	7	449	47.9	7.43	50.29	7
A6XMH5	Beta-2-microglobulin OS=Homo sapiens OX=9606 PE=2 SV=1	24	1	8	1	92	10.4	8.02	25.22	1
A6XND1	Insulin-like growth factor binding protein 3 isoform b OS=Homo sapiens OX=9606 GN=IGFBP3 PE=1 SV=1	30	5	20	5	263	29	8.46	55.2	5
A8K2Q6	Peptidyl-prolyl cis-trans isomerase OS=Homo sapiens OX=9606 PE=2 SV=1	6	1	2	1	212	22.7	8.4	4.12	1
A8K7Q1	cDNA FLJ77770, highly similar to Homo sapiens nucleobindin 1 (NUCB1), mRNA OS=Homo sapiens OX=9606 PE=2 SV=1	33	12	39	12	461	53.9	5.25	106.85	12
A8KA16	cDNA FLJ77243, highly similar to Homo sapiens steroid sensitive gene 1 (URB), transcript variant 1, mRNA OS=Homo sapiens OX=9606 PE=2 SV=1	17	11	29	11	950	108.1	9.69	71	11
A8KAJ3	cDNA FLJ77823, highly similar to Homo sapiens EGF-containing fibulin-like extracellular matrix protein 1, transcript variant 3, mRNA OS=Homo sapiens OX=9606 PE=2 SV=1	41	14	30	14	493	54.6	5.14	80.77	14
A8MU27	Small ubiquitin-related modifier 3 OS=Homo sapiens OX=9606 GN=SUMO3 PE=1 SV=1	8	1	1	1	147	16.9	9.67	2.05	1
B2R4R0	Histone H4 OS=Homo sapiens OX=9606 GN=HIST1H4H PE=2 SV=1	21	2	5	2	103	11.4	11.36	13.25	2
B2R582	cDNA, FLJ92374, highly similar to Homo sapiens C-type lectin domain family 3, member B (CLEC3B), mRNA OS=Homo sapiens OX=9606 PE=2 SV=1	22	3	8	3	202	22.5	6.04	16.67	3
B2R5J8	C-C motif chemokine OS=Homo sapiens OX=9606 PE=2 SV=1	12	1	1	1	91	10	9.07	2.23	1
B2R5M9	cDNA, FLJ92537, highly similar to Homo sapiens procollagen-lysine, 2-oxoglutarate 5-dioxygenase (lysine hydroxylase, Ehlers-Danlos syndrome type VI) (PLOD), mRNA OS=Homo sapiens OX=9606 PE=2 SV=1	12	7	9	7	727	83.5	7.02	20.29	7
B2R642	cDNA, FLJ92775, highly similar to Homo sapiens melanoma cell adhesion molecule (MCAM), mRNA OS=Homo sapiens OX=9606 PE=2 SV=1	2	1	3	1	646	71.6	5.76	6.45	1
B2R7T8	cDNA, FLJ93598, highly similar to Homo sapiens capping protein (actin filament) muscle Z-line, beta (CAPZB), mRNA OS=Homo sapiens OX=9606 PE=2 SV=1	18	4	6	4	272	30.6	6	13.8	4
B2R960	cDNA, FLJ94230, highly similar to Homo sapiens thioredoxin-like 1 (TXNL1), mRNA OS=Homo sapiens OX=9606 PE=2 SV=1	5	1	1	1	289	32.2	4.96	2.01	1
B2RBS8	cDNA, FLJ95666, highly similar to Homo sapiens albumin (ALB), mRNA OS=Homo sapiens OX=9606 PE=2 SV=1	2	2	9	2	609	69.3	6.28	21.9	2
B2RCM5	cDNA, FLJ96160, highly similar to Homo sapiens EGF-containing fibulin-like extracellular matrix protein 2 (EFEMP2), mRNA OS=Homo sapiens OX=9606 PE=2 SV=1	15	5	11	5	443	49.3	4.94	25.69	5
B2RDW0	cDNA, FLJ96792, highly similar to Homo sapiens calmodulin 2 (phosphorylase kinase, delta) (CALM2), mRNA OS=Homo sapiens OX=9606 PE=2 SV=1	21	2	4	2	149	16.8	4.22	9.23	2
B3KQT9	Protein disulfide-isomerase OS=Homo sapiens OX=9606 PE=2 SV=1	23	8	14	7	480	54.1	7.21	31.05	8
B3KY04	cDNA FLJ46506 fis, clone THYMU3030752, highly similar to BTB/POZ domain-containing protein KCTD12 OS=Homo sapiens OX=9606 PE=2 SV=1	21	5	9	5	325	35.7	5.64	19.45	5
B4DDQ2	Biglycan OS=Homo sapiens OX=9606 PE=2 SV=1	51	12	101	12	334	37.6	7.97	321.3	12
B4DE78	cDNA FLJ52141, highly similar to 14-3-3 protein gamma OS=Homo sapiens OX=9606 PE=2 SV=1	21	3	6	2	207	23.5	4.82	17.74	3
B4DF70	cDNA FLJ60461, highly similar to Peroxiredoxin-2 OS=Homo sapiens OX=9606 PE=2 SV=1	16	2	4	1	183	20.1	8.78	9.6	2
B4DFR2	cDNA FLJ59194, moderately similar to Dynein light chain 2A, cytoplasmic OS=Homo sapiens OX=9606 PE=2 SV=1	8	1	1	1	121	13.4	10.08	1.93	1

Accession	Description	Coverage [%][a]	# Peptides[b]	# PSMs[c]	# Unique Peptides[d]	# AAs[e]	MW [kDa]	Calc. pI[f]	Score Sequest HT[g]	# Peptides: Sequest HT[h]
B4DHK9	cDNA FLJ51656, highly similar to Actin-like protein 2 OS=Homo sapiens OX=9606 PE=2 SV=1	17	4	7	4	342	39	5.91	16.58	4
B4DJ30	cDNA FLJ61290, highly similar to Neutral alpha-glucosidase AB OS=Homo sapiens OX=9606 PE=2 SV=1	6	4	6	4	995	112.9	6.06	14.26	4
B4DJI4	cDNA FLJ58065, highly similar to LIM and SH3 domain protein 1 OS=Homo sapiens OX=9606 PE=2 SV=1	12	2	2	2	220	24.5	8.54	4.29	2
B4DJQ5	cDNA FLJ59211, highly similar to Glucosidase 2 subunit beta OS=Homo sapiens OX=9606 PE=2 SV=1	10	4	6	4	535	60.1	4.42	14.17	4
B4DKJ4	cDNA FLJ57738, highly similar to Translationally-controlled tumor protein OS=Homo sapiens OX=9606 PE=2 SV=1	10	1	2	1	142	16.1	4.68	6.03	1
B4DKZ9	cDNA FLJ55705, highly similar to Threonyl-tRNA synthetase, cytoplasmic OS=Homo sapiens OX=9606 PE=2 SV=1	2	1	1	1	602	70.3	8.56	2.09	1
B4DLV7	Rab GDP dissociation inhibitor OS=Homo sapiens OX=9606 PE=2 SV=1	27	8	10	5	449	51.1	8.18	23.82	8
B4DM79	cDNA FLJ53848, highly similar to Inter-alpha-trypsin inhibitor heavy chain H2 OS=Homo sapiens OX=9606 PE=2 SV=1	5	2	4	2	543	60.7	5.35	9.34	2
B4DMR3	cDNA FLJ51896, highly similar to Glia-derived nexin OS=Homo sapiens OX=9606 PE=2 SV=1	49	14	104	14	334	37.1	9.52	273.65	14
B4DN59	cDNA FLJ52702, highly similar to Homo sapiens CD44 antigen (homing function and Indian blood group system) (CD44), transcript variant 4, mRNA OS=Homo sapiens OX=9606 PE=2 SV=1	12	3	5	3	329	36	5.9	11.76	3
B4DNG0	cDNA FLJ58142, highly similar to Olfactomedin-like protein 3 OS=Homo sapiens OX=9606 GN=OLFML3 PE=1 SV=1	23	5	13	5	345	39	6.11	31.76	5
B4DPQ0	Complement C1r subcomponent OS=Homo sapiens OX=9606 GN=C1R PE=1 SV=1	41	18	100	18	719	81.8	6.37	315.2	18
B4DPZ5	cDNA FLJ53495, highly similar to Polymerase I and transcript release factor OS=Homo sapiens OX=9606 PE=2 SV=1	8	2	5	2	363	40.5	5.25	11.93	2
B4DRT4	cDNA FLJ51535, highly similar to Phosphatidylethanolamine-binding protein 1 OS=Homo sapiens OX=9606 PE=2 SV=1	23	2	7	2	155	17.3	6	19.88	2
B4DRV4	cDNA FLJ55667, highly similar to Secreted protein acidic and rich in cysteine OS=Homo sapiens OX=9606 PE=2 SV=1	70	12	157	1	212	24.7	5.62	436.15	12
B4DU16	cDNA FLJ54550, highly similar to Homo sapiens fibronectin 1 (FN1), transcript variant 6, mRNA OS=Homo sapiens OX=9606 PE=2 SV=1	61	31	443	1	894	98.1	6.24	1410.81	31
B4DUQ1	cDNA FLJ54552, highly similar to Heterogeneous nuclear ribonucleoprotein K OS=Homo sapiens OX=9606 PE=2 SV=1	16	5	11	5	439	48.5	5.92	28.68	5
B4DUV1	Fibulin-1 OS=Homo sapiens OX=9606 PE=2 SV=1	41	15	48	3	641	70.1	5.26	136.96	15
B4DWC4	Chloride intracellular channel protein OS=Homo sapiens OX=9606 PE=2 SV=1	35	6	14	6	245	27.8	6.38	33.94	6
B4E324	cDNA FLJ60397, highly similar to Lysosomal protective protein OS=Homo sapiens OX=9606 PE=2 SV=1	3	1	3	1	480	54.2	6.98	8	1
B4E3Q1	cDNA FLJ61580, highly similar to Calsyntenin-1 OS=Homo sapiens OX=9606 PE=2 SV=1	14	9	29	9	962	107.7	4.97	81.38	9
B5MCZ3	Interleukin-6 OS=Homo sapiens OX=9606 GN=IL6 PE=1 SV=1	38	6	30	6	189	21.5	6.57	85.17	6
B7Z1I4	cDNA FLJ57258, highly similar to Neurotrimin OS=Homo sapiens OX=9606 PE=2 SV=1	6	1	1	1	346	38.2	8.54	2.25	1
B7Z230	Peptidylprolyl isomerase OS=Homo sapiens OX=9606 PE=2 SV=1	2	1	1	1	432	47.8	4.91	1.9	1
C9IZP8	Complement C1s subcomponent (Fragment) OS=Homo sapiens OX=9606 GN=C1S PE=1 SV=1	17	1	3	1	103	11.8	4.2	9.53	1
C9JPM3	Disintegrin and metalloproteinase domain-containing protein 9 OS=Homo sapiens OX=9606 GN=ADAM9 PE=1 SV=1	11	1	1	1	147	16.8	8.95	2.62	1
D0PNI1	Epididymis luminal protein 4 OS=Homo sapiens OX=9606 GN=YWHAZ PE=2 SV=1	36	7	18	6	245	27.7	4.79	48.59	7
D0PNI2	Lysyl oxidase OS=Homo sapiens OX=9606 GN=LOX PE=4 SV=1	24	6	11	6	417	46.9	8.09	30.06	6

Accession	Description	Coverage [%][a]	# Peptides[b]	# PSMs[c]	# Unique Peptides[d]	# AAs[e]	MW [kDa]	Calc. pI[f]	Score Sequest HT[g]	# Peptides: Sequest HT[h]
D1MGQ2	Alpha-2 globin chain OS=Homo sapiens OX=9606 GN=HBA2 PE=3 SV=1	11	1	1	1	142	15.2	8.68	3.16	1
D3DT71	Collagen, type XI, alpha 1, isoform CRA_b OS=Homo sapiens OX=9606 GN=COL11A1 PE=4 SV=1	2	2	5	2	1767	176.5	5.38	13.82	2
D3DTX7	Collagen, type I, alpha 1, isoform CRA_a OS=Homo sapiens OX=9606 GN=COL1A1 PE=4 SV=1	67	33	344	2	885	84.7	6.24	1085.9	33
D3YTG3	Target of Nesh-SH3 OS=Homo sapiens OX=9606 GN=ABI3BP PE=1 SV=1	5	6	15	6	1777	195.2	9.73	40.29	6
D6RF35	Vitamin D-binding protein OS=Homo sapiens OX=9606 GN=GC PE=1 SV=1	5	1	1	1	476	53	5.52	2.75	1
D6RF92	C-X-C motif chemokine OS=Homo sapiens OX=9606 GN=CXCL6 PE=1 SV=1	29	4	16	4	113	11.9	9.76	38.25	4
D9ZGF2	Collagen, type VI, alpha 3 OS=Homo sapiens OX=9606 GN=COL6A3 PE=4 SV=1	25	54	141	54	3177	343.5	6.68	356.17	54
E7ETU9	Procollagen-lysine,2-oxoglutarate 5-dioxygenase 2 OS=Homo sapiens OX=9606 GN=PLOD2 PE=1 SV=1	6	3	5	3	703	81.1	6.65	10.65	3
E9PL57	NEDD8-MDP1 readthrough (Fragment) OS=Homo sapiens OX=9606 GN=NEDD8-MDP1 PE=4 SV=1	8	1	1	1	170	19.5	7.43	1.96	1
E9PRJ8	Tetraspanin (Fragment) OS=Homo sapiens OX=9606 GN=CD81 PE=1 SV=1	28	3	4	3	209	22.5	6.15	10.88	3
F2Z393	Transaldolase OS=Homo sapiens OX=9606 GN=TALDO1 PE=1 SV=1	8	2	2	2	318	35.3	8.97	4.66	2
F5GZS6	4F2 cell-surface antigen heavy chain OS=Homo sapiens OX=9606 GN=SLC3A2 PE=1 SV=1	2	1	1	1	599	64.8	5.1	1.92	1
F5H5D3	Tubulin alpha chain OS=Homo sapiens OX=9606 GN=TUBA1C PE=1 SV=1	21	7	18	7	519	57.7	5.07	46.79	7
F6RFD5	Destrin OS=Homo sapiens OX=9606 GN=DSTN PE=1 SV=1	18	2	4	2	135	15.4	8.59	10.24	2
F8W6I7	Heterogeneous nuclear ribonucleoprotein A1 OS=Homo sapiens OX=9606 GN=HNRNPA1 PE=1 SV=2	19	4	12	4	307	33.1	9.13	28.51	4
F8W809	Thioredoxin reductase 1, cytoplasmic OS=Homo sapiens OX=9606 GN=TXNRD1 PE=1 SV=1	6	2	3	2	498	54.6	6.47	7.15	2
G3V279	Enhancer of rudimentary homolog OS=Homo sapiens OX=9606 GN=ERH PE=1 SV=1	17	1	1	1	71	8.2	4.7	2.09	1
G3V3E8	NPC intracellular cholesterol transporter 2 OS=Homo sapiens OX=9606 GN=NPC2 PE=1 SV=1	28	3	9	3	174	19.2	8.44	23.6	3
H0UI49	Laminin, alpha 4, isoform CRA_b OS=Homo sapiens OX=9606 GN=LAMA4 PE=4 SV=1	19	21	44	21	1816	201.7	6.3	109.38	21
H0YGS3	Microfibrillar-associated protein 5 (Fragment) OS=Homo sapiens OX=9606 GN=MFAP5 PE=1 SV=1	34	2	8	2	79	9.4	9.47	21.68	2
H3BT71	RNA-binding motif protein, X chromosome OS=Homo sapiens OX=9606 GN=RBMX PE=1 SV=1	9	2	2	2	296	32.2	9.91	4.5	2
H6VRF8	Keratin 1 OS=Homo sapiens OX=9606 GN=KRT1 PE=3 SV=1	15	8	14	8	644	66	8.12	32.02	8
H7BZJ3	Protein disulfide-isomerase A3 (Fragment) OS=Homo sapiens OX=9606 GN=PDIA3 PE=1 SV=1	20	2	4	1	123	13.5	7.3	8.7	2
H7C0V9	Amyloid-beta A4 protein (Fragment) OS=Homo sapiens OX=9606 GN=APP PE=1 SV=1	4	1	1	1	485	55.1	4.82	3.35	1
H7C557	Multiple epidermal growth factor-like domains protein 6 OS=Homo sapiens OX=9606 GN=MEGF6 PE=1 SV=2	1	1	3	1	1323	138.3	6.62	8.84	1
I3L297	Adapter molecule crk OS=Homo sapiens OX=9606 GN=CRK PE=1 SV=1	5	1	2	1	223	25.5	7.56	3.99	1
I4AY87	Macrophage migration inhibitory factor (Fragment) OS=Homo sapiens OX=9606 GN=MIF PE=1 SV=1	18	2	4	2	115	12.5	7.88	9.04	2
K4RH61	Matrix metalloproteinase 14 (Membrane-inserted) OS=Homo sapiens OX=9606 GN=MMP14 PE=2 SV=1	2	1	3	1	582	65.9	7.97	7.14	1

Accession	Description	Coverage [%][a]	# Peptides[b]	# PSMs[c]	# Unique Peptides[d]	# AAs[e]	MW [kDa]	Calc. pI[f]	Score Sequest HT[g]	# Peptides: Sequest HT[h]
K7ENT6	Tropomyosin alpha-4 chain OS=Homo sapiens OX=9606 GN=TPM4 PE=1 SV=2	26	7	33	1	247	28.5	4.63	85.63	7
K7EQA1	Programmed cell death protein 5 OS=Homo sapiens OX=9606 GN=PDCD5 PE=1 SV=1	15	1	2	1	87	10	9	4.41	1
M0R0Y4	Urokinase plasminogen activator surface receptor (Fragment) OS=Homo sapiens OX=9606 GN=PLAUR PE=1 SV=1	6	1	1	1	185	20.7	6.6	2.31	1
M0R261	6-phosphogluconolactonase (Fragment) OS=Homo sapiens OX=9606 GN=PGLS PE=1 SV=1	6	1	1	1	216	23	5.86	2.08	1
M1VE83	Tyrosine-protein kinase receptor OS=Homo sapiens OX=9606 GN=SDC4-ROS1_S4;R34 PE=2 SV=1	4	2	3	2	643	71.6	4.81	7.05	2
O00300	Tumor necrosis factor receptor superfamily member 11B OS=Homo sapiens OX=9606 GN=TNFRSF11B PE=1 SV=3	14	4	16	4	401	46	8.29	41.7	4
O00391	Sulfhydryl oxidase 1 OS=Homo sapiens OX=9606 GN=QSOX1 PE=1 SV=3	42	22	101	22	747	82.5	8.92	320.09	22
O14907	Tax1-binding protein 3 OS=Homo sapiens OX=9606 GN=TAX1BP3 PE=1 SV=2	28	2	4	2	124	13.7	8.48	8.95	2
O14950	Myosin regulatory light chain 12B OS=Homo sapiens OX=9606 GN=MYL12B PE=1 SV=2	34	5	10	2	172	19.8	4.84	23.11	5
O15511	Actin-related protein 2/3 complex subunit 5 OS=Homo sapiens OX=9606 GN=ARPC5 PE=1 SV=3	8	1	3	1	151	16.3	5.67	6.52	1
O43854	EGF-like repeat and discoidin I-like domain-containing protein 3 OS=Homo sapiens OX=9606 GN=EDIL3 PE=1 SV=1	17	5	9	5	480	53.7	7.28	20.14	5
O60568	Procollagen-lysine,2-oxoglutarate 5-dioxygenase 3 OS=Homo sapiens OX=9606 GN=PLOD3 PE=1 SV=1	6	3	5	3	738	84.7	6.05	11.43	3
O60664	Perilipin-3 OS=Homo sapiens OX=9606 GN=PLIN3 PE=1 SV=3	17	5	7	5	434	47	5.44	15.76	5
O95450	A disintegrin and metalloproteinase with thrombospondin motifs 2 OS=Homo sapiens OX=9606 GN=ADAMTS2 PE=2 SV=2	1	1	1	1	1211	134.7	7.15	2.32	1
P00338	L-lactate dehydrogenase A chain OS=Homo sapiens OX=9606 GN=LDHA PE=1 SV=2	20	5	17	5	332	36.7	8.27	42.29	5
P00441	Superoxide dismutase [Cu-Zn] OS=Homo sapiens OX=9606 GN=SOD1 PE=1 SV=2	25	2	4	2	154	15.9	6.13	10.38	2
P01024	Complement C3 OS=Homo sapiens OX=9606 GN=C3 PE=1 SV=2	38	45	144	45	1663	187	6.4	392.5	45
P01033	Metalloproteinase inhibitor 1 OS=Homo sapiens OX=9606 GN=TIMP1 PE=1 SV=1	54	7	103	7	207	23.2	8.1	350.42	7
P01034	Cystatin-C OS=Homo sapiens OX=9606 GN=CST3 PE=1 SV=1	42	5	15	5	146	15.8	8.75	40.11	5
P02452	Collagen alpha-1(I) chain OS=Homo sapiens OX=9606 GN=COL1A1 PE=1 SV=5	55	43	417	12	1464	138.9	5.8	1306.75	43
P02461	Collagen alpha-1(III) chain OS=Homo sapiens OX=9606 GN=COL3A1 PE=1 SV=4	33	25	126	25	1466	138.5	6.61	379.31	25
P02462	Collagen alpha-1(IV) chain OS=Homo sapiens OX=9606 GN=COL4A1 PE=1 SV=4	8	6	23	6	1669	160.5	8.28	63.93	6
P02545	Prelamin-A/C OS=Homo sapiens OX=9606 GN=LMNA PE=1 SV=1	28	14	32	14	664	74.1	7.02	82.11	14
P04083	Annexin A1 OS=Homo sapiens OX=9606 GN=ANXA1 PE=1 SV=2	33	9	22	9	346	38.7	7.02	54.48	9
P04632	Calpain small subunit 1 OS=Homo sapiens OX=9606 GN=CAPNS1 PE=1 SV=1	5	1	3	1	268	28.3	5.2	6.74	1
P04792	Heat shock protein beta-1 OS=Homo sapiens OX=9606 GN=HSPB1 PE=1 SV=2	23	2	5	2	205	22.8	6.4	13.37	2
P05120	Plasminogen activator inhibitor 2 OS=Homo sapiens OX=9606 GN=SERPINB2 PE=1 SV=2	7	2	2	2	415	46.6	5.63	4.58	2
P05387	60S acidic ribosomal protein P2 OS=Homo sapiens OX=9606 GN=RPLP2 PE=1 SV=1	14	2	2	2	115	11.7	4.54	4.47	2

Accession	Description	Coverage [%][a]	# Peptides[b]	# PSMs[c]	# Unique Peptides[d]	# AAs[e]	MW [kDa]	Calc. pI[f]	Score Sequest HT[g]	# Peptides: Sequest HT[h]
P05997	Collagen alpha-2(V) chain OS=Homo sapiens OX=9606 GN=COL5A2 PE=1 SV=3	32	24	73	24	1499	144.8	6.46	213.93	24
P07195	L-lactate dehydrogenase B chain OS=Homo sapiens OX=9606 GN=LDHB PE=1 SV=2	18	5	7	5	334	36.6	6.05	16.11	5
P07437	Tubulin beta chain OS=Homo sapiens OX=9606 GN=TUBB PE=1 SV=2	30	8	16	8	444	49.6	4.89	41.98	8
P07585	Decorin OS=Homo sapiens OX=9606 GN=DCN PE=1 SV=1	36	10	88	10	359	39.7	8.54	248.76	10
P07737	Profilin-1 OS=Homo sapiens OX=9606 GN=PFN1 PE=1 SV=2	59	5	18	5	140	15	8.27	48.69	5
P07942	Laminin subunit beta-1 OS=Homo sapiens OX=9606 GN=LAMB1 PE=1 SV=2	17	21	38	21	1786	197.9	4.94	89.95	21
P07996	Thrombospondin-1 OS=Homo sapiens OX=9606 GN=THBS1 PE=1 SV=2	39	31	125	29	1170	129.3	4.94	352.2	31
P08254	Stromelysin-1 OS=Homo sapiens OX=9606 GN=MMP3 PE=1 SV=2	14	6	19	6	477	53.9	6.16	46.93	6
P08670	Vimentin OS=Homo sapiens OX=9606 GN=VIM PE=1 SV=4	49	23	109	23	466	53.6	5.12	298.9	23
P08758	Annexin A5 OS=Homo sapiens OX=9606 GN=ANXA5 PE=1 SV=2	14	4	5	4	320	35.9	5.05	10.72	4
P09341	Growth-regulated alpha protein OS=Homo sapiens OX=9606 GN=CXCL1 PE=1 SV=1	37	3	36	2	107	11.3	10.43	122.72	3
P09382	Galectin-1 OS=Homo sapiens OX=9606 GN=LGALS1 PE=1 SV=2	65	5	26	5	135	14.7	5.5	73.82	5
P09486	SPARC OS=Homo sapiens OX=9606 GN=SPARC PE=1 SV=1	56	13	195	2	303	34.6	4.84	551.28	13
P09871	Complement C1s subcomponent OS=Homo sapiens OX=9606 GN=C1S PE=1 SV=1	36	16	65	16	688	76.6	4.96	190.03	16
P10599	Thioredoxin OS=Homo sapiens OX=9606 GN=TXN PE=1 SV=3	12	1	5	1	105	11.7	4.92	12.69	1
P10909	Clusterin OS=Homo sapiens OX=9606 GN=CLU PE=1 SV=1	22	7	21	7	449	52.5	6.27	53.88	7
P11047	Laminin subunit gamma-1 OS=Homo sapiens OX=9606 GN=LAMC1 PE=1 SV=3	19	20	50	20	1609	177.5	5.12	138.63	20
P12110	Collagen alpha-2(VI) chain OS=Homo sapiens OX=9606 GN=COL6A2 PE=1 SV=4	23	14	45	2	1019	108.5	6.21	126.68	14
P13497	Bone morphogenetic protein 1 OS=Homo sapiens OX=9606 GN=BMP1 PE=1 SV=2	8	6	14	6	986	111.2	6.9	33.68	6
P13500	C-C motif chemokine 2 OS=Homo sapiens OX=9606 GN=CCL2 PE=1 SV=1	11	1	8	1	99	11	9.25	18.95	1
P13639	Elongation factor 2 OS=Homo sapiens OX=9606 GN=EEF2 PE=1 SV=4	12	7	15	7	858	95.3	6.83	36.42	7
P14543	Nidogen-1 OS=Homo sapiens OX=9606 GN=NID1 PE=1 SV=3	13	12	27	11	1247	136.3	5.29	68.38	12
P14550	Alcohol dehydrogenase [NADP(+)] OS=Homo sapiens OX=9606 GN=AKR1A1 PE=1 SV=3	5	1	1	1	325	36.6	6.79	2.01	1
P15018	Leukemia inhibitory factor OS=Homo sapiens OX=9606 GN=LIF PE=1 SV=1	15	3	7	3	202	22	9.35	17.55	3
P15291	Beta-1,4-galactosyltransferase 1 OS=Homo sapiens OX=9606 GN=B4GALT1 PE=1 SV=5	31	7	13	7	398	43.9	8.65	34.62	7
P18065	Insulin-like growth factor-binding protein 2 OS=Homo sapiens OX=9606 GN=IGFBP2 PE=1 SV=2	33	7	24	7	325	34.8	7.5	62.35	7
P18206	Vinculin OS=Homo sapiens OX=9606 GN=VCL PE=1 SV=4	18	16	30	16	1134	123.7	5.66	73.5	16
P19022	Cadherin-2 OS=Homo sapiens OX=9606 GN=CDH2 PE=1 SV=4	8	6	17	6	906	99.7	4.81	42.93	6

Accession	Description	Coverage [%][a]	# Peptides[b]	# PSMs[c]	# Unique Peptides[d]	# AAs[e]	MW [kDa]	Calc. pI[f]	Score Sequest HT[g]	# Peptides: Sequest HT[h]
P19320	Vascular cell adhesion protein 1 OS=Homo sapiens OX=9606 GN=VCAM1 PE=1 SV=1	26	15	31	15	739	81.2	5.22	72.06	15
P19875	C-X-C motif chemokine 2 OS=Homo sapiens OX=9606 GN=CXCL2 PE=1 SV=1	37	3	33	1	107	11.4	10.55	113.11	3
P19876	C-X-C motif chemokine 3 OS=Homo sapiens OX=9606 GN=CXCL3 PE=1 SV=1	38	3	35	1	107	11.3	10.37	119.5	3
P20618	Proteasome subunit beta type-1 OS=Homo sapiens OX=9606 GN=PSMB1 PE=1 SV=2	6	1	1	1	241	26.5	8.13	2.01	1
P20809	Interleukin-11 OS=Homo sapiens OX=9606 GN=IL11 PE=1 SV=1	27	4	8	4	199	21.4	10.62	16.86	4
P21333	Filamin-A OS=Homo sapiens OX=9606 GN=FLNA PE=1 SV=4	38	59	171	57	2647	280.6	6.06	474.63	59
P23526	Adenosylhomocysteinase OS=Homo sapiens OX=9606 GN=AHCY PE=1 SV=4	9	3	6	3	432	47.7	6.34	13.6	3
P24592	Insulin-like growth factor-binding protein 6 OS=Homo sapiens OX=9606 GN=IGFBP6 PE=1 SV=1	22	2	4	2	240	25.3	7.81	15.13	2
P24844	Myosin regulatory light polypeptide 9 OS=Homo sapiens OX=9606 GN=MYL9 PE=1 SV=4	34	5	10	2	172	19.8	4.92	22.54	5
P25398	40S ribosomal protein S12 OS=Homo sapiens OX=9606 GN=RPS12 PE=1 SV=3	25	2	4	2	132	14.5	7.21	11.11	2
P25786	Proteasome subunit alpha type-1 OS=Homo sapiens OX=9606 GN=PSMA1 PE=1 SV=1	5	1	1	1	263	29.5	6.61	2.02	1
P26022	Pentraxin-related protein PTX3 OS=Homo sapiens OX=9606 GN=PTX3 PE=1 SV=3	39	11	39	11	381	41.9	5.01	107.16	11
P26038	Moesin OS=Homo sapiens OX=9606 GN=MSN PE=1 SV=3	19	9	29	7	577	67.8	6.4	74.38	9
P26641	Elongation factor 1-gamma OS=Homo sapiens OX=9606 GN=EEF1G PE=1 SV=3	3	1	3	1	437	50.1	6.67	6.05	1
P27816	Microtubule-associated protein 4 OS=Homo sapiens OX=9606 GN=MAP4 PE=1 SV=3	9	7	11	7	1152	120.9	5.43	25.6	7
P28072	Proteasome subunit beta type-6 OS=Homo sapiens OX=9606 GN=PSMB6 PE=1 SV=4	5	1	1	1	239	25.3	4.92	2.32	1
P30041	Peroxiredoxin-6 OS=Homo sapiens OX=9606 GN=PRDX6 PE=1 SV=3	19	3	8	3	224	25	6.38	20.01	3
P30044	Peroxiredoxin-5, mitochondrial OS=Homo sapiens OX=9606 GN=PRDX5 PE=1 SV=4	14	2	3	2	214	22.1	8.7	8.11	2
P30050	60S ribosomal protein L12 OS=Homo sapiens OX=9606 GN=RPL12 PE=1 SV=1	15	2	3	2	165	17.8	9.42	6.24	2
P30530	Tyrosine-protein kinase receptor UFO OS=Homo sapiens OX=9606 GN=AXL PE=1 SV=4	3	2	3	2	894	98.3	5.39	7.03	2
P31946	14-3-3 protein beta/alpha OS=Homo sapiens OX=9606 GN=YWHAB PE=1 SV=3	12	2	4	1	246	28.1	4.83	8.78	2
P33908	Mannosyl-oligosaccharide 1,2-alpha-mannosidase IA OS=Homo sapiens OX=9606 GN=MAN1A1 PE=1 SV=3	10	5	5	5	653	72.9	6.47	12.14	5
P35052	Glypican-1 OS=Homo sapiens OX=9606 GN=GPC1 PE=1 SV=2	23	9	19	9	558	61.6	7.3	46.87	9
P35442	Thrombospondin-2 OS=Homo sapiens OX=9606 GN=THBS2 PE=1 SV=2	24	18	60	16	1172	129.9	4.83	158.75	18
P35527	Keratin, type I cytoskeletal 9 OS=Homo sapiens OX=9606 GN=KRT9 PE=1 SV=3	4	2	4	2	623	62	5.24	10.87	2
P35555	Fibrillin-1 OS=Homo sapiens OX=9606 GN=FBN1 PE=1 SV=3	25	46	137	46	2871	312	4.93	363.16	46
P36955	Pigment epithelium-derived factor OS=Homo sapiens OX=9606 GN=SERPINF1 PE=1 SV=4	28	9	23	9	418	46.3	6.38	58.44	9
P37802	Transgelin-2 OS=Homo sapiens OX=9606 GN=TAGLN2 PE=1 SV=3	50	8	26	8	199	22.4	8.25	72.22	8

Accession	Description	Coverage [%][a]	# Peptides[b]	# PSMs[c]	# Unique Peptides[d]	# AAs[e]	MW [kDa]	Calc. pI[f]	Score Sequest HT[g]	# Peptides: Sequest HT[h]
P40925	Malate dehydrogenase, cytoplasmic OS=Homo sapiens OX=9606 GN=MDH1 PE=1 SV=4	12	3	5	1	334	36.4	7.36	11.24	3
P41222	Prostaglandin-H2 D-isomerase OS=Homo sapiens OX=9606 GN=PTGDS PE=1 SV=1	17	2	4	2	190	21	7.8	9.87	2
P43251	Biotinidase OS=Homo sapiens OX=9606 GN=BTD PE=1 SV=2	7	3	6	3	543	61.1	6.25	15.63	3
P46926	Glucosamine-6-phosphate isomerase 1 OS=Homo sapiens OX=9606 GN=GNPDA1 PE=1 SV=1	4	1	1	1	289	32.6	6.92	2.02	1
P48061	Stromal cell-derived factor 1 OS=Homo sapiens OX=9606 GN=CXCL12 PE=1 SV=1	24	2	7	2	93	10.7	9.88	16.72	2
P48307	Tissue factor pathway inhibitor 2 OS=Homo sapiens OX=9606 GN=TFPI2 PE=1 SV=1	13	2	5	2	235	26.9	8.53	13.13	2
P48723	Heat shock 70 kDa protein 13 OS=Homo sapiens OX=9606 GN=HSPA13 PE=1 SV=1	3	1	1	1	471	51.9	5.76	2.43	1
P50990	T-complex protein 1 subunit theta OS=Homo sapiens OX=9606 GN=CCT8 PE=1 SV=4	4	2	4	2	548	59.6	5.6	8.48	2
P51884	Lumican OS=Homo sapiens OX=9606 GN=LUM PE=1 SV=2	35	10	95	10	338	38.4	6.61	284.9	10
P51991	Heterogeneous nuclear ribonucleoprotein A3 OS=Homo sapiens OX=9606 GN=HNRNPA3 PE=1 SV=2	3	1	3	1	378	39.6	9.01	6.59	1
P54578	Ubiquitin carboxyl-terminal hydrolase 14 OS=Homo sapiens OX=9606 GN=USP14 PE=1 SV=3	3	1	1	1	494	56	5.3	2.1	1
P54802	Alpha-N-acetylglucosaminidase OS=Homo sapiens OX=9606 GN=NAGLU PE=1 SV=2	7	3	6	3	743	82.2	6.65	13.82	3
P55058	Phospholipid transfer protein OS=Homo sapiens OX=9606 GN=PLTP PE=1 SV=1	13	5	14	5	493	54.7	7.01	36.17	5
P55072	Transitional endoplasmic reticulum ATPase OS=Homo sapiens OX=9606 GN=VCP PE=1 SV=4	13	8	11	8	806	89.3	5.26	23.33	8
P55290	Cadherin-13 OS=Homo sapiens OX=9606 GN=CDH13 PE=1 SV=1	7	4	8	4	713	78.2	4.98	18.99	4
P58546	Myotrophin OS=Homo sapiens OX=9606 GN=MTPN PE=1 SV=2	14	1	1	1	118	12.9	5.52	2.01	1
P59998	Actin-related protein 2/3 complex subunit 4 OS=Homo sapiens OX=9606 GN=ARPC4 PE=1 SV=3	11	2	4	2	168	19.7	8.43	8.87	2
P62328	Thymosin beta-4 OS=Homo sapiens OX=9606 GN=TMSB4X PE=1 SV=2	34	2	4	2	44	5.1	5.06	9.81	2
P62857	40S ribosomal protein S28 OS=Homo sapiens OX=9606 GN=RPS28 PE=1 SV=1	13	1	1	1	69	7.8	10.7	1.9	1
P62906	60S ribosomal protein L10a OS=Homo sapiens OX=9606 GN=RPL10A PE=1 SV=2	6	1	1	1	217	24.8	9.94	2.53	1
P62937	Peptidyl-prolyl cis-trans isomerase A OS=Homo sapiens OX=9606 GN=PPIA PE=1 SV=2	32	3	6	3	165	18	7.81	14.61	3
P62942	Peptidyl-prolyl cis-trans isomerase FKBP1A OS=Homo sapiens OX=9606 GN=FKBP1A PE=1 SV=2	26	2	6	2	108	11.9	8.16	15.41	2
P63220	40S ribosomal protein S21 OS=Homo sapiens OX=9606 GN=RPS21 PE=1 SV=1	30	2	2	2	83	9.1	8.5	3.96	2
P63313	Thymosin beta-10 OS=Homo sapiens OX=9606 GN=TMSB10 PE=1 SV=2	34	1	5	1	44	5	5.36	13.45	1
P67936	Tropomyosin alpha-4 chain OS=Homo sapiens OX=9606 GN=TPM4 PE=1 SV=3	31	8	39	2	248	28.5	4.69	98.37	8
P80303	Nucleobindin-2 OS=Homo sapiens OX=9606 GN=NUCB2 PE=1 SV=3	10	3	9	3	420	50.2	5.12	23.42	3
P80723	Brain acid soluble protein 1 OS=Homo sapiens OX=9606 GN=BASP1 PE=1 SV=2	12	1	1	1	227	22.7	4.63	2.52	1
P98066	Tumor necrosis factor-inducible gene 6 protein OS=Homo sapiens OX=9606 GN=TNFAIP6 PE=1 SV=2	3	1	2	1	277	31.2	6.79	4.35	1

Accession	Description	Coverage [%][a]	# Peptides[b]	# PSMs[c]	# Unique Peptides[d]	# AAs[e]	MW [kDa]	Calc. pI[f]	Score Sequest HT[g]	# Peptides: Sequest HT[h]
Q01995	Transgelin OS=Homo sapiens OX=9606 GN=TAGLN PE=1 SV=4	54	9	49	9	201	22.6	8.84	124.98	9
Q02388	Collagen alpha-1(VII) chain OS=Homo sapiens OX=9606 GN=COL7A1 PE=1 SV=2	10	15	26	15	2944	295	6.27	63.23	15
Q04760	Lactoylglutathione lyase OS=Homo sapiens OX=9606 GN=GLO1 PE=1 SV=4	15	2	2	2	184	20.8	5.31	4.45	2
Q05DB4	HEBP2 protein (Fragment) OS=Homo sapiens OX=9606 GN=HEBP2 PE=2 SV=1	7	1	1	1	214	24	5.38	2.55	1
Q05DH1	Proteasome subunit alpha type (Fragment) OS=Homo sapiens OX=9606 GN=PSMA7 PE=2 SV=1	6	1	1	1	238	26.7	8.87	1.9	1
Q06210	Glutamine--fructose-6-phosphate aminotransferase [isomerizing] 1 OS=Homo sapiens OX=9606 GN=GFPT1 PE=1 SV=3	4	2	4	2	699	78.8	7.11	8.34	2
Q07954	Prolow-density lipoprotein receptor-related protein 1 OS=Homo sapiens OX=9606 GN=LRP1 PE=1 SV=2	4	13	22	13	4544	504.3	5.39	52.1	13
Q08629	Testican-1 OS=Homo sapiens OX=9606 GN=SPOCK1 PE=1 SV=1	12	4	13	4	439	49.1	6.1	32.7	4
Q09666	Neuroblast differentiation-associated protein AHNAK OS=Homo sapiens OX=9606 GN=AHNAK PE=1 SV=2	12	32	57	32	5890	628.7	6.15	124.56	32
Q0Z944	Beta globin (Fragment) OS=Homo sapiens OX=9606 GN=HBB PE=3 SV=1	10	1	1	1	105	11.5	6.37	2.16	1
Q10471	Polypeptide N-acetylgalactosaminyltransferase 2 OS=Homo sapiens OX=9606 GN=GALNT2 PE=1 SV=1	12	5	8	5	571	64.7	8.35	18.17	5
Q12841	Follistatin-related protein 1 OS=Homo sapiens OX=9606 GN=FSTL1 PE=1 SV=1	40	11	83	11	308	35	5.52	266.69	11
Q13217	DnaJ homolog subfamily C member 3 OS=Homo sapiens OX=9606 GN=DNAJC3 PE=1 SV=1	6	2	4	2	504	57.5	6.15	10.14	2
Q13219	Pappalysin-1 OS=Homo sapiens OX=9606 GN=PAPPA PE=1 SV=3	10	11	23	11	1627	180.9	6.18	56.02	11
Q13561	Dynactin subunit 2 OS=Homo sapiens OX=9606 GN=DCTN2 PE=1 SV=4	7	2	2	2	401	44.2	5.21	4.65	2
Q14112	Nidogen-2 OS=Homo sapiens OX=9606 GN=NID2 PE=1 SV=3	6	6	9	5	1375	151.2	5.29	19.46	6
Q14315	Filamin-C OS=Homo sapiens OX=9606 GN=FLNC PE=1 SV=3	5	10	20	8	2725	290.8	5.97	45.48	10
Q14393	Growth arrest-specific protein 6 OS=Homo sapiens OX=9606 GN=GAS6 PE=1 SV=3	18	7	18	7	678	74.9	5.69	49.4	7
Q14767	Latent-transforming growth factor beta-binding protein 2 OS=Homo sapiens OX=9606 GN=LTBP2 PE=1 SV=3	13	15	43	15	1821	194.9	5.19	114.24	15
Q15084	Protein disulfide-isomerase A6 OS=Homo sapiens OX=9606 GN=PDIA6 PE=1 SV=1	13	4	9	4	440	48.1	5.08	23.39	4
Q15149	Plectin OS=Homo sapiens OX=9606 GN=PLEC PE=1 SV=3	6	25	41	25	4684	531.5	5.96	92.87	25
Q15293	Reticulocalbin-1 OS=Homo sapiens OX=9606 GN=RCN1 PE=1 SV=1	7	2	3	2	331	38.9	5	5.89	2
Q15365	Poly(rC)-binding protein 1 OS=Homo sapiens OX=9606 GN=PCBP1 PE=1 SV=2	6	1	1	1	356	37.5	7.09	2.71	1
Q15582	Transforming growth factor-beta-induced protein ig-h3 OS=Homo sapiens OX=9606 GN=TGFBI PE=1 SV=1	47	18	136	18	683	74.6	7.71	422.91	18
Q16270	Insulin-like growth factor-binding protein 7 OS=Homo sapiens OX=9606 GN=IGFBP7 PE=1 SV=1	45	10	74	10	282	29.1	7.9	241.96	10
Q16394	Exostosin-1 OS=Homo sapiens OX=9606 GN=EXT1 PE=1 SV=2	5	3	5	3	746	86.2	9.04	11.18	3
Q16658	Fascin OS=Homo sapiens OX=9606 GN=FSCN1 PE=1 SV=3	12	4	8	4	493	54.5	7.24	24.25	4
Q16778	Histone H2B type 2-E OS=Homo sapiens OX=9606 GN=HIST2H2BE PE=1 SV=3	12	1	2	1	126	13.9	10.32	4.49	1

Accession	Description	Coverage [%][a]	# Peptides[b]	# PSMs[c]	# Unique Peptides[d]	# AAs[e]	MW [kDa]	Calc. pI[f]	Score Sequest HT[g]	# Peptides: Sequest HT[h]
Q2HIZ0	Thrombospondin 3 OS=Homo sapiens OX=9606 GN=THBS3 PE=2 SV=1	6	3	6	3	956	104.1	4.65	16.73	3
Q4ZHG4	Fibronectin type III domain-containing protein 1 OS=Homo sapiens OX=9606 GN=FNDC1 PE=2 SV=4	14	19	55	19	1894	205.4	9.32	145.42	19
Q53FA4	Cysteine-rich, angiogenic inducer, 61 variant (Fragment) OS=Homo sapiens OX=9606 PE=2 SV=1	18	4	10	4	381	42	8.31	24.61	4
Q53FA7	Quinone oxidoreductase PIG3 OS=Homo sapiens OX=9606 GN=TP53I3 PE=1 SV=2	4	1	2	1	332	35.5	7.17	3.9	1
Q53G71	Calreticulin variant (Fragment) OS=Homo sapiens OX=9606 PE=2 SV=1	12	3	8	3	406	46.9	4.45	19.71	3
Q53G75	Matrix metalloproteinase 1 preproprotein variant (Fragment) OS=Homo sapiens OX=9606 PE=2 SV=1	25	6	12	6	405	46.5	7.03	29.26	6
Q53G99	Beta actin variant (Fragment) OS=Homo sapiens OX=9606 PE=2 SV=1	42	10	55	1	375	41.7	5.59	167.43	10
Q53GN4	WD repeat domain 1, isoform CRA_a (Fragment) OS=Homo sapiens OX=9606 GN=WDR1 PE=2 SV=1	10	4	6	4	606	66.1	6.65	12.39	4
Q53GY0	Plastin 3 variant (Fragment) OS=Homo sapiens OX=9606 PE=2 SV=1	22	9	17	9	630	70.7	5.68	42.21	9
Q53HQ7	Elongation factor 1-alpha (Fragment) OS=Homo sapiens OX=9606 PE=2 SV=1	16	4	9	4	462	50.2	8.94	24.73	4
Q53R94	Uncharacterized protein RTN4 (Fragment) OS=Homo sapiens OX=9606 GN=RTN4 PE=4 SV=1	7	1	2	1	185	19.3	4.13	4.21	1
Q53XB4	Full-length cDNA clone CS0DF032YM23 of Fetal brain of Homo sapiens (human) OS=Homo sapiens OX=9606 GN=RAB1 PE=2 SV=1	12	2	2	2	147	16.8	9.03	5.18	2
Q59GA0	Thy-1 cell surface antigen variant (Fragment) OS=Homo sapiens OX=9606 PE=2 SV=1	10	1	3	1	145	15.9	9	7.18	1
Q5M8T4	Connective tissue growth factor OS=Homo sapiens OX=9606 GN=CTGF PE=2 SV=1	34	8	16	8	349	38	7.94	40.13	8
Q5NKV8	Intercellular adhesion molecule 1 OS=Homo sapiens OX=9606 GN=ICAM1 PE=2 SV=1	9	3	9	3	532	57.8	7.68	22.68	3
Q5U0A0	Proteasome subunit alpha type OS=Homo sapiens OX=9606 PE=2 SV=1	5	1	3	1	241	26.4	4.79	6.58	1
Q641Q3	Meteorin-like protein OS=Homo sapiens OX=9606 GN=METRNL PE=2 SV=1	16	3	7	3	311	34.4	8.35	19.48	3
Q6DEN2	DPYSL3 protein OS=Homo sapiens OX=9606 GN=DPYSL3 PE=2 SV=1	6	3	6	2	684	73.9	6.55	13.85	3
Q6EMK4	Vasorin OS=Homo sapiens OX=9606 GN=VASN PE=1 SV=1	6	3	9	3	673	71.7	7.39	27.25	3
Q6FGX9	Adenylate kinase isoenzyme 1 OS=Homo sapiens OX=9606 GN=AK1 PE=2 SV=1	6	1	1	1	194	21.6	8.63	1.92	1
Q6FHC9	STC2 protein (Fragment) OS=Homo sapiens OX=9606 GN=STC2 PE=2 SV=1	27	4	24	4	302	33.2	7.3	89.57	4
Q6FHE1	FST protein OS=Homo sapiens OX=9606 GN=FST PE=2 SV=1	33	6	14	6	317	34.8	7.65	37.08	6
Q6FHF5	Proliferating cell nuclear antigen (Fragment) OS=Homo sapiens OX=9606 GN=PCNA PE=2 SV=1	6	1	1	1	261	28.7	4.69	2.33	1
Q6FHU3	PSME1 protein (Fragment) OS=Homo sapiens OX=9606 GN=PSME1 PE=2 SV=1	5	1	2	1	249	28.7	6.02	4.34	1
Q6FHW3	DF protein OS=Homo sapiens OX=9606 GN=DF PE=2 SV=1	21	3	6	3	228	24.4	7.24	15.68	3
Q6FHZ0	Malate dehydrogenase OS=Homo sapiens OX=9606 GN=MDH2 PE=2 SV=1	12	3	6	3	338	35.5	8.68	12.54	3
Q6IAW5	CALU protein OS=Homo sapiens OX=9606 GN=CALU PE=2 SV=1	22	4	14	4	315	37.1	4.64	39.29	4
Q6NUL1	Exostoses (Multiple) 2 OS=Homo sapiens OX=9606 GN=EXT2 PE=2 SV=1	6	3	5	3	718	82.1	6.55	12.36	3

Accession	Description	Coverage [%][a]	# Peptides[b]	# PSMs[c]	# Unique Peptides[d]	# AAs[e]	MW [kDa]	Calc. pI[f]	Score Sequest HT[g]	# Peptides: Sequest HT[h]
Q6NUR7	Ezrin OS=Homo sapiens OX=9606 GN=EZR PE=2 SV=1	5	3	8	1	586	69.2	6.27	17.06	3
Q6NVI1	MARCKS protein (Fragment) OS=Homo sapiens OX=9606 GN=MARCKS PE=2 SV=1	20	1	4	1	157	14.9	4.79	15.47	1
Q6UVK1	Chondroitin sulfate proteoglycan 4 OS=Homo sapiens OX=9606 GN=CSPG4 PE=1 SV=2	1	2	5	2	2322	250.4	5.47	13.68	2
Q6YHK3	CD109 antigen OS=Homo sapiens OX=9606 GN=CD109 PE=1 SV=2	6	7	17	7	1445	161.6	5.85	40.61	7
Q6ZN40	Tropomyosin 1 (Alpha), isoform CRA_f OS=Homo sapiens OX=9606 GN=TPM1 PE=1 SV=1	25	8	23	1	326	37.4	4.72	60.79	8
Q75MU2	Uncharacterized protein WBSCR1 (Fragment) OS=Homo sapiens OX=9606 GN=WBSCR1 PE=4 SV=1	8	1	1	1	156	17.1	5.31	2.2	1
Q7Z2W2	Extracellular sulfatase OS=Homo sapiens OX=9606 GN=DKFZp686F13142 PE=2 SV=1	2	1	3	1	871	101	9.09	6.87	1
Q7Z7M9	Polypeptide N-acetylgalactosaminyltransferase 5 OS=Homo sapiens OX=9606 GN=GALNT5 PE=1 SV=1	9	7	17	7	940	106.2	9.47	38.46	7
Q86UY0	TXNDC5 protein OS=Homo sapiens OX=9606 GN=TXNDC5 PE=1 SV=1	3	1	2	1	360	40.3	5.83	4.3	1
Q86Y38	Xylosyltransferase 1 OS=Homo sapiens OX=9606 GN=XYLT1 PE=1 SV=1	8	6	9	6	959	107.5	9.22	19.42	6
Q86Z22	Epididymis secretory protein Li 297 OS=Homo sapiens OX=9606 GN=HEL-S-297 PE=2 SV=1	8	2	11	2	226	23.8	9.06	36.62	2
Q8IUX7	Adipocyte enhancer-binding protein 1 OS=Homo sapiens OX=9606 GN=AEBP1 PE=1 SV=1	18	14	36	14	1158	130.8	5.11	97.46	14
Q8N7G1	Purine nucleoside phosphorylase OS=Homo sapiens OX=9606 PE=2 SV=1	12	2	2	2	293	32.5	7.21	5.09	2
Q8NBJ4	Golgi membrane protein 1 OS=Homo sapiens OX=9606 GN=GOLM1 PE=1 SV=1	8	2	3	2	401	45.3	4.97	7.12	2
Q8TBR3	Fusion (Involved in t(1216) in malignant liposarcoma) OS=Homo sapiens OX=9606 GN=FUS PE=2 SV=1	2	1	2	1	526	53.4	9.38	4.17	1
Q8WUJ3	Cell migration-inducing and hyaluronan-binding protein OS=Homo sapiens OX=9606 GN=CEMIP PE=1 SV=2	5	5	6	5	1361	152.9	7.85	12.41	5
Q8WX93	Palladin OS=Homo sapiens OX=9606 GN=PALLD PE=1 SV=3	3	1	1	1	1383	150.5	7.09	2.64	1
Q92520	Protein FAM3C OS=Homo sapiens OX=9606 GN=FAM3C PE=1 SV=1	21	3	8	3	227	24.7	8.29	22.33	3
Q92626	Peroxidasin homolog OS=Homo sapiens OX=9606 GN=PXDN PE=1 SV=2	25	21	56	21	1479	165.2	7.17	149.32	21
Q969H8	Myeloid-derived growth factor OS=Homo sapiens OX=9606 GN=MYDGF PE=1 SV=1	9	1	3	1	173	18.8	6.68	7.17	1
Q96AY3	Peptidyl-prolyl cis-trans isomerase FKBP10 OS=Homo sapiens OX=9606 GN=FKBP10 PE=1 SV=1	20	9	17	9	582	64.2	5.62	36.06	9
Q96C90	Protein phosphatase 1 regulatory subunit 14B OS=Homo sapiens OX=9606 GN=PPP1R14B PE=1 SV=3	18	1	1	1	147	15.9	4.86	3.32	1
Q96D15	Reticulocalbin-3 OS=Homo sapiens OX=9606 GN=RCN3 PE=1 SV=1	13	3	9	3	328	37.5	4.89	20.55	3
Q96HC4	PDZ and LIM domain protein 5 OS=Homo sapiens OX=9606 GN=PDLIM5 PE=1 SV=5	12	5	11	5	596	63.9	8.21	24.88	5
Q99715	Collagen alpha-1(XII) chain OS=Homo sapiens OX=9606 GN=COL12A1 PE=1 SV=2	33	66	235	66	3063	332.9	5.53	643.46	66
Q99988	Growth/differentiation factor 15 OS=Homo sapiens OX=9606 GN=GDF15 PE=1 SV=3	5	1	2	1	308	34.1	9.66	4.54	1
Q9BRK3	Matrix remodeling-associated protein 8 OS=Homo sapiens OX=9606 GN=MXRA8 PE=1 SV=1	5	2	3	2	442	49.1	7.23	6.36	2
Q9BS26	Endoplasmic reticulum resident protein 44 OS=Homo sapiens OX=9606 GN=ERP44 PE=1 SV=1	4	1	1	1	406	46.9	5.26	2.21	1

Accession	Description	Coverage [%][a]	# Peptides[b]	# PSMs[c]	# Unique Peptides[d]	# AAs[e]	MW [kDa]	Calc. pI[f]	Score Sequest HT[g]	# Peptides: Sequest HT[h]
Q9H3K6	BolA-like protein 2 OS=Homo sapiens OX=9606 GN=BOLA2 PE=1 SV=1	19	1	1	1	86	10.1	6.52	2.84	1
Q9H4F8	SPARC-related modular calcium-binding protein 1 OS=Homo sapiens OX=9606 GN=SMOC1 PE=1 SV=1	3	1	3	1	434	48.1	8.22	10.04	1
Q9NQ30	Endothelial cell-specific molecule 1 OS=Homo sapiens OX=9606 GN=ESM1 PE=1 SV=2	7	1	1	1	184	20.1	7.34	1.9	1
Q9NQ81	Uridine diphospho-glucose dehydrogenase (Fragment) OS=Homo sapiens OX=9606 GN=GDH PE=4 SV=1	8	1	1	1	164	17.8	6.16	2.22	1
Q9NS15	Latent-transforming growth factor beta-binding protein 3 OS=Homo sapiens OX=9606 GN=LTBP3 PE=1 SV=4	1	1	1	1	1303	139.3	6.07	2.14	1
Q9UBX1	Cathepsin F OS=Homo sapiens OX=9606 GN=CTSF PE=1 SV=1	4	1	1	1	484	53.3	8.22	2.46	1
Q9Y240	C-type lectin domain family 11 member A OS=Homo sapiens OX=9606 GN=CLEC11A PE=1 SV=1	16	4	13	4	323	35.7	5.16	31.88	4
Q9Y490	Talin-1 OS=Homo sapiens OX=9606 GN=TLN1 PE=1 SV=3	4	7	12	7	2541	269.6	6.07	26.66	7
Q9Y6C2	EMILIN-1 OS=Homo sapiens OX=9606 GN=EMILIN1 PE=1 SV=3	15	8	13	8	1016	106.6	5.17	33.05	8
S4R3N1	HSPE1-MOB4 readthrough OS=Homo sapiens OX=9606 GN=HSPE1-MOB4 PE=3 SV=1	5	1	3	1	261	29.7	6.16	8.62	1
V9HW25	Epididymis secretory protein Li 273 OS=Homo sapiens OX=9606 GN=HEL-S-273 PE=2 SV=1	24	8	36	2	284	33	4.67	90.26	8
V9HW74	Ubiquitin carboxyl-terminal hydrolase OS=Homo sapiens OX=9606 GN=HEL-117 PE=2 SV=1	10	1	2	1	223	24.8	5.48	4.68	1
V9HW98	Epididymis luminal protein 2 OS=Homo sapiens OX=9606 GN=HEL2 PE=1 SV=1	47	9	19	8	255	29.2	4.74	45.64	9
V9HWB4	Epididymis secretory sperm binding protein Li 89n OS=Homo sapiens OX=9606 GN=HEL-S-89n PE=2 SV=1	18	8	26	7	654	72.3	5.16	72.46	8
V9HWB8	Pyruvate kinase OS=Homo sapiens OX=9606 GN=HEL-S-30 PE=1 SV=1	40	14	40	3	531	57.9	7.84	110.41	14
V9HWC6	Peptidyl-prolyl cis-trans isomerase OS=Homo sapiens OX=9606 GN=HEL-S-39 PE=2 SV=1	28	5	18	5	208	22.7	9.32	47.38	5
V9HWE8	Epididymis secretory sperm binding protein Li 47e OS=Homo sapiens OX=9606 GN=HEL-S-47e PE=2 SV=1	15	2	6	2	204	23.2	5.11	14.61	2
V9HWF4	Phosphoglycerate kinase OS=Homo sapiens OX=9606 GN=HEL-S-68p PE=2 SV=1	4	1	2	1	417	44.6	8.1	3.87	1
V9HWI5	Cofilin 1 (Non-muscle), isoform CRA_b OS=Homo sapiens OX=9606 GN=HEL-S-15 PE=2 SV=1	43	4	12	3	166	18.5	8.09	29.22	4
V9HWN7	Fructose-bisphosphate aldolase OS=Homo sapiens OX=9606 GN=HEL-S-87p PE=2 SV=1	22	5	18	5	364	39.4	8.09	53.84	5
V9HWP2	Epididymis luminal protein 35 OS=Homo sapiens OX=9606 GN=HEL-S-125m PE=2 SV=1	10	6	14	5	803	92.4	4.84	38.71	6
A0A024QYX3	RNA binding motif (RNP1, RRM) protein 3, isoform CRA_c OS=Homo sapiens OX=9606 GN=RBM3 PE=4 SV=1	11	1	2	1	157	17.2	8.91	5.46	1

[a] Coverage is the percentage of the sequence covered by identifications (PSMs only) from the included searches. [b] # Peptides is the number of distinct peptide sequences in the protein. [c] # PSMs is number of identified peptide spectrum matches identified from all included searches, including those redundantly identified. [d] # Unique peptide is the total number of distinct peptide sequences unique to the protein group. [e] #AAs is length of the protein sequence. [f] Calc pI (isoelectric point) number is theoretically calculated isoelectric point for the protein that is, the pH at which a particular molecule carries no net electrical charge. [g] Score Sequest HT is a protein score calculated by summing the individual scores for each peptide. The higher this score, the higher the individual score of the peptide, allowing for better identification. [h] # Peptides: Sequest HT is the number of distinct peptide sequences in the identified protein.

Supplementary Table 1-3: Detailed information on commonly secreted proteins by 3 adipose tissue (AT) mesenchymal stem cells (MSCs) under normal condition.

Accession	Description	Coverage [%][a]	# Peptides[b]	# PSMs[c]	# Unique Peptides[d]	# AAs[e]	MW [kDa]	Calc. pI[f]	Score Sequest HT[g]	# Peptides: Sequest HT[h]
A0A024QYT5	Serpin peptidase inhibitor, clade E (Nexin, plasminogen activator inhibitor type 1), member 1, isoform CRA_b OS=Homo sapiens OX=9606 GN=SERPINE1 PE=3 SV=1	33	10	28	10	402	45	7.2	79.82	10
A0A024R1N1	Myosin, heavy polypeptide 9, non-muscle, isoform CRA_a OS=Homo sapiens OX=9606 GN=MYH9 PE=3 SV=1	12	15	25	15	1960	226.4	5.6	62.73	15
A0A024R1U8	Insulin-like growth factor binding protein 4, isoform CRA_a OS=Homo sapiens OX=9606 GN=IGFBP4 PE=4 SV=1	25	5	12	5	258	27.9	7.15	29.76	5
A0A024R1Z6	Vesicle amine transport protein 1 homolog (T californica), isoform CRA_a OS=Homo sapiens OX=9606 GN=VAT1 PE=4 SV=1	17	4	4	4	393	41.9	6.29	10.02	4
A0A024R222	Phosphoserine aminotransferase OS=Homo sapiens OX=9606 GN=PSAT1 PE=3 SV=1	6	2	2	2	370	40.4	7.66	4.11	2
A0A024R2P0	40S ribosomal protein SA OS=Homo sapiens OX=9606 GN=RPSA PE=3 SV=1	15	3	4	3	295	32.8	4.87	8.73	3
A0A024R2W4	Dystroglycan 1 (Dystrophin-associated glycoprotein 1), isoform CRA_a OS=Homo sapiens OX=9606 GN=DAG1 PE=4 SV=1	4	2	3	2	895	97.5	8.56	7.62	2
A0A024R321	Filamin B, beta (Actin binding protein 278), isoform CRA_a OS=Homo sapiens OX=9606 GN=FLNB PE=4 SV=1	4	7	9	6	2622	280.3	5.94	19.6	7
A0A024R462	Fibronectin 1, isoform CRA_n OS=Homo sapiens OX=9606 GN=FN1 PE=4 SV=1	57	77	760	1	2355	259	5.73	2472.92	77
A0A024R498	Serpin peptidase inhibitor, clade E (Nexin, plasminogen activator inhibitor type 1), member 2, isoform CRA_b OS=Homo sapiens OX=9606 GN=SERPINE2 PE=3 SV=1	36	11	33	11	397	44	9.38	101	11
A0A024R694	Actinin, alpha 1, isoform CRA_a OS=Homo sapiens OX=9606 GN=ACTN1 PE=4 SV=1	36	24	41	14	892	103	5.41	101.82	24
A0A024R6K8	Tryptophanyl-tRNA synthetase, isoform CRA_a OS=Homo sapiens OX=9606 GN=WARS PE=3 SV=1	10	3	3	3	471	53.1	6.23	7	3
A0A024R6R4	Matrix metallopeptidase 2 (Gelatinase A, 72kDa gelatinase, 72kDa type IV collagenase), isoform CRA_a OS=Homo sapiens OX=9606 GN=MMP2 PE=3 SV=1	54	23	116	23	660	73.8	5.47	345.42	23
A0A024R8V7	TIMP metallopeptidase inhibitor 2, isoform CRA_b OS=Homo sapiens OX=9606 GN=TIMP2 PE=4 SV=1	41	6	29	6	179	20.2	7.17	84.66	6
A0A024R962	HCG40889, isoform CRA_b OS=Homo sapiens OX=9606 GN=hCG_40889 PE=4 SV=1	10	9	16	9	1231	139	6.62	39.27	9
A0A024RA94	Microfibrillar-associated protein 2, isoform CRA_d OS=Homo sapiens OX=9606 GN=MFAP2 PE=4 SV=1	7	1	2	1	183	20.8	4.97	4.11	1
A0A024RAM4	Microtubule-associated protein 1B, isoform CRA_b OS=Homo sapiens OX=9606 GN=MAP1B PE=4 SV=1	1	1	1	1	2468	270.5	4.81	0	1
A0A024RD80	Heat shock protein 90kDa alpha (Cytosolic), class B member 1, isoform CRA_a OS=Homo sapiens OX=9606 GN=HSP90AB1 PE=3 SV=1	5	3	4	2	724	83.2	5.03	8.17	3
A0A024RDW8	Collagen, type IV, alpha 2, isoform CRA_a OS=Homo sapiens OX=9606 GN=COL4A2 PE=4 SV=1	20	18	36	18	1712	167.4	8.66	102.54	18
A0A087WTA8	Collagen alpha-2(I) chain OS=Homo sapiens OX=9606 GN=COL1A2 PE=1 SV=1	60	42	1088	42	1364	129.1	9.01	3539.78	42
A0A087WVQ6	Clathrin heavy chain OS=Homo sapiens OX=9606 GN=CLTC PE=1 SV=1	5	4	4	4	1679	191.9	5.69	10.16	4
A0A087WX80	Laminin subunit alpha-2 OS=Homo sapiens OX=9606 GN=LAMA2 PE=1 SV=1	7	14	20	14	3121	343.5	6.39	46.89	14
A0A087WZ51	Tumor protein D54 OS=Homo sapiens OX=9606 GN=TPD52L2 PE=1 SV=1	23	2	2	2	152	16.3	4.92	3.99	2
A0A087X0S5	Collagen alpha-1(VI) chain OS=Homo sapiens OX=9606 GN=COL6A1 PE=1 SV=1	46	30	200	30	1026	108.3	5.43	595.93	30
A0A090N8Y2	Protein disulfide-isomerase A4 OS=Homo sapiens OX=9606 GN=ERP70 PE=2 SV=1	2	1	1	1	645	72.9	5.07	2.14	1
A0A0A0MT01	Gelsolin OS=Homo sapiens OX=9606 GN=GSN PE=1 SV=1	31	12	35	11	767	84.7	5.83	104.9	12
A0A0B4J1R6	Transketolase OS=Homo sapiens OX=9606 GN=TKT PE=1 SV=1	28	6	10	6	457	49.9	7.91	26.09	6

Accession	Description	Coverage [%][a]	# Peptides[b]	# PSMs[c]	# Unique Peptides[d]	# AAs[e]	MW [kDa]	Calc. pI[f]	Score Sequest HT[g]	# Peptides: Sequest HT[h]
A0A0F7G8J1	Plasminogen OS=Homo sapiens OX=9606 GN=PLG PE=2 SV=1	2	2	2	2	809	90.6	7.36	4.13	2
A0A0S2Z3G9	Actinin alpha 4 isoform 1 (Fragment) OS=Homo sapiens OX=9606 GN=ACTN4 PE=2 SV=1	30	20	36	10	911	104.8	5.44	88.43	20
A0A0S2Z4G6	Tropomyosin 1 (Alpha), isoform CRA_o (Fragment) OS=Homo sapiens OX=9606 GN=TPM1 PE=1 SV=1	12	4	10	1	284	32.7	4.74	25.94	4
A0A140T902	Tenascin-X OS=Homo sapiens OX=9606 GN=TNXB PE=1 SV=1	4	7	7	7	4222	455.9	5.2	15.09	7
A0A140VJI7	Testicular tissue protein Li 61 OS=Homo sapiens OX=9606 PE=2 SV=1	45	13	41	13	540	60.6	6.71	131.7	13
A0A146E5L3	Mesenchymal stromal cell-and fibroblast-expressing Linx paralogue OS=Homo sapiens OX=9606 GN=Meflin PE=2 SV=1	20	5	15	5	428	46	5.15	45.39	5
A0A161I202	Lactoferrin OS=Homo sapiens OX=9606 GN=LTF PE=2 SV=1	3	2	7	2	711	78.3	8.18	15.66	2
A0A172Q381	Endosialin (Fragment) OS=Homo sapiens OX=9606 GN=TEM1 PE=2 SV=1	2	1	3	1	668	71.2	5.01	8.71	1
A0A2R8Y6G6	Alpha-enolase OS=Homo sapiens OX=9606 GN=ENO1 PE=1 SV=1	46	11	26	11	434	47.3	6.99	74.13	11
A0A2Z6ATB6	Drebrin A OS=Homo sapiens OX=9606 GN=DBN1 PE=2 SV=1	3	1	1	1	695	76.3	4.51	2.25	1
A0AV88	ADAM10 protein OS=Homo sapiens OX=9606 GN=ADAM10 PE=2 SV=1	3	1	1	1	512	57.9	7.2	2.61	1
A1KY36	Cell proliferation-inducing protein 41 OS=Homo sapiens OX=9606 PE=2 SV=1	7	2	2	2	745	73.4	9.61	4.44	2
A1L4H1	Soluble scavenger receptor cysteine-rich domain-containing protein SSC5D OS=Homo sapiens OX=9606 GN=SSC5D PE=1 SV=3	2	2	2	2	1573	165.6	6.13	4.31	2
A3KC71	Nuclear envelope protein okuribin OS=Homo sapiens OX=9606 GN=okuribin PE=2 SV=1	23	17	32	17	950	108.1	9.72	81.27	17
A3KPE2	Apolipoprotein C-III OS=Homo sapiens OX=9606 GN=APOC3 PE=2 SV=1	16	1	3	1	99	10.8	5.41	6.01	1
A4D1W7	Inhibin, beta A (Activin A, activin AB alpha polypeptide) OS=Homo sapiens OX=9606 GN=INHBA PE=2 SV=1	25	6	11	6	426	47.4	8.03	29.94	6
A4D2D2	Procollagen C-endopeptidase enhancer OS=Homo sapiens OX=9606 GN=PCOLCE PE=4 SV=1	52	15	43	15	449	47.9	7.43	123.28	15
A5PLM9	Cathepsin L1 OS=Homo sapiens OX=9606 GN=CTSL1 PE=2 SV=1	4	1	2	1	333	37.5	5.45	4.21	1
A6NLN1	Polypyrimidine tract binding protein 1, isoform CRA_b OS=Homo sapiens OX=9606 GN=PTBP1 PE=1 SV=4	4	1	1	1	527	56.5	9.38	2.17	1
A6XMH5	Beta-2-microglobulin OS=Homo sapiens OX=9606 PE=2 SV=1	24	1	6	1	92	10.4	8.02	19.75	1
A6XND1	Insulin-like growth factor binding protein 3 isoform b OS=Homo sapiens OX=9606 GN=IGFBP3 PE=1 SV=1	22	3	8	3	263	29	8.46	20.19	3
A8K2H4	cDNA FLJ78235 OS=Homo sapiens OX=9606 PE=2 SV=1	29	6	14	6	339	37.8	6.3	37.39	6
A8K3K1	cDNA FLJ78096, highly similar to Homo sapiens actin, alpha, cardiac muscle (ACTC), mRNA OS=Homo sapiens OX=9606 PE=2 SV=1	17	5	24	1	377	42	5.39	62.55	5
A8K482	Aspartate aminotransferase OS=Homo sapiens OX=9606 PE=2 SV=1	3	1	3	1	430	47.5	9.23	6.74	1
A8K7Q1	cDNA FLJ77770, highly similar to Homo sapiens nucleobindin 1 (NUCB1), mRNA OS=Homo sapiens OX=9606 PE=2 SV=1	32	10	29	10	461	53.9	5.25	79.74	10
A8K8C4	cDNA FLJ77693, highly similar to Homo sapiens calpastatin (CAST), transcript variant 3, mRNA OS=Homo sapiens OX=9606 PE=2 SV=1	3	1	1	1	431	47.1	4.84	2.11	1
A8KAJ3	cDNA FLJ77823, highly similar to Homo sapiens EGF-containing fibulin-like extracellular matrix protein 1, transcript variant 3, mRNA OS=Homo sapiens OX=9606 PE=2 SV=1	41	14	72	14	493	54.6	5.14	220.24	14
A8MX94	Glutathione S-transferase P OS=Homo sapiens OX=9606 GN=GSTP1 PE=1 SV=1	22	3	4	3	174	19.5	5.97	9.97	3

Accession	Description	Coverage [%][a]	# Peptides[b]	# PSMs[c]	# Unique Peptides[d]	# AAs[e]	MW [kDa]	Calc. pI[f]	Score Sequest HT[g]	# Peptides: Sequest HT[h]
B1AKZ4	Phosphoprotein enriched in astrocytes 15, isoform CRA_a OS=Homo sapiens OX=9606 GN=PEA15 PE=2 SV=1	9	1	1	1	130	15	5.02	2.48	1
B2R4R0	Histone H4 OS=Homo sapiens OX=9606 GN=HIST1H4H PE=2 SV=1	33	3	4	3	103	11.4	11.36	10.65	3
B2R577	Protein S100 OS=Homo sapiens OX=9606 PE=2 SV=1	20	1	2	1	90	10.1	5.91	6.26	1
B2R582	cDNA, FLJ92374, highly similar to Homo sapiens C-type lectin domain family 3, member B (CLEC3B), mRNA OS=Homo sapiens OX=9606 PE=2 SV=1	29	4	10	4	202	22.5	6.04	23.84	4
B2R5M9	cDNA, FLJ92537, highly similar to Homo sapiens procollagen-lysine, 2-oxoglutarate 5-dioxygenase (lysine hydroxylase, Ehlers-Danlos syndrome type VI) (PLOD), mRNA OS=Homo sapiens OX=9606 PE=2 SV=1	8	4	6	4	727	83.5	7.02	15	4
B2R701	cDNA, FLJ93202, Homo sapiens protease inhibitor 16 (PI16), mRNA OS=Homo sapiens OX=9606 PE=2 SV=1	17	5	27	1	428	45.7	5.38	96.21	5
B2R7T8	cDNA, FLJ93598, highly similar to Homo sapiens capping protein (actin filament) muscle Z-line, beta (CAPZB), mRNA OS=Homo sapiens OX=9606 PE=2 SV=1	3	1	1	1	272	30.6	6	1.95	1
B2R950	cDNA, FLJ94213, highly similar to Homo sapiens pregnancy-zone protein (PZP), mRNA OS=Homo sapiens OX=9606 PE=2 SV=1	2	3	5	1	1482	163.8	6.38	11.54	3
B2RBS8	cDNA, FLJ95666, highly similar to Homo sapiens albumin (ALB), mRNA OS=Homo sapiens OX=9606 PE=2 SV=1	4	3	22	3	609	69.3	6.28	60.39	3
B2RCM5	cDNA, FLJ96160, highly similar to Homo sapiens EGF-containing fibulin-like extracellular matrix protein 2 (EFEMP2), mRNA OS=Homo sapiens OX=9606 PE=2 SV=1	12	4	8	4	443	49.3	4.94	18.83	4
B2RDE1	cDNA, FLJ96568, highly similar to Homo sapiens tropomyosin 3 (TPM3), mRNA OS=Homo sapiens OX=9606 PE=2 SV=1	19	5	10	3	248	29	4.75	24.34	5
B2RDW0	cDNA, FLJ96792, highly similar to Homo sapiens calmodulin 2 (phosphorylase kinase, delta) (CALM2), mRNA OS=Homo sapiens OX=9606 PE=2 SV=1	21	2	7	2	149	16.8	4.22	19.6	2
B3KQT9	Protein disulfide-isomerase OS=Homo sapiens OX=9606 PE=2 SV=1	24	7	14	7	480	54.1	7.21	36.04	7
B4DDQ2	Biglycan OS=Homo sapiens OX=9606 PE=2 SV=1	40	10	53	10	334	37.6	7.97	160.6	10
B4DE78	cDNA FLJ52141, highly similar to 14-3-3 protein gamma OS=Homo sapiens OX=9606 PE=2 SV=1	12	2	6	1	207	23.5	4.82	14.13	2
B4DID6	cDNA FLJ52545, highly similar to Dickkopf-related protein 3 OS=Homo sapiens OX=9606 PE=2 SV=1	23	5	10	5	364	39.8	4.67	23.82	5
B4DJ30	cDNA FLJ61290, highly similar to Neutral alpha-glucosidase AB OS=Homo sapiens OX=9606 PE=2 SV=1	6	4	6	4	995	112.9	6.06	15.17	4
B4DJI2	cDNA FLJ53342, highly similar to Granulins OS=Homo sapiens OX=9606 PE=2 SV=1	3	1	1	1	530	56.8	6.87	3.17	1
B4DJI4	cDNA FLJ58065, highly similar to LIM and SH3 domain protein 1 OS=Homo sapiens OX=9606 PE=2 SV=1	12	2	3	2	220	24.5	8.54	6.54	2
B4DLV7	Rab GDP dissociation inhibitor OS=Homo sapiens OX=9606 PE=2 SV=1	25	7	9	7	449	51.1	8.18	20.51	7
B4DM79	cDNA FLJ53848, highly similar to Inter-alpha-trypsin inhibitor heavy chain H2 OS=Homo sapiens OX=9606 PE=2 SV=1	7	3	9	3	543	60.7	5.35	24.84	3
B4DNG0	cDNA FLJ58142, highly similar to Olfactomedin-like protein 3 OS=Homo sapiens OX=9606 GN=OLFML3 PE=1 SV=1	17	4	10	4	345	39	6.11	27.5	4
B4DPH4	cDNA FLJ58778, highly similar to Plasminogen OS=Homo sapiens OX=9606 PE=2 SV=1	4	1	1	1	407	44.9	7.46	2.5	1
B4DPQ0	Complement C1r subcomponent OS=Homo sapiens OX=9606 GN=C1R PE=1 SV=1	43	18	60	18	719	81.8	6.37	189.44	18
B4DPW5	cDNA FLJ59374, highly similar to Homo sapiens caldesmon 1 (CALD1), transcript variant 2, mRNA OS=Homo sapiens OX=9606 PE=2 SV=1	2	1	2	1	497	57.4	8.22	3.93	1
B4DPZ5	cDNA FLJ53495, highly similar to Polymerase I and transcript release factor OS=Homo sapiens OX=9606 PE=2 SV=1	5	1	1	1	363	40.5	5.25	2.85	1
B4DRT4	cDNA FLJ51535, highly similar to Phosphatidylethanolamine-binding protein 1 OS=Homo sapiens OX=9606 PE=2 SV=1	52	4	9	4	155	17.3	6	25.34	4

Accession	Description	Coverage [%][a]	# Peptides[b]	# PSMs[c]	# Unique Peptides[d]	# AAs[e]	MW [kDa]	Calc. pI[f]	Score Sequest HT[g]	# Peptides: Sequest HT[h]
B4DU16	cDNA FLJ54550, highly similar to Homo sapiens fibronectin 1 (FN1), transcript variant 6, mRNA OS=Homo sapiens OX=9606 PE=2 SV=1	66	35	359	1	894	98.1	6.24	1160.35	35
B4DU62	cDNA FLJ54154, highly similar to EH-domain-containing protein 2 OS=Homo sapiens OX=9606 PE=2 SV=1	15	2	2	2	185	20.6	5.08	4.74	2
B4DUQ1	cDNA FLJ54552, highly similar to Heterogeneous nuclear ribonucleoprotein K OS=Homo sapiens OX=9606 PE=2 SV=1	3	1	1	1	439	48.5	5.92	2.12	1
B4DUV1	Fibulin-1 OS=Homo sapiens OX=9606 PE=2 SV=1	20	8	15	1	641	70.1	5.26	40.12	8
B4DWC4	Chloride intracellular channel protein OS=Homo sapiens OX=9606 PE=2 SV=1	11	2	2	2	245	27.8	6.38	4.56	2
B4DY85	cDNA FLJ61463, highly similar to Zyxin OS=Homo sapiens OX=9606 PE=2 SV=1	6	2	2	2	516	55.4	6.06	4.92	2
B4E324	cDNA FLJ60397, highly similar to Lysosomal protective protein OS=Homo sapiens OX=9606 PE=2 SV=1	3	1	2	1	480	54.2	6.98	5.02	1
B4E3Q1	cDNA FLJ61580, highly similar to Calsyntenin-1 OS=Homo sapiens OX=9606 PE=2 SV=1	13	8	17	8	962	107.7	4.97	41.83	8
B7Z9B8	cDNA FLJ56912, highly similar to Fibulin-2 OS=Homo sapiens OX=9606 PE=2 SV=1	8	6	8	6	1210	129.3	4.87	18.24	6
B8ZWD1	Acyl-CoA-binding protein OS=Homo sapiens OX=9606 GN=DBI PE=1 SV=1	21	1	1	1	97	11.1	8.32	2.97	1
B9A025	Lysyl oxidase homolog 3 OS=Homo sapiens OX=9606 GN=LOXL3 PE=1 SV=1	2	1	1	1	586	64.8	6.62	1.94	1
C9J8S2	Retinoic acid receptor responder protein 2 (Fragment) OS=Homo sapiens OX=9606 GN=RARRES2 PE=1 SV=1	25	2	6	2	159	17.8	10.21	14.96	2
C9JIZ6	Prosaposin OS=Homo sapiens OX=9606 GN=PSAP PE=1 SV=2	12	4	5	4	527	58.4	5.17	13.18	4
C9JZW3	Elongation factor 1-beta (Fragment) OS=Homo sapiens OX=9606 GN=EEF1B2 PE=1 SV=1	12	1	3	1	123	13.4	4.56	8.27	1
D0PNI1	Epididymis luminal protein 4 OS=Homo sapiens OX=9606 GN=YWHAZ PE=2 SV=1	29	5	13	4	245	27.7	4.79	33.53	5
D0PNI2	Lysyl oxidase OS=Homo sapiens OX=9606 GN=LOX PE=4 SV=1	8	3	4	3	417	46.9	8.09	8.91	3
D1MGQ2	Alpha-2 globin chain OS=Homo sapiens OX=9606 GN=HBA2 PE=3 SV=1	22	2	8	2	142	15.2	8.68	24.27	2
D3DT71	Collagen, type XI, alpha 1, isoform CRA_b OS=Homo sapiens OX=9606 GN=COL11A1 PE=4 SV=1	3	3	13	1	1767	176.5	5.38	37.35	3
D3DTX7	Collagen, type I, alpha 1, isoform CRA_a OS=Homo sapiens OX=9606 GN=COL1A1 PE=4 SV=1	67	33	397	2	885	84.7	6.24	1265.77	33
D3YTG3	Target of Nesh-SH3 OS=Homo sapiens OX=9606 GN=ABI3BP PE=1 SV=1	6	8	15	8	1777	195.2	9.73	32.98	8
D6RD99	Scrapie-responsive protein 1 OS=Homo sapiens OX=9606 GN=SCRG1 PE=1 SV=1	51	2	3	2	92	10.5	7.31	8.31	2
D6RF35	Vitamin D-binding protein OS=Homo sapiens OX=9606 GN=GC PE=1 SV=1	8	2	5	2	476	53	5.52	17.1	2
D9ZGF2	Collagen, type VI, alpha 3 OS=Homo sapiens OX=9606 GN=COL6A3 PE=4 SV=1	22	44	85	44	3177	343.5	6.68	223.9	44
D9ZGG2	Vitronectin OS=Homo sapiens OX=9606 GN=VTN PE=4 SV=1	3	1	3	1	478	54.3	5.8	7.18	1
E7EUF1	Ectonucleotide pyrophosphatase/phosphodiesterase family member 2 OS=Homo sapiens OX=9606 GN=ENPP2 PE=1 SV=1	14	8	17	8	884	101.5	7.53	45.94	8
E9PL57	NEDD8-MDP1 readthrough (Fragment) OS=Homo sapiens OX=9606 GN=NEDD8-MDP1 PE=4 SV=1	8	1	1	1	170	19.5	7.43	1.95	1
E9PL83	ADM OS=Homo sapiens OX=9606 GN=ADM PE=1 SV=1	20	2	4	2	138	15	10.76	11.16	2
E9PRR2	Serine protease 23 (Fragment) OS=Homo sapiens OX=9606 GN=PRSS23 PE=1 SV=1	13	1	4	1	154	16.9	8.82	10.92	1

Accession	Description	Coverage [%][a]	# Peptides[b]	# PSMs[c]	# Unique Peptides[d]	# AAs[e]	MW [kDa]	Calc. pI[f]	Score Sequest HT[g]	# Peptides: Sequest HT[h]
F5GXS0	Complement C4-B OS=Homo sapiens OX=9606 GN=C4B PE=1 SV=1	1	1	1	1	1698	187.6	7.33	1.93	1
F5H365	Protein transport protein SEC23 OS=Homo sapiens OX=9606 GN=SEC23A PE=1 SV=1	4	2	2	2	736	82.9	7.46	4.77	2
F8VR42	Dynein regulatory complex subunit 2 (Fragment) OS=Homo sapiens OX=9606 GN=CCDC65 PE=1 SV=1	3	1	1	1	373	44	9.25	2.05	1
F8VR50	Actin-related protein 2/3 complex subunit 3 (Fragment) OS=Homo sapiens OX=9606 GN=ARPC3 PE=1 SV=1	13	1	2	1	84	9.7	7.93	4.08	1
F8VSC5	SCY1-like protein 2 (Fragment) OS=Homo sapiens OX=9606 GN=SCYL2 PE=1 SV=1	3	1	1	1	681	77	7.05	1.91	1
F8W6I7	Heterogeneous nuclear ribonucleoprotein A1 OS=Homo sapiens OX=9606 GN=HNRNPA1 PE=1 SV=2	8	2	4	2	307	33.1	9.13	9.48	2
F8W809	Thioredoxin reductase 1, cytoplasmic OS=Homo sapiens OX=9606 GN=TXNRD1 PE=1 SV=1	17	5	10	5	498	54.6	6.47	26.62	5
F8W9U3	Protein ABHD14B OS=Homo sapiens OX=9606 GN=ABHD14B PE=1 SV=1	11	1	1	1	208	22.4	6.52	2.96	1
G3V279	Enhancer of rudimentary homolog OS=Homo sapiens OX=9606 GN=ERH PE=1 SV=1	17	1	3	1	71	8.2	4.7	7.21	1
G3V3E8	NPC intracellular cholesterol transporter 2 OS=Homo sapiens OX=9606 GN=NPC2 PE=1 SV=1	22	2	2	2	174	19.2	8.44	4.63	2
G3V4U0	Fibulin-5 OS=Homo sapiens OX=9606 GN=FBLN5 PE=1 SV=1	9	3	3	3	453	50.8	4.77	7.42	3
H0YEA7	N-acetylglucosamine-1-phosphotransferase subunit gamma (Fragment) OS=Homo sapiens OX=9606 GN=GNPTG PE=1 SV=8	8	1	1	1	184	20.2	9.11	3.61	1
H0YGS3	Microfibrillar-associated protein 5 (Fragment) OS=Homo sapiens OX=9606 GN=MFAP5 PE=1 SV=1	34	2	11	2	79	9.4	9.47	33.25	2
H7BZJ3	Protein disulfide-isomerase A3 (Fragment) OS=Homo sapiens OX=9606 GN=PDIA3 PE=1 SV=1	11	1	2	1	123	13.5	7.3	4.81	1
H7C0V9	Amyloid-beta A4 protein (Fragment) OS=Homo sapiens OX=9606 GN=APP PE=1 SV=1	2	1	2	1	485	55.1	4.82	5.11	1
H7C3F9	Arp2/3 complex 34 kDa subunit (Fragment) OS=Homo sapiens OX=9606 GN=ARPC2 PE=1 SV=1	7	1	2	1	165	18.6	9.41	4.53	1
I4AY87	Macrophage migration inhibitory factor (Fragment) OS=Homo sapiens OX=9606 GN=MIF PE=1 SV=1	18	2	5	2	115	12.5	7.88	11.56	2
K4RH61	Matrix metalloproteinase 14 (Membrane-inserted) OS=Homo sapiens OX=9606 GN=MMP14 PE=2 SV=1	2	1	2	1	582	65.9	7.97	4.14	1
K7ERZ3	Perilipin-3 (Fragment) OS=Homo sapiens OX=9606 GN=PLIN3 PE=1 SV=1	16	2	2	2	292	32	5.14	4.8	2
K9MS24	Spectrin beta chain OS=Homo sapiens OX=9606 GN=SPTBN1 PE=2 SV=1	1	2	3	2	2364	274.2	5.55	7.17	2
M0R1V7	Ubiquitin-60S ribosomal protein L40 (Fragment) OS=Homo sapiens OX=9606 GN=UBA52 PE=1 SV=1	25	1	1	1	63	7.1	5.36	2.62	1
O00391	Sulfhydryl oxidase 1 OS=Homo sapiens OX=9606 GN=QSOX1 PE=1 SV=3	32	17	39	17	747	82.5	8.92	112.91	17
O00462	Beta-mannosidase OS=Homo sapiens OX=9606 GN=MANBA PE=2 SV=3	1	1	1	1	879	100.8	5.52	2.24	1
O14950	Myosin regulatory light chain 12B OS=Homo sapiens OX=9606 GN=MYL12B PE=1 SV=2	22	3	5	3	172	19.8	4.84	12.54	3
O60565	Gremlin-1 OS=Homo sapiens OX=9606 GN=GREM1 PE=1 SV=1	7	1	3	1	184	20.7	9.39	6.84	1
O76076	WNT1-inducible-signaling pathway protein 2 OS=Homo sapiens OX=9606 GN=WISP2 PE=1 SV=1	7	1	1	1	250	26.8	7.88	2.03	1
P00338	L-lactate dehydrogenase A chain OS=Homo sapiens OX=9606 GN=LDHA PE=1 SV=2	31	6	14	6	332	36.7	8.27	39.25	6
P00441	Superoxide dismutase [Cu-Zn] OS=Homo sapiens OX=9606 GN=SOD1 PE=1 SV=2	45	3	8	3	154	15.9	6.13	34.28	3

Accession	Description	Coverage [%][a]	# Peptides[b]	# PSMs[c]	# Unique Peptides[d]	# AAs[e]	MW [kDa]	Calc. pI[f]	Score Sequest HT[g]	# Peptides: Sequest HT[h]
P01023	Alpha-2-macroglobulin OS=Homo sapiens OX=9606 GN=A2M PE=1 SV=3	3	4	8	2	1474	163.2	6.46	18.84	4
P01024	Complement C3 OS=Homo sapiens OX=9606 GN=C3 PE=1 SV=2	2	3	5	3	1663	187	6.4	10.15	3
P01033	Metalloproteinase inhibitor 1 OS=Homo sapiens OX=9606 GN=TIMP1 PE=1 SV=1	54	7	55	7	207	23.2	8.1	187.32	7
P01034	Cystatin-C OS=Homo sapiens OX=9606 GN=CST3 PE=1 SV=1	56	6	15	6	146	15.8	8.75	40.6	6
P02452	Collagen alpha-1(I) chain OS=Homo sapiens OX=9606 GN=COL1A1 PE=1 SV=5	59	45	462	14	1464	138.9	5.8	1476.19	45
P02461	Collagen alpha-1(III) chain OS=Homo sapiens OX=9606 GN=COL3A1 PE=1 SV=4	41	32	157	32	1466	138.5	6.61	470.42	32
P02462	Collagen alpha-1(IV) chain OS=Homo sapiens OX=9606 GN=COL4A1 PE=1 SV=4	6	5	12	5	1669	160.5	8.28	36.22	5
P02545	Prelamin-A/C OS=Homo sapiens OX=9606 GN=LMNA PE=1 SV=1	26	12	22	12	664	74.1	7.02	58.49	12
P02647	Apolipoprotein A-I OS=Homo sapiens OX=9606 GN=APOA1 PE=1 SV=1	6	1	4	1	267	30.8	5.76	10.71	1
P04083	Annexin A1 OS=Homo sapiens OX=9606 GN=ANXA1 PE=1 SV=2	16	4	7	4	346	38.7	7.02	16.01	4
P04406	Glyceraldehyde-3-phosphate dehydrogenase OS=Homo sapiens OX=9606 GN=GAPDH PE=1 SV=3	42	8	17	8	335	36	8.46	46.02	8
P04792	Heat shock protein beta-1 OS=Homo sapiens OX=9606 GN=HSPB1 PE=1 SV=2	28	3	5	3	205	22.8	6.4	10.5	3
P05387	60S acidic ribosomal protein P2 OS=Homo sapiens OX=9606 GN=RPLP2 PE=1 SV=1	17	1	1	1	115	11.7	4.54	2.51	1
P05388	60S acidic ribosomal protein P0 OS=Homo sapiens OX=9606 GN=RPLP0 PE=1 SV=1	7	1	1	1	317	34.3	5.97	1.91	1
P05997	Collagen alpha-2(V) chain OS=Homo sapiens OX=9606 GN=COL5A2 PE=1 SV=3	37	26	72	26	1499	144.8	6.46	205.56	26
P07585	Decorin OS=Homo sapiens OX=9606 GN=DCN PE=1 SV=1	41	10	63	10	359	39.7	8.54	196.21	10
P07737	Profilin-1 OS=Homo sapiens OX=9606 GN=PFN1 PE=1 SV=2	50	5	13	5	140	15	8.27	34.03	5
P07942	Laminin subunit beta-1 OS=Homo sapiens OX=9606 GN=LAMB1 PE=1 SV=2	13	15	18	15	1786	197.9	4.94	43.2	15
P07996	Thrombospondin-1 OS=Homo sapiens OX=9606 GN=THBS1 PE=1 SV=2	24	20	34	18	1170	129.3	4.94	88.74	20
P08670	Vimentin OS=Homo sapiens OX=9606 GN=VIM PE=1 SV=4	36	16	77	16	466	53.6	5.12	214.67	16
P09382	Galectin-1 OS=Homo sapiens OX=9606 GN=LGALS1 PE=1 SV=2	54	4	19	4	135	14.7	5.5	54.09	4
P09486	SPARC OS=Homo sapiens OX=9606 GN=SPARC PE=1 SV=1	56	14	200	14	303	34.6	4.84	659.54	14
P09871	Complement C1s subcomponent OS=Homo sapiens OX=9606 GN=C1S PE=1 SV=1	39	16	50	16	688	76.6	4.96	148.19	16
P10599	Thioredoxin OS=Homo sapiens OX=9606 GN=TXN PE=1 SV=3	12	1	4	1	105	11.7	4.92	10.27	1
P10909	Clusterin OS=Homo sapiens OX=9606 GN=CLU PE=1 SV=1	22	7	20	4	449	52.5	6.27	51.45	7
P11047	Laminin subunit gamma-1 OS=Homo sapiens OX=9606 GN=LAMC1 PE=1 SV=3	18	18	25	18	1609	177.5	5.12	64.28	18
P12110	Collagen alpha-2(VI) chain OS=Homo sapiens OX=9606 GN=COL6A2 PE=1 SV=4	25	15	47	8	1019	108.5	6.21	141.92	15
P13497	Bone morphogenetic protein 1 OS=Homo sapiens OX=9606 GN=BMP1 PE=1 SV=2	5	3	5	3	986	111.2	6.9	11.38	3

Accession	Description	Coverage [%][a]	# Peptides[b]	# PSMs[c]	# Unique Peptides[d]	# AAs[e]	MW [kDa]	Calc. pI[f]	Score Sequest HT[g]	# Peptides: Sequest HT[h]
P14543	Nidogen-1 OS=Homo sapiens OX=9606 GN=NID1 PE=1 SV=3	21	17	35	17	1247	136.3	5.29	88.67	17
P16870	Carboxypeptidase E OS=Homo sapiens OX=9606 GN=CPE PE=1 SV=1	10	3	3	3	476	53.1	5.14	7.06	3
P18206	Vinculin OS=Homo sapiens OX=9606 GN=VCL PE=1 SV=4	17	14	25	14	1134	123.7	5.66	58.52	14
P19022	Cadherin-2 OS=Homo sapiens OX=9606 GN=CDH2 PE=1 SV=4	7	5	7	5	906	99.7	4.81	16.75	5
P21333	Filamin-A OS=Homo sapiens OX=9606 GN=FLNA PE=1 SV=4	24	40	74	38	2647	280.6	6.06	190.97	40
P24592	Insulin-like growth factor-binding protein 6 OS=Homo sapiens OX=9606 GN=IGFBP6 PE=1 SV=1	34	3	14	3	240	25.3	7.81	46.75	3
P25787	Proteasome subunit alpha type-2 OS=Homo sapiens OX=9606 GN=PSMA2 PE=1 SV=2	6	1	3	1	234	25.9	7.43	8.08	1
P25940	Collagen alpha-3(V) chain OS=Homo sapiens OX=9606 GN=COL5A3 PE=1 SV=3	8	7	15	7	1745	172	6.87	37.52	7
P26022	Pentraxin-related protein PTX3 OS=Homo sapiens OX=9606 GN=PTX3 PE=1 SV=3	43	12	48	12	381	41.9	5.01	131.74	12
P26038	Moesin OS=Homo sapiens OX=9606 GN=MSN PE=1 SV=3	13	8	16	8	577	67.8	6.4	39.72	8
P30041	Peroxiredoxin-6 OS=Homo sapiens OX=9606 GN=PRDX6 PE=1 SV=3	24	4	5	4	224	25	6.38	11.2	4
P30044	Peroxiredoxin-5, mitochondrial OS=Homo sapiens OX=9606 GN=PRDX5 PE=1 SV=4	8	1	1	1	214	22.1	8.7	2.48	1
P30046	D-dopachrome decarboxylase OS=Homo sapiens OX=9606 GN=DDT PE=1 SV=3	12	1	2	1	118	12.7	7.3	4.08	1
P30048	Thioredoxin-dependent peroxide reductase, mitochondrial OS=Homo sapiens OX=9606 GN=PRDX3 PE=1 SV=3	10	2	4	2	256	27.7	7.78	10.79	2
P30050	60S ribosomal protein L12 OS=Homo sapiens OX=9606 GN=RPL12 PE=1 SV=1	19	2	3	2	165	17.8	9.42	6.56	2
P30530	Tyrosine-protein kinase receptor UFO OS=Homo sapiens OX=9606 GN=AXL PE=1 SV=4	2	1	1	1	894	98.3	5.39	2.28	1
P31946	14-3-3 protein beta/alpha OS=Homo sapiens OX=9606 GN=YWHAB PE=1 SV=3	9	2	4	1	246	28.1	4.83	8.63	2
P35237	Serpin B6 OS=Homo sapiens OX=9606 GN=SERPINB6 PE=1 SV=3	13	3	3	3	376	42.6	5.27	6.35	3
P35442	Thrombospondin-2 OS=Homo sapiens OX=9606 GN=THBS2 PE=1 SV=2	11	9	15	7	1172	129.9	4.83	36.61	9
P35527	Keratin, type I cytoskeletal 9 OS=Homo sapiens OX=9606 GN=KRT9 PE=1 SV=3	6	2	2	2	623	62	5.24	4.97	2
P35555	Fibrillin-1 OS=Homo sapiens OX=9606 GN=FBN1 PE=1 SV=3	19	37	86	37	2871	312	4.93	234.4	37
P36955	Pigment epithelium-derived factor OS=Homo sapiens OX=9606 GN=SERPINF1 PE=1 SV=4	39	12	39	12	418	46.3	6.38	105.4	12
P40925	Malate dehydrogenase, cytoplasmic OS=Homo sapiens OX=9606 GN=MDH1 PE=1 SV=4	13	3	5	3	334	36.4	7.36	12.85	3
P48061	Stromal cell-derived factor 1 OS=Homo sapiens OX=9606 GN=CXCL12 PE=1 SV=1	15	1	2	1	93	10.7	9.88	6.49	1
P50454	Serpin H1 OS=Homo sapiens OX=9606 GN=SERPINH1 PE=1 SV=2	41	14	32	14	418	46.4	8.69	87.01	14
P51884	Lumican OS=Homo sapiens OX=9606 GN=LUM PE=1 SV=2	35	10	66	10	338	38.4	6.61	196.31	10
P55072	Transitional endoplasmic reticulum ATPase OS=Homo sapiens OX=9606 GN=VCP PE=1 SV=4	12	4	7	4	806	89.3	5.26	21.11	4
P55083	Microfibril-associated glycoprotein 4 OS=Homo sapiens OX=9606 GN=MFAP4 PE=1 SV=2	15	2	15	2	255	28.6	5.63	47.03	2

Accession	Description	Coverage [%][a]	# Peptides[b]	# PSMs[c]	# Unique Peptides[d]	# AAs[e]	MW [kDa]	Calc. pI[f]	Score Sequest HT[g]	# Peptides: Sequest HT[h]
P55290	Cadherin-13 OS=Homo sapiens OX=9606 GN=CDH13 PE=1 SV=1	6	3	4	3	713	78.2	4.98	10.08	3
P62857	40S ribosomal protein S28 OS=Homo sapiens OX=9606 GN=RPS28 PE=1 SV=1	17	1	2	1	69	7.8	10.7	4.62	1
P62937	Peptidyl-prolyl cis-trans isomerase A OS=Homo sapiens OX=9606 GN=PPIA PE=1 SV=2	36	3	6	3	165	18	7.81	16.12	3
P62942	Peptidyl-prolyl cis-trans isomerase FKBP1A OS=Homo sapiens OX=9606 GN=FKBP1A PE=1 SV=2	26	2	5	2	108	11.9	8.16	12.55	2
P63261	Actin, cytoplasmic 2 OS=Homo sapiens OX=9606 GN=ACTG1 PE=1 SV=1	53	12	67	1	375	41.8	5.48	204.66	12
P63313	Thymosin beta-10 OS=Homo sapiens OX=9606 GN=TMSB10 PE=1 SV=2	34	1	3	1	44	5	5.36	10.47	1
P67936	Tropomyosin alpha-4 chain OS=Homo sapiens OX=9606 GN=TPM4 PE=1 SV=3	34	9	19	4	248	28.5	4.69	42.53	9
P78539	Sushi repeat-containing protein SRPX OS=Homo sapiens OX=9606 GN=SRPX PE=1 SV=1	3	1	1	1	464	51.5	8.66	1.9	1
P80303	Nucleobindin-2 OS=Homo sapiens OX=9606 GN=NUCB2 PE=1 SV=3	3	1	1	1	420	50.2	5.12	2.46	1
Q01995	Transgelin OS=Homo sapiens OX=9606 GN=TAGLN PE=1 SV=4	39	6	17	6	201	22.6	8.84	44.55	6
Q04760	Lactoylglutathione lyase OS=Homo sapiens OX=9606 GN=GLO1 PE=1 SV=4	9	1	2	1	184	20.8	5.31	7.31	1
Q05DH1	Proteasome subunit alpha type (Fragment) OS=Homo sapiens OX=9606 GN=PSMA7 PE=2 SV=1	5	1	2	1	238	26.7	8.87	4.04	1
Q06830	Peroxiredoxin-1 OS=Homo sapiens OX=9606 GN=PRDX1 PE=1 SV=1	36	5	10	5	199	22.1	8.13	23.24	5
Q07954	Prolow-density lipoprotein receptor-related protein 1 OS=Homo sapiens OX=9606 GN=LRP1 PE=1 SV=2	1	3	3	3	4544	504.3	5.39	7.78	3
Q08629	Testican-1 OS=Homo sapiens OX=9606 GN=SPOCK1 PE=1 SV=1	21	6	8	6	439	49.1	6.1	20.71	6
Q09666	Neuroblast differentiation-associated protein AHNAK OS=Homo sapiens OX=9606 GN=AHNAK PE=1 SV=2	6	13	18	13	5890	628.7	6.15	41.12	13
Q0Z944	Beta globin (Fragment) OS=Homo sapiens OX=9606 GN=HBB PE=3 SV=1	10	1	5	1	105	11.5	6.37	11.58	1
Q12841	Follistatin-related protein 1 OS=Homo sapiens OX=9606 GN=FSTL1 PE=1 SV=1	40	10	79	10	308	35	5.52	238.97	10
Q14315	Filamin-C OS=Homo sapiens OX=9606 GN=FLNC PE=1 SV=3	4	7	14	5	2725	290.8	5.97	35.06	7
Q14767	Latent-transforming growth factor beta-binding protein 2 OS=Homo sapiens OX=9606 GN=LTBP2 PE=1 SV=3	4	5	12	5	1821	194.9	5.19	29.81	5
Q15019	Septin-2 OS=Homo sapiens OX=9606 GN=SEPT2 PE=1 SV=1	6	1	1	1	361	41.5	6.6	2.7	1
Q15063-5	Isoform 5 of Periostin OS=Homo sapiens OX=9606 GN=POSTN	57	31	221	4	809	90.4	7.39	732.85	31
Q15084	Protein disulfide-isomerase A6 OS=Homo sapiens OX=9606 GN=PDIA6 PE=1 SV=1	17	5	9	5	440	48.1	5.08	21.31	5
Q15149	Plectin OS=Homo sapiens OX=9606 GN=PLEC PE=1 SV=3	2	7	10	7	4684	531.5	5.96	22.77	7
Q15293	Reticulocalbin-1 OS=Homo sapiens OX=9606 GN=RCN1 PE=1 SV=1	22	5	9	5	331	38.9	5	24.86	5
Q15582	Transforming growth factor-beta-induced protein ig-h3 OS=Homo sapiens OX=9606 GN=TGFBI PE=1 SV=1	54	21	169	21	683	74.6	7.71	536.55	21
Q16270	Insulin-like growth factor-binding protein 7 OS=Homo sapiens OX=9606 GN=IGFBP7 PE=1 SV=1	37	8	36	8	282	29.1	7.9	102.62	8
Q16658	Fascin OS=Homo sapiens OX=9606 GN=FSCN1 PE=1 SV=3	5	2	3	2	493	54.5	7.24	8.55	2

Accession	Description	Coverage [%][a]	# Peptides[b]	# PSMs[c]	# Unique Peptides[d]	# AAs[e]	MW [kDa]	Calc. pI[f]	Score Sequest HT[g]	# Peptides: Sequest HT[h]
Q16777	Histone H2A type 2-C OS=Homo sapiens OX=9606 GN=HIST2H2AC PE=1 SV=4	22	2	3	2	129	14	10.9	6.41	2
Q16778	Histone H2B type 2-E OS=Homo sapiens OX=9606 GN=HIST2H2BE PE=1 SV=3	12	1	2	1	126	13.9	10.32	5.85	1
Q2HIZ0	Thrombospondin 3 OS=Homo sapiens OX=9606 GN=THBS3 PE=2 SV=1	3	1	1	1	956	104.1	4.65	2.31	1
Q4LDE5	Sushi, von Willebrand factor type A, EGF and pentraxin domain-containing protein 1 OS=Homo sapiens OX=9606 GN=SVEP1 PE=1 SV=3	5	11	17	11	3571	389.9	5.5	38.31	11
Q53FA4	Cysteine-rich, angiogenic inducer, 61 variant (Fragment) OS=Homo sapiens OX=9606 PE=2 SV=1	12	2	3	2	381	42	8.31	9.25	2
Q53FA7	Quinone oxidoreductase PIG3 OS=Homo sapiens OX=9606 GN=TP53I3 PE=1 SV=2	4	1	2	1	332	35.5	7.17	4.73	1
Q53G71	Calreticulin variant (Fragment) OS=Homo sapiens OX=9606 PE=2 SV=1	9	2	3	2	406	46.9	4.45	7.22	2
Q53G99	Beta actin variant (Fragment) OS=Homo sapiens OX=9606 PE=2 SV=1	53	12	68	1	375	41.7	5.59	206.12	12
Q53HQ7	Elongation factor 1-alpha (Fragment) OS=Homo sapiens OX=9606 PE=2 SV=1	8	3	4	3	462	50.2	8.94	10.05	3
Q53XB4	Full-length cDNA clone CS0DF032YM23 of Fetal brain of Homo sapiens (human) OS=Homo sapiens OX=9606 GN=RAB1 PE=2 SV=1	9	1	1	1	147	16.8	9.03	2.35	1
Q59F68	CD68 antigen variant (Fragment) OS=Homo sapiens OX=9606 PE=2 SV=1	3	1	1	1	375	42.4	5.64	2.16	1
Q59GA0	Thy-1 cell surface antigen variant (Fragment) OS=Homo sapiens OX=9606 PE=2 SV=1	10	1	3	1	145	15.9	9	7.19	1
Q5M8T4	Connective tissue growth factor OS=Homo sapiens OX=9606 GN=CTGF PE=2 SV=1	22	5	9	5	349	38	7.94	22.34	5
Q5UGI6	Serine/cysteine proteinase inhibitor clade G member 1 splice variant 2 (Fragment) OS=Homo sapiens OX=9606 GN=SERPING1 PE=2 SV=1	24	7	20	7	333	37.3	8	52.25	7
Q6EMK4	Vasorin OS=Homo sapiens OX=9606 GN=VASN PE=1 SV=1	13	5	7	5	673	71.7	7.39	17.07	5
Q6FHC9	STC2 protein (Fragment) OS=Homo sapiens OX=9606 GN=STC2 PE=2 SV=1	13	2	6	2	302	33.2	7.3	19.78	2
Q6FHU2	Phosphoglycerate mutase (Fragment) OS=Homo sapiens OX=9606 GN=PGAM1 PE=2 SV=1	25	3	5	3	254	28.8	7.18	13.89	3
Q6FHW3	DF protein OS=Homo sapiens OX=9606 GN=DF PE=2 SV=1	47	7	20	7	228	24.4	7.24	62.84	7
Q6FHZ0	Malate dehydrogenase OS=Homo sapiens OX=9606 GN=MDH2 PE=2 SV=1	24	5	9	5	338	35.5	8.68	21.91	5
Q6IAW5	CALU protein OS=Homo sapiens OX=9606 GN=CALU PE=2 SV=1	41	8	21	8	315	37.1	4.64	55.95	8
Q6NUL1	Exostoses (Multiple) 2 OS=Homo sapiens OX=9606 GN=EXT2 PE=2 SV=1	5	2	2	2	718	82.1	6.55	5.34	2
Q6UXB8	Peptidase inhibitor 16 OS=Homo sapiens OX=9606 GN=PI16 PE=1 SV=1	18	6	35	2	463	49.4	5.39	118.04	6
Q6YHK3	CD109 antigen OS=Homo sapiens OX=9606 GN=CD109 PE=1 SV=2	6	6	9	6	1445	161.6	5.85	19.92	6
Q86Z22	Epididymis secretory protein Li 297 OS=Homo sapiens OX=9606 GN=HEL-S-297 PE=2 SV=1	7	1	3	1	226	23.8	9.06	9.67	1
Q8IUX7	Adipocyte enhancer-binding protein 1 OS=Homo sapiens OX=9606 GN=AEBP1 PE=1 SV=1	15	12	24	12	1158	130.8	5.11	64.6	12
Q8NBP7	Proprotein convertase subtilisin/kexin type 9 OS=Homo sapiens OX=9606 GN=PCSK9 PE=1 SV=3	6	3	6	3	692	74.2	6.61	14.33	3
Q92520	Protein FAM3C OS=Homo sapiens OX=9606 GN=FAM3C PE=1 SV=1	7	1	3	1	227	24.7	8.29	8.17	1
Q92626	Peroxidasin homolog OS=Homo sapiens OX=9606 GN=PXDN PE=1 SV=2	10	9	12	9	1479	165.2	7.17	33.22	9

Accession	Description	Coverage [%][a]	# Peptides[b]	# PSMs[c]	# Unique Peptides[d]	# AAs[e]	MW [kDa]	Calc. pI[f]	Score Sequest HT[g]	# Peptides: Sequest HT[h]
Q969H8	Myeloid-derived growth factor OS=Homo sapiens OX=9606 GN=MYDGF PE=1 SV=1	9	1	1	1	173	18.8	6.68	2.31	1
Q96AY3	Peptidyl-prolyl cis-trans isomerase FKBP10 OS=Homo sapiens OX=9606 GN=FKBP10 PE=1 SV=1	7	3	6	3	582	64.2	5.62	15.16	3
Q96HC4	PDZ and LIM domain protein 5 OS=Homo sapiens OX=9606 GN=PDLIM5 PE=1 SV=5	2	1	1	1	596	63.9	8.21	1.93	1
Q99715	Collagen alpha-1(XII) chain OS=Homo sapiens OX=9606 GN=COL12A1 PE=1 SV=2	28	58	122	58	3063	332.9	5.53	320.45	58
Q9BRK3	Matrix remodeling-associated protein 8 OS=Homo sapiens OX=9606 GN=MXRA8 PE=1 SV=1	2	1	1	1	442	49.1	7.23	2.14	1
Q9BUD6	Spondin-2 OS=Homo sapiens OX=9606 GN=SPON2 PE=1 SV=3	6	2	2	2	331	35.8	5.52	4.09	2
Q9UHI8	A disintegrin and metalloproteinase with thrombospondin motifs 1 OS=Homo sapiens OX=9606 GN=ADAMTS1 PE=1 SV=4	3	1	1	1	967	105.3	6.83	3.01	1
Q9UNN8	Endothelial protein C receptor OS=Homo sapiens OX=9606 GN=PROCR PE=1 SV=1	7	1	1	1	238	26.7	7.18	2.32	1
Q9Y240	C-type lectin domain family 11 member A OS=Homo sapiens OX=9606 GN=CLEC11A PE=1 SV=1	12	3	3	3	323	35.7	5.16	7.14	3
Q9Y490	Talin-1 OS=Homo sapiens OX=9606 GN=TLN1 PE=1 SV=3	3	5	7	5	2541	269.6	6.07	16.81	5
Q9Y4K0	Lysyl oxidase homolog 2 OS=Homo sapiens OX=9606 GN=LOXL2 PE=1 SV=1	10	5	5	5	774	86.7	6.38	13.01	5
Q9Y6C2	EMILIN-1 OS=Homo sapiens OX=9606 GN=EMILIN1 PE=1 SV=3	1	1	2	1	1016	106.6	5.17	5.29	1
V9H1C1	Gelsolin exon 4 (Fragment) OS=Homo sapiens OX=9606 PE=4 SV=1	35	2	4	1	69	8	5.78	11.09	2
V9HW74	Ubiquitin carboxyl-terminal hydrolase OS=Homo sapiens OX=9606 GN=HEL-117 PE=2 SV=1	16	2	5	2	223	24.8	5.48	12.39	2
V9HWB4	Epididymis secretory sperm binding protein Li 89n OS=Homo sapiens OX=9606 GN=HEL-S-89n PE=2 SV=1	25	13	26	12	654	72.3	5.16	66.59	13
V9HWC6	Peptidyl-prolyl cis-trans isomerase OS=Homo sapiens OX=9606 GN=HEL-S-39 PE=2 SV=1	22	4	13	4	208	22.7	9.32	34.48	4
V9HWE8	Epididymis secretory sperm binding protein Li 47e OS=Homo sapiens OX=9606 GN=HEL-S-47e PE=2 SV=1	21	4	5	4	204	23.2	5.11	11.92	4
V9HWF4	Phosphoglycerate kinase OS=Homo sapiens OX=9606 GN=HEL-S-68p PE=2 SV=1	8	2	4	2	417	44.6	8.1	10.64	2
V9HWI5	Cofilin 1 (Non-muscle), isoform CRA_b OS=Homo sapiens OX=9606 GN=HEL-S-15 PE=2 SV=1	37	4	8	4	166	18.5	8.09	20.42	4
V9HWK1	Triosephosphate isomerase OS=Homo sapiens OX=9606 GN=HEL-S-49 PE=2 SV=1	45	8	22	8	249	26.7	6.9	62.14	8
V9HWN7	Fructose-bisphosphate aldolase OS=Homo sapiens OX=9606 GN=HEL-S-87p PE=2 SV=1	35	7	15	7	364	39.4	8.09	40.37	7
V9HWP2	Epididymis luminal protein 35 OS=Homo sapiens OX=9606 GN=HEL-S-125m PE=2 SV=1	11	6	13	5	803	92.4	4.84	31.22	6

[a] Coverage is the percentage of the sequence covered by identifications (PSMs only) from the included searches. [b] # Peptides is the number of distinct peptide sequences in the protein. [c] # PSMs is number of identified peptide spectrum matches identified from all included searches, including those redundantly identified. [d] # Unique peptide is the total number of distinct peptide sequences unique to the protein group. [e] #AAs is length of the protein sequence. [f] Calc pI (isoelectric point) number is theoretically calculated isoelectric point for the protein that is, the pH at which a particular molecule carries no net electrical charge. [g] Score Sequest HT is a protein score calculated by summing the individual scores for each peptide. The higher this score, the higher the individual score of the peptide, allowing for better identification. [h] # Peptides: Sequest HT is the number of distinct peptide sequences in the identified protein.

Supplementary Table 1-4: Detailed information on commonly secreted proteins by 3 adipose tissue (AT) mesenchymal stem cells (MSCs) under inflammatory condition.

Accession	Description	Coverage [%][a]	# Peptides[b]	# PSMs[c]	# Unique Peptides[d]	# AAs[e]	MW [kDa]	Calc. pI[f]	Score Sequest HT[g]	# Peptides: Sequest HT[h]
A0A024R0A1	Macrophage colony-stimulating factor 1 OS=Homo sapiens OX=9606 GN=CSF1 PE=4 SV=1	13	6	16	6	554	60.1	5.29	51.01	6
A0A024R0A9	Stromal cell derived factor 4, isoform CRA_b OS=Homo sapiens OX=9606 GN=SDF4 PE=4 SV=1	14	4	8	4	348	39.6	5.11	21.26	4
A0A024R1U8	Insulin-like growth factor binding protein 4, isoform CRA_a OS=Homo sapiens OX=9606 GN=IGFBP4 PE=4 SV=1	21	4	22	4	258	27.9	7.15	63.31	4
A0A024R2P0	40S ribosomal protein SA OS=Homo sapiens OX=9606 GN=RPSA PE=3 SV=1	6	1	2	1	295	32.8	4.87	3.82	1
A0A024R2W4	Dystroglycan 1 (Dystrophin-associated glycoprotein 1), isoform CRA_a OS=Homo sapiens OX=9606 GN=DAG1 PE=4 SV=1	4	2	6	2	895	97.5	8.56	20.08	2
A0A024R433	Insulin-like growth factor binding protein 5, isoform CRA_a OS=Homo sapiens OX=9606 GN=IGFBP5 PE=4 SV=1	22	4	11	4	272	30.6	8.21	30.25	4
A0A024R462	Fibronectin 1, isoform CRA_n OS=Homo sapiens OX=9606 GN=FN1 PE=4 SV=1	56	74	879	39	2355	259	5.73	3071.96	74
A0A024R5Z7	Annexin OS=Homo sapiens OX=9606 GN=ANXA2 PE=3 SV=1	40	11	29	11	339	38.6	7.75	78.57	11
A0A024R694	Actinin, alpha 1, isoform CRA_a OS=Homo sapiens OX=9606 GN=ACTN1 PE=4 SV=1	24	16	36	8	892	103	5.41	90.82	16
A0A024R6R4	Matrix metallopeptidase 2 (Gelatinase A, 72kDa gelatinase, 72kDa type IV collagenase), isoform CRA_a OS=Homo sapiens OX=9606 GN=MMP2 PE=3 SV=1	54	22	178	22	660	73.8	5.47	603.44	22
A0A024R8E5	Collagen, type V, alpha 1, isoform CRA_a OS=Homo sapiens OX=9606 GN=COL5A1 PE=4 SV=1	19	20	69	18	1838	183.4	5.06	230.97	20
A0A024R8V7	TIMP metallopeptidase inhibitor 2, isoform CRA_b OS=Homo sapiens OX=9606 GN=TIMP2 PE=4 SV=1	41	5	21	5	179	20.2	7.17	71.84	5
A0A024R969	Chitinase 3-like 1 (Cartilage glycoprotein-39), isoform CRA_a OS=Homo sapiens OX=9606 GN=CHI3L1 PE=3 SV=1	33	9	24	9	383	42.6	8.46	70.19	9
A0A024RA94	Microfibrillar-associated protein 2, isoform CRA_d OS=Homo sapiens OX=9606 GN=MFAP2 PE=4 SV=1	10	1	4	1	183	20.8	4.97	10.71	1
A0A024RAQ9	Chondroitin sulfate proteoglycan 2 (Versican), isoform CRA_b OS=Homo sapiens OX=9606 GN=CSPG2 PE=4 SV=1	5	13	37	13	3396	372.6	4.51	104.1	13
A0A024RDA5	Multifunctional fusion protein OS=Homo sapiens OX=9606 GN=IL8 PE=3 SV=1	16	1	18	1	99	11.1	8.84	57.56	1
A0A024RDW8	Collagen, type IV, alpha 2, isoform CRA_a OS=Homo sapiens OX=9606 GN=COL4A2 PE=4 SV=1	24	22	58	22	1712	167.4	8.66	175.93	22
A0A087WTA8	Collagen alpha-2(I) chain OS=Homo sapiens OX=9606 GN=COL1A2 PE=1 SV=1	61	42	1049	42	1364	129.1	9.01	3618.4	42
A0A087WX80	Laminin subunit alpha-2 OS=Homo sapiens OX=9606 GN=LAMA2 PE=1 SV=1	9	19	33	19	3121	343.5	6.39	78.76	19
A0A087WY35	Integrin beta-like protein 1 OS=Homo sapiens OX=9606 GN=ITGBL1 PE=1 SV=1	11	3	7	3	445	48.5	5.4	17.5	3
A0A087WZ51	Tumor protein D54 OS=Homo sapiens OX=9606 GN=TPD52L2 PE=1 SV=1	13	1	1	1	152	16.3	4.92	1.94	1
A0A087X0S5	Collagen alpha-1(VI) chain OS=Homo sapiens OX=9606 GN=COL6A1 PE=1 SV=1	46	27	216	27	1026	108.3	5.43	698.07	27
A0A0A0MT01	Gelsolin OS=Homo sapiens OX=9606 GN=GSN PE=1 SV=1	29	11	29	10	767	84.7	5.83	82.9	11
A0A0B4U5E3	Granulocyte-colony stimulating factor (Fragment) OS=Homo sapiens OX=9606 PE=2 SV=1	9	1	4	1	174	18.7	6.04	15.72	1
A0A0S2Z3G9	Actinin alpha 4 isoform 1 (Fragment) OS=Homo sapiens OX=9606 GN=ACTN4 PE=2 SV=1	28	19	38	11	911	104.8	5.44	92.04	19
A0A0S2Z3Y1	Lectin galactoside-binding soluble 3 binding protein isoform 1 (Fragment) OS=Homo sapiens OX=9606 GN=LGALS3BP PE=2 SV=1	22	8	17	8	585	65.3	5.27	46.43	8
A0A140T902	Tenascin-X OS=Homo sapiens OX=9606 GN=TNXB PE=1 SV=1	3	8	9	8	4222	455.9	5.2	19.14	8

Accession	Description	Coverage [%][a]	# Peptides[b]	# PSMs[c]	# Unique Peptides[d]	# AAs[e]	MW [kDa]	Calc. pI[f]	Score Sequest HT[g]	# Peptides: Sequest HT[h]
A0A140VJI7	Testicular tissue protein Li 61 OS=Homo sapiens OX=9606 PE=2 SV=1	56	18	64	13	540	60.6	6.71	215.36	18
A0A161I202	Lactoferrin OS=Homo sapiens OX=9606 GN=LTF PE=2 SV=1	3	2	6	2	711	78.3	8.18	14.35	2
A0A172Q381	Endosialin (Fragment) OS=Homo sapiens OX=9606 GN=TEM1 PE=2 SV=1	7	2	3	2	668	71.2	5.01	8.66	2
A0A1B0GU92	Uncharacterized protein OS=Homo sapiens OX=9606 PE=1 SV=1	5	2	6	2	557	59.8	6.62	14.56	2
A0A1U9X7H4	CFB OS=Homo sapiens OX=9606 PE=3 SV=1	27	15	45	15	764	85.5	6.96	149.67	15
A0A2R8Y5V9	Tropomyosin alpha-4 chain OS=Homo sapiens OX=9606 GN=TPM4 PE=1 SV=1	17	6	21	4	248	28.6	4.7	49.94	6
A1KY36	Cell proliferation-inducing protein 41 OS=Homo sapiens OX=9606 PE=2 SV=1	4	2	3	2	745	73.4	9.61	7.08	2
A1L4H1	Soluble scavenger receptor cysteine-rich domain-containing protein SSC5D OS=Homo sapiens OX=9606 GN=SSC5D PE=1 SV=3	2	2	2	2	1573	165.6	6.13	5.16	2
A2VCQ4	PRKCSH protein (Fragment) OS=Homo sapiens OX=9606 GN=PRKCSH PE=2 SV=1	13	2	3	2	181	20.3	4.72	6.66	2
A4D1W7	Inhibin, beta A (Activin A, activin AB alpha polypeptide) OS=Homo sapiens OX=9606 GN=INHBA PE=2 SV=1	27	10	47	10	426	47.4	8.03	141.96	10
A4D2D2	Procollagen C-endopeptidase enhancer OS=Homo sapiens OX=9606 GN=PCOLCE PE=4 SV=1	37	11	30	11	449	47.9	7.43	86.73	11
A5PLM9	Cathepsin L1 OS=Homo sapiens OX=9606 GN=CTSL1 PE=2 SV=1	8	2	3	2	333	37.5	5.45	9.01	2
A6XMH5	Beta-2-microglobulin OS=Homo sapiens OX=9606 PE=2 SV=1	24	1	13	1	92	10.4	8.02	50.57	1
A6XND1	Insulin-like growth factor binding protein 3 isoform b OS=Homo sapiens OX=9606 GN=IGFBP3 PE=1 SV=1	23	4	11	4	263	29	8.46	32.62	4
A8K2H4	cDNA FLJ78235 OS=Homo sapiens OX=9606 PE=2 SV=1	21	5	13	5	339	37.8	6.3	40.91	5
A8K482	Aspartate aminotransferase OS=Homo sapiens OX=9606 PE=2 SV=1	3	1	3	1	430	47.5	9.23	7.41	1
A8K7Q1	cDNA FLJ77770, highly similar to Homo sapiens nucleobindin 1 (NUCB1), mRNA OS=Homo sapiens OX=9606 PE=2 SV=1	35	11	39	11	461	53.9	5.25	113.14	11
A8K7T4	cDNA FLJ75774, highly similar to Homo sapiens lectin, mannose-binding 2 (LMAN2), mRNA OS=Homo sapiens OX=9606 PE=2 SV=1	6	1	3	1	356	40.2	6.95	7.85	1
A8KA16	cDNA FLJ77243, highly similar to Homo sapiens steroid sensitive gene 1 (URB), transcript variant 1, mRNA OS=Homo sapiens OX=9606 PE=2 SV=1	15	11	18	11	950	108.1	9.69	45.6	11
A8KAJ3	cDNA FLJ77823, highly similar to Homo sapiens EGF-containing fibulin-like extracellular matrix protein 1, transcript variant 3, mRNA OS=Homo sapiens OX=9606 PE=2 SV=1	40	13	46	13	493	54.6	5.14	132.33	13
B2R4R0	Histone H4 OS=Homo sapiens OX=9606 GN=HIST1H4H PE=2 SV=1	21	2	6	2	103	11.4	11.36	16.57	2
B2R577	Protein S100 OS=Homo sapiens OX=9606 PE=2 SV=1	20	1	1	1	90	10.1	5.91	2.04	1
B2R5J8	C-C motif chemokine OS=Homo sapiens OX=9606 PE=2 SV=1	12	1	3	1	91	10	9.07	8.66	1
B2R5M9	cDNA, FLJ92537, highly similar to Homo sapiens procollagen-lysine, 2-oxoglutarate 5-dioxygenase (lysine hydroxylase, Ehlers-Danlos syndrome type VI) (PLOD), mRNA OS=Homo sapiens OX=9606 PE=2 SV=1	8	4	6	4	727	83.5	7.02	13.48	4
B2R701	cDNA, FLJ93202, Homo sapiens protease inhibitor 16 (PI16), mRNA OS=Homo sapiens OX=9606 PE=2 SV=1	17	4	24	1	428	45.7	5.38	82.77	4
B2RBS8	cDNA, FLJ95666, highly similar to Homo sapiens albumin (ALB), mRNA OS=Homo sapiens OX=9606 PE=2 SV=1	2	2	22	2	609	69.3	6.28	71.93	2
B2RCM5	cDNA, FLJ96160, highly similar to Homo sapiens EGF-containing fibulin-like extracellular matrix protein 2 (EFEMP2), mRNA OS=Homo sapiens OX=9606 PE=2 SV=1	21	7	16	7	443	49.3	4.94	42.87	7

Accession	Description	Coverage [%][a]	# Peptides[b]	# PSMs[c]	# Unique Peptides[d]	# AAs[e]	MW [kDa]	Calc. pI[f]	Score Sequest HT[g]	# Peptides: Sequest HT[h]
B2RDW0	cDNA, FLJ96792, highly similar to Homo sapiens calmodulin 2 (phosphorylase kinase, delta) (CALM2), mRNA OS=Homo sapiens OX=9606 PE=2 SV=1	11	1	4	1	149	16.8	4.22	11.57	1
B3KQT9	Protein disulfide-isomerase OS=Homo sapiens OX=9606 PE=2 SV=1	16	5	7	5	480	54.1	7.21	22.95	5
B3KRN4	cDNA FLJ34625 fis, clone KIDNE2015244, highly similar to Serine protease HTRA1 OS=Homo sapiens OX=9606 PE=2 SV=1	19	6	17	6	447	48.4	10.13	48.3	6
B4DDQ2	Biglycan OS=Homo sapiens OX=9606 PE=2 SV=1	51	12	79	12	334	37.6	7.97	251.57	12
B4DE78	cDNA FLJ52141, highly similar to 14-3-3 protein gamma OS=Homo sapiens OX=9606 PE=2 SV=1	12	2	4	1	207	23.5	4.82	8.72	2
B4DID6	cDNA FLJ52545, highly similar to Dickkopf-related protein 3 OS=Homo sapiens OX=9606 PE=2 SV=1	12	3	8	3	364	39.8	4.67	22.18	3
B4DJ30	cDNA FLJ61290, highly similar to Neutral alpha-glucosidase AB OS=Homo sapiens OX=9606 PE=2 SV=1	7	4	9	4	995	112.9	6.06	23.48	4
B4DLV7	Rab GDP dissociation inhibitor OS=Homo sapiens OX=9606 PE=2 SV=1	8	2	4	2	449	51.1	8.18	9.62	2
B4DM79	cDNA FLJ53848, highly similar to Inter-alpha-trypsin inhibitor heavy chain H2 OS=Homo sapiens OX=9606 PE=2 SV=1	5	2	4	2	543	60.7	5.35	10.5	2
B4DMR3	cDNA FLJ51896, highly similar to Glia-derived nexin OS=Homo sapiens OX=9606 PE=2 SV=1	36	9	41	9	334	37.1	9.52	108.58	9
B4DNG0	cDNA FLJ58142, highly similar to Olfactomedin-like protein 3 OS=Homo sapiens OX=9606 GN=OLFML3 PE=1 SV=1	29	7	22	7	345	39	6.11	54.1	7
B4DPQ0	Complement C1r subcomponent OS=Homo sapiens OX=9606 GN=C1R PE=1 SV=1	50	21	113	21	719	81.8	6.37	379.97	21
B4DRT4	cDNA FLJ51535, highly similar to Phosphatidylethanolamine-binding protein 1 OS=Homo sapiens OX=9606 PE=2 SV=1	23	2	6	2	155	17.3	6	14.91	2
B4DRV4	cDNA FLJ55667, highly similar to Secreted protein acidic and rich in cysteine OS=Homo sapiens OX=9606 PE=2 SV=1	70	13	122	1	212	24.7	5.62	375.58	13
B4DU16	cDNA FLJ54550, highly similar to Homo sapiens fibronectin 1 (FN1), transcript variant 6, mRNA OS=Homo sapiens OX=9606 PE=2 SV=1	67	35	412	1	894	98.1	6.24	1465.76	35
B4DWC4	Chloride intracellular channel protein OS=Homo sapiens OX=9606 PE=2 SV=1	6	1	1	1	245	27.8	6.38	2.31	1
B4DZ36	cDNA FLJ58441, highly similar to Attractin OS=Homo sapiens OX=9606 PE=2 SV=1	1	1	3	1	1156	129.7	6.87	6.93	1
B4E324	cDNA FLJ60397, highly similar to Lysosomal protective protein OS=Homo sapiens OX=9606 PE=2 SV=1	3	1	3	1	480	54.2	6.98	7.68	1
B4E3Q1	cDNA FLJ61580, highly similar to Calsyntenin-1 OS=Homo sapiens OX=9606 PE=2 SV=1	14	9	24	9	962	107.7	4.97	71.44	9
B4E3S0	Coronin OS=Homo sapiens OX=9606 GN=CORO1C PE=1 SV=1	3	1	1	1	369	41.6	6.73	2.41	1
B5MCZ3	Interleukin-6 OS=Homo sapiens OX=9606 GN=IL6 PE=1 SV=1	48	7	49	7	189	21.5	6.57	156.65	7
B7Z1I2	Aspartate aminotransferase OS=Homo sapiens OX=9606 PE=2 SV=1	4	1	1	1	366	41	7.18	2.03	1
B7Z9B8	cDNA FLJ56912, highly similar to Fibulin-2 OS=Homo sapiens OX=9606 PE=2 SV=1	7	5	6	5	1210	129.3	4.87	14.1	5
B7ZA74	cDNA, FLJ79088, highly similar to UV excision repair protein RAD23 homolog B OS=Homo sapiens OX=9606 PE=2 SV=1	3	1	1	1	388	40.7	4.94	1.93	1
C9JIZ6	Prosaposin OS=Homo sapiens OX=9606 GN=PSAP PE=1 SV=2	20	8	13	8	527	58.4	5.17	31.46	8
C9JPM3	Disintegrin and metalloproteinase domain-containing protein 9 OS=Homo sapiens OX=9606 GN=ADAM9 PE=1 SV=1	11	1	3	1	147	16.8	8.95	7.52	1
D0PNI1	Epididymis luminal protein 4 OS=Homo sapiens OX=9606 GN=YWHAZ PE=2 SV=1	17	3	6	2	245	27.7	4.79	15.56	3
D0PNI2	Lysyl oxidase OS=Homo sapiens OX=9606 GN=LOX PE=4 SV=1	27	8	18	8	417	46.9	8.09	46.99	8

Accession	Description	Coverage [%][a]	# Peptides[b]	# PSMs[c]	# Unique Peptides[d]	# AAs[e]	MW [kDa]	Calc. pI[f]	Score Sequest HT[g]	# Peptides: Sequest HT[h]
D1MGQ2	Alpha-2 globin chain OS=Homo sapiens OX=9606 GN=HBA2 PE=3 SV=1	17	2	4	2	142	15.2	8.68	10.18	2
D3DSM4	Collagen, type XVIII, alpha 1, isoform CRA_d OS=Homo sapiens OX=9606 GN=COL18A1 PE=4 SV=1	2	2	3	2	1336	135.4	6.47	7.54	2
D3DTX7	Collagen, type I, alpha 1, isoform CRA_a OS=Homo sapiens OX=9606 GN=COL1A1 PE=4 SV=1	67	34	314	2	885	84.7	6.24	1104.89	34
D3YTG3	Target of Nesh-SH3 OS=Homo sapiens OX=9606 GN=ABI3BP PE=1 SV=1	13	16	66	16	1777	195.2	9.73	191.98	16
D6RF35	Vitamin D-binding protein OS=Homo sapiens OX=9606 GN=GC PE=1 SV=1	8	2	6	2	476	53	5.52	17.1	2
D6RF92	C-X-C motif chemokine OS=Homo sapiens OX=9606 GN=CXCL6 PE=1 SV=1	29	4	14	3	113	11.9	9.76	33.54	4
D9ZGF2	Collagen, type VI, alpha 3 OS=Homo sapiens OX=9606 GN=COL6A3 PE=4 SV=1	22	45	117	45	3177	343.5	6.68	315.59	45
E7EUF1	Ectonucleotide pyrophosphatase/phosphodiesterase family member 2 OS=Homo sapiens OX=9606 GN=ENPP2 PE=1 SV=1	10	7	14	7	884	101.5	7.53	34.65	7
E9PL83	ADM OS=Homo sapiens OX=9606 GN=ADM PE=1 SV=1	9	1	2	1	138	15	10.76	4.11	1
F5GXS0	Complement C4-B OS=Homo sapiens OX=9606 GN=C4B PE=1 SV=1	1	1	2	1	1698	187.6	7.33	5.33	1
F8VR42	Dynein regulatory complex subunit 2 (Fragment) OS=Homo sapiens OX=9606 GN=CCDC65 PE=1 SV=1	3	1	2	1	373	44	9.25	3.84	1
F8W6I7	Heterogeneous nuclear ribonucleoprotein A1 OS=Homo sapiens OX=9606 GN=HNRNPA1 PE=1 SV=2	3	1	1	1	307	33.1	9.13	2.35	1
G3V3E8	NPC intracellular cholesterol transporter 2 OS=Homo sapiens OX=9606 GN=NPC2 PE=1 SV=1	13	1	1	1	174	19.2	8.44	3.41	1
G9K389	YWHAE/FAM22B fusion protein (Fragment) OS=Homo sapiens OX=9606 GN=YWHAE/FAM22B fusion PE=2 SV=1	12	3	7	2	384	41.3	4.97	16.18	3
H0YGS3	Microfibrillar-associated protein 5 (Fragment) OS=Homo sapiens OX=9606 GN=MFAP5 PE=1 SV=1	44	3	11	3	79	9.4	9.47	28.82	3
H7BZJ3	Protein disulfide-isomerase A3 (Fragment) OS=Homo sapiens OX=9606 GN=PDIA3 PE=1 SV=1	11	1	2	1	123	13.5	7.3	6.88	1
I4AY87	Macrophage migration inhibitory factor (Fragment) OS=Homo sapiens OX=9606 GN=MIF PE=1 SV=1	18	2	4	2	115	12.5	7.88	8.37	2
M0R0Y4	Urokinase plasminogen activator surface receptor (Fragment) OS=Homo sapiens OX=9606 GN=PLAUR PE=1 SV=1	6	1	1	1	185	20.7	6.6	2.19	1
M1VE83	Tyrosine-protein kinase receptor OS=Homo sapiens OX=9606 GN=SDC4-ROS1_S4;R34 PE=2 SV=1	2	1	3	1	643	71.6	4.81	9.08	1
O00300	Tumor necrosis factor receptor superfamily member 11B OS=Homo sapiens OX=9606 GN=TNFRSF11B PE=1 SV=3	11	3	7	3	401	46	8.29	16.57	3
O00391	Sulfhydryl oxidase 1 OS=Homo sapiens OX=9606 GN=QSOX1 PE=1 SV=3	45	24	92	24	747	82.5	8.92	308.93	24
O00468	Agrin OS=Homo sapiens OX=9606 GN=AGRN PE=1 SV=6	11	14	24	14	2068	217.2	6.39	63.48	14
O14950	Myosin regulatory light chain 12B OS=Homo sapiens OX=9606 GN=MYL12B PE=1 SV=2	23	3	8	3	172	19.8	4.84	20.13	3
O14992	HS24/P52 OS=Homo sapiens OX=9606 GN=HS24/p52 PE=2 SV=1	3	1	1	1	474	52.3	8.21	2.13	1
O15511	Actin-related protein 2/3 complex subunit 5 OS=Homo sapiens OX=9606 GN=ARPC5 PE=1 SV=3	8	1	1	1	151	16.3	5.67	2.1	1
O75462	Cytokine receptor-like factor 1 OS=Homo sapiens OX=9606 GN=CRLF1 PE=1 SV=1	3	1	2	1	422	46.3	9.11	4.15	1
P01024	Complement C3 OS=Homo sapiens OX=9606 GN=C3 PE=1 SV=2	40	46	164	46	1663	187	6.4	471.33	46
P01033	Metalloproteinase inhibitor 1 OS=Homo sapiens OX=9606 GN=TIMP1 PE=1 SV=1	54	7	99	7	207	23.2	8.1	389.41	7

Accession	Description	Coverage [%][a]	# Peptides[b]	# PSMs[c]	# Unique Peptides[d]	# AAs[e]	MW [kDa]	Calc. pI[f]	Score Sequest HT[g]	# Peptides: Sequest HT[h]
P01034	Cystatin-C OS=Homo sapiens OX=9606 GN=CST3 PE=1 SV=1	44	4	18	4	146	15.8	8.75	53.53	4
P02452	Collagen alpha-1(I) chain OS=Homo sapiens OX=9606 GN=COL1A1 PE=1 SV=5	59	46	389	14	1464	138.9	5.8	1341.49	46
P02461	Collagen alpha-1(III) chain OS=Homo sapiens OX=9606 GN=COL3A1 PE=1 SV=4	41	33	195	33	1466	138.5	6.61	635.96	33
P02462	Collagen alpha-1(IV) chain OS=Homo sapiens OX=9606 GN=COL4A1 PE=1 SV=4	7	6	15	6	1669	160.5	8.28	41.88	6
P04083	Annexin A1 OS=Homo sapiens OX=9606 GN=ANXA1 PE=1 SV=2	20	5	11	5	346	38.7	7.02	26.62	5
P05997	Collagen alpha-2(V) chain OS=Homo sapiens OX=9606 GN=COL5A2 PE=1 SV=3	44	33	101	33	1499	144.8	6.46	313.17	33
P07195	L-lactate dehydrogenase B chain OS=Homo sapiens OX=9606 GN=LDHB PE=1 SV=2	3	1	3	1	334	36.6	6.05	6.54	1
P07585	Decorin OS=Homo sapiens OX=9606 GN=DCN PE=1 SV=1	33	10	57	10	359	39.7	8.54	180.62	10
P07737	Profilin-1 OS=Homo sapiens OX=9606 GN=PFN1 PE=1 SV=2	50	5	15	5	140	15	8.27	37.99	5
P07942	Laminin subunit beta-1 OS=Homo sapiens OX=9606 GN=LAMB1 PE=1 SV=2	15	16	36	16	1786	197.9	4.94	93.12	16
P07996	Thrombospondin-1 OS=Homo sapiens OX=9606 GN=THBS1 PE=1 SV=2	37	29	93	27	1170	129.3	4.94	268.97	29
P08254	Stromelysin-1 OS=Homo sapiens OX=9606 GN=MMP3 PE=1 SV=2	9	4	8	4	477	53.9	6.16	19.11	4
P08670	Vimentin OS=Homo sapiens OX=9606 GN=VIM PE=1 SV=4	39	15	73	15	466	53.6	5.12	202.72	15
P09341	Growth-regulated alpha protein OS=Homo sapiens OX=9606 GN=CXCL1 PE=1 SV=1	37	3	47	2	107	11.3	10.43	169.12	3
P09382	Galectin-1 OS=Homo sapiens OX=9606 GN=LGALS1 PE=1 SV=2	60	5	21	5	135	14.7	5.5	58.74	5
P09486	SPARC OS=Homo sapiens OX=9606 GN=SPARC PE=1 SV=1	56	14	150	2	303	34.6	4.84	468.66	14
P09871	Complement C1s subcomponent OS=Homo sapiens OX=9606 GN=C1S PE=1 SV=1	46	22	103	22	688	76.6	4.96	333.82	22
P10599	Thioredoxin OS=Homo sapiens OX=9606 GN=TXN PE=1 SV=3	23	2	7	2	105	11.7	4.92	16.18	2
P10909	Clusterin OS=Homo sapiens OX=9606 GN=CLU PE=1 SV=1	23	7	21	7	449	52.5	6.27	60.05	7
P11047	Laminin subunit gamma-1 OS=Homo sapiens OX=9606 GN=LAMC1 PE=1 SV=3	21	21	45	21	1609	177.5	5.12	119.51	21
P12110	Collagen alpha-2(VI) chain OS=Homo sapiens OX=9606 GN=COL6A2 PE=1 SV=4	23	15	45	3	1019	108.5	6.21	131.8	15
P12110-2	Isoform 2C2A of Collagen alpha-2(VI) chain OS=Homo sapiens OX=9606 GN=COL6A2	22	13	41	1	918	97.4	5.55	114.53	13
P13500	C-C motif chemokine 2 OS=Homo sapiens OX=9606 GN=CCL2 PE=1 SV=1	11	1	9	1	99	11	9.25	23.7	1
P14543	Nidogen-1 OS=Homo sapiens OX=9606 GN=NID1 PE=1 SV=3	27	23	63	22	1247	136.3	5.29	191.86	23
P15018	Leukemia inhibitory factor OS=Homo sapiens OX=9606 GN=LIF PE=1 SV=1	6	1	2	1	202	22	9.35	4.93	1
P15291	Beta-1,4-galactosyltransferase 1 OS=Homo sapiens OX=9606 GN=B4GALT1 PE=1 SV=5	27	6	14	6	398	43.9	8.65	44.01	6
P18206	Vinculin OS=Homo sapiens OX=9606 GN=VCL PE=1 SV=4	6	5	8	5	1134	123.7	5.66	16.8	5
P19022	Cadherin-2 OS=Homo sapiens OX=9606 GN=CDH2 PE=1 SV=4	10	6	29	6	906	99.7	4.81	71.12	6

Accession	Description	Coverage [%][a]	# Peptides[b]	# PSMs[c]	# Unique Peptides[d]	# AAs[e]	MW [kDa]	Calc. pI[f]	Score Sequest HT[g]	# Peptides: Sequest HT[h]
P19875	C-X-C motif chemokine 2 OS=Homo sapiens OX=9606 GN=CXCL2 PE=1 SV=1	37	3	42	1	107	11.4	10.55	153.82	3
P19876	C-X-C motif chemokine 3 OS=Homo sapiens OX=9606 GN=CXCL3 PE=1 SV=1	38	3	45	1	107	11.3	10.37	163.16	3
P20809	Interleukin-11 OS=Homo sapiens OX=9606 GN=IL11 PE=1 SV=1	12	2	4	2	199	21.4	10.62	9.49	2
P21333	Filamin-A OS=Homo sapiens OX=9606 GN=FLNA PE=1 SV=4	15	26	49	25	2647	280.6	6.06	129.45	26
P24592	Insulin-like growth factor-binding protein 6 OS=Homo sapiens OX=9606 GN=IGFBP6 PE=1 SV=1	34	3	20	3	240	25.3	7.81	80.38	3
P25940	Collagen alpha-3(V) chain OS=Homo sapiens OX=9606 GN=COL5A3 PE=1 SV=3	3	2	2	2	1745	172	6.87	5.06	2
P26022	Pentraxin-related protein PTX3 OS=Homo sapiens OX=9606 GN=PTX3 PE=1 SV=3	47	13	66	13	381	41.9	5.01	196.45	13
P28072	Proteasome subunit beta type-6 OS=Homo sapiens OX=9606 GN=PSMB6 PE=1 SV=4	5	1	2	1	239	25.3	4.92	3.96	1
P30041	Peroxiredoxin-6 OS=Homo sapiens OX=9606 GN=PRDX6 PE=1 SV=3	19	3	5	3	224	25	6.38	11.11	3
P33908	Mannosyl-oligosaccharide 1,2-alpha-mannosidase IA OS=Homo sapiens OX=9606 GN=MAN1A1 PE=1 SV=3	14	6	19	6	653	72.9	6.47	49.28	6
P35237	Serpin B6 OS=Homo sapiens OX=9606 GN=SERPINB6 PE=1 SV=3	4	1	3	1	376	42.6	5.27	6.9	1
P35442	Thrombospondin-2 OS=Homo sapiens OX=9606 GN=THBS2 PE=1 SV=2	23	17	48	15	1172	129.9	4.83	128.18	17
P35555	Fibrillin-1 OS=Homo sapiens OX=9606 GN=FBN1 PE=1 SV=3	25	46	153	46	2871	312	4.93	410.72	46
P36955	Pigment epithelium-derived factor OS=Homo sapiens OX=9606 GN=SERPINF1 PE=1 SV=4	41	13	44	13	418	46.3	6.38	129.33	13
P48061	Stromal cell-derived factor 1 OS=Homo sapiens OX=9606 GN=CXCL12 PE=1 SV=1	15	1	3	1	93	10.7	9.88	10.15	1
P48307	Tissue factor pathway inhibitor 2 OS=Homo sapiens OX=9606 GN=TFPI2 PE=1 SV=1	19	3	8	3	235	26.9	8.53	20.33	3
P51884	Lumican OS=Homo sapiens OX=9606 GN=LUM PE=1 SV=2	38	11	82	11	338	38.4	6.61	253.32	11
P55058	Phospholipid transfer protein OS=Homo sapiens OX=9606 GN=PLTP PE=1 SV=1	25	9	27	9	493	54.7	7.01	85.47	9
P55083	Microfibril-associated glycoprotein 4 OS=Homo sapiens OX=9606 GN=MFAP4 PE=1 SV=2	17	3	13	3	255	28.6	5.63	47.93	3
P55290	Cadherin-13 OS=Homo sapiens OX=9606 GN=CDH13 PE=1 SV=1	4	2	6	2	713	78.2	4.98	18.77	2
P62328	Thymosin beta-4 OS=Homo sapiens OX=9606 GN=TMSB4X PE=1 SV=2	34	2	2	2	44	5.1	5.06	4.42	2
P62857	40S ribosomal protein S28 OS=Homo sapiens OX=9606 GN=RPS28 PE=1 SV=1	17	1	1	1	69	7.8	10.7	2.33	1
P62942	Peptidyl-prolyl cis-trans isomerase FKBP1A OS=Homo sapiens OX=9606 GN=FKBP1A PE=1 SV=2	26	2	4	2	108	11.9	8.16	8.87	2
P63313	Thymosin beta-10 OS=Homo sapiens OX=9606 GN=TMSB10 PE=1 SV=2	34	1	3	1	44	5	5.36	9.69	1
P80303	Nucleobindin-2 OS=Homo sapiens OX=9606 GN=NUCB2 PE=1 SV=3	6	2	4	2	420	50.2	5.12	11.21	2
P98066	Tumor necrosis factor-inducible gene 6 protein OS=Homo sapiens OX=9606 GN=TNFAIP6 PE=1 SV=2	31	6	23	6	277	31.2	6.79	64.39	6
Q01995	Transgelin OS=Homo sapiens OX=9606 GN=TAGLN PE=1 SV=4	27	4	11	4	201	22.6	8.84	26.19	4
Q02388	Collagen alpha-1(VII) chain OS=Homo sapiens OX=9606 GN=COL7A1 PE=1 SV=2	3	5	9	5	2944	295	6.27	23.05	5

Accession	Description	Coverage [%][a]	# Peptides[b]	# PSMs[c]	# Unique Peptides[d]	# AAs[e]	MW [kDa]	Calc. pI[f]	Score Sequest HT[g]	# Peptides: Sequest HT[h]
Q04760	Lactoylglutathione lyase OS=Homo sapiens OX=9606 GN=GLO1 PE=1 SV=4	5	1	2	1	184	20.8	5.31	3.81	1
Q07092	Collagen alpha-1(XVI) chain OS=Homo sapiens OX=9606 GN=COL16A1 PE=1 SV=2	6	6	6	6	1604	157.7	7.84	14.45	6
Q07954	Prolow-density lipoprotein receptor-related protein 1 OS=Homo sapiens OX=9606 GN=LRP1 PE=1 SV=2	2	4	4	4	4544	504.3	5.39	9.96	4
Q09666	Neuroblast differentiation-associated protein AHNAK OS=Homo sapiens OX=9606 GN=AHNAK PE=1 SV=2	6	9	11	9	5890	628.7	6.15	24.72	9
Q10471	Polypeptide N-acetylgalactosaminyltransferase 2 OS=Homo sapiens OX=9606 GN=GALNT2 PE=1 SV=1	5	2	4	2	571	64.7	8.35	10.27	2
Q12841	Follistatin-related protein 1 OS=Homo sapiens OX=9606 GN=FSTL1 PE=1 SV=1	43	11	96	11	308	35	5.52	314.83	11
Q13217	DnaJ homolog subfamily C member 3 OS=Homo sapiens OX=9606 GN=DNAJC3 PE=1 SV=1	5	2	4	2	504	57.5	6.15	8.45	2
Q13219	Pappalysin-1 OS=Homo sapiens OX=9606 GN=PAPPA PE=1 SV=3	5	6	9	6	1627	180.9	6.18	21.79	6
Q14112	Nidogen-2 OS=Homo sapiens OX=9606 GN=NID2 PE=1 SV=3	5	4	7	3	1375	151.2	5.29	17.05	4
Q14315	Filamin-C OS=Homo sapiens OX=9606 GN=FLNC PE=1 SV=3	4	6	8	3	2725	290.8	5.97	19.6	6
Q14767	Latent-transforming growth factor beta-binding protein 2 OS=Homo sapiens OX=9606 GN=LTBP2 PE=1 SV=3	7	8	13	8	1821	194.9	5.19	33.02	8
Q15063-5	Isoform 5 of Periostin OS=Homo sapiens OX=9606 GN=POSTN	57	28	140	4	809	90.4	7.39	464.35	28
Q15084	Protein disulfide-isomerase A6 OS=Homo sapiens OX=9606 GN=PDIA6 PE=1 SV=1	6	2	2	2	440	48.1	5.08	4.19	2
Q15582	Transforming growth factor-beta-induced protein ig-h3 OS=Homo sapiens OX=9606 GN=TGFBI PE=1 SV=1	48	19	138	19	683	74.6	7.71	454	19
Q16270	Insulin-like growth factor-binding protein 7 OS=Homo sapiens OX=9606 GN=IGFBP7 PE=1 SV=1	38	9	57	9	282	29.1	7.9	178.9	9
Q16394	Exostosin-1 OS=Homo sapiens OX=9606 GN=EXT1 PE=1 SV=2	9	5	8	5	746	86.2	9.04	19.19	5
Q16658	Fascin OS=Homo sapiens OX=9606 GN=FSCN1 PE=1 SV=3	6	2	3	2	493	54.5	7.24	9.48	2
Q16706	Alpha-mannosidase 2 OS=Homo sapiens OX=9606 GN=MAN2A1 PE=1 SV=2	2	2	4	2	1144	131.1	7.58	9.74	2
Q16769	Glutaminyl-peptide cyclotransferase OS=Homo sapiens OX=9606 GN=QPCT PE=1 SV=1	4	1	2	1	361	40.9	6.61	4.47	1
Q16777	Histone H2A type 2-C OS=Homo sapiens OX=9606 GN=HIST2H2AC PE=1 SV=4	15	1	1	1	129	14	10.9	2.16	1
Q16778	Histone H2B type 2-E OS=Homo sapiens OX=9606 GN=HIST2H2BE PE=1 SV=3	12	1	3	1	126	13.9	10.32	9.67	1
Q2TSD0	Glyceraldehyde-3-phosphate dehydrogenase OS=Homo sapiens OX=9606 PE=2 SV=1	17	4	9	4	335	36	8.46	23.97	4
Q4LDE5	Sushi, von Willebrand factor type A, EGF and pentraxin domain-containing protein 1 OS=Homo sapiens OX=9606 GN=SVEP1 PE=1 SV=3	1	1	1	1	3571	389.9	5.5	2.71	1
Q53FA4	Cysteine-rich, angiogenic inducer, 61 variant (Fragment) OS=Homo sapiens OX=9606 PE=2 SV=1	15	3	6	3	381	42	8.31	16.05	3
Q53FR6	Cartilage oligomeric matrix protein variant (Fragment) OS=Homo sapiens OX=9606 PE=2 SV=1	17	7	15	7	757	82.7	4.61	39.94	7
Q53G71	Calreticulin variant (Fragment) OS=Homo sapiens OX=9606 PE=2 SV=1	9	2	7	2	406	46.9	4.45	18.81	2
Q53G75	Matrix metalloproteinase 1 preproprotein variant (Fragment) OS=Homo sapiens OX=9606 PE=2 SV=1	44	13	59	13	405	46.5	7.03	164.43	13
Q53G99	Beta actin variant (Fragment) OS=Homo sapiens OX=9606 PE=2 SV=1	50	11	33	1	375	41.7	5.59	94.31	11

Accession	Description	Coverage [%][a]	# Peptides[b]	# PSMs[c]	# Unique Peptides[d]	# AAs[e]	MW [kDa]	Calc. pI[f]	Score Sequest HT[g]	# Peptides: Sequest HT[h]
Q53GY0	Plastin 3 variant (Fragment) OS=Homo sapiens OX=9606 PE=2 SV=1	16	7	16	7	630	70.7	5.68	39.68	7
Q53R94	Uncharacterized protein RTN4 (Fragment) OS=Homo sapiens OX=9606 GN=RTN4 PE=4 SV=1	7	1	2	1	185	19.3	4.13	4.4	1
Q59E93	Aminopeptidase (Fragment) OS=Homo sapiens OX=9606 PE=2 SV=1	5	4	6	4	977	110.5	5.53	14.23	4
Q59GA0	Thy-1 cell surface antigen variant (Fragment) OS=Homo sapiens OX=9606 PE=2 SV=1	10	1	2	1	145	15.9	9	4.22	1
Q5M8T4	Connective tissue growth factor OS=Homo sapiens OX=9606 GN=CTGF PE=2 SV=1	18	5	14	5	349	38	7.94	33.26	5
Q5NKV8	Intercellular adhesion molecule 1 OS=Homo sapiens OX=9606 GN=ICAM1 PE=2 SV=1	8	3	7	3	532	57.8	7.68	18.94	3
Q5T7F0	Neuropilin OS=Homo sapiens OX=9606 GN=NRP1 PE=1 SV=1	6	3	5	3	704	79	5.86	11.88	3
Q5UGI6	Serine/cysteine proteinase inhibitor clade G member 1 splice variant 2 (Fragment) OS=Homo sapiens OX=9606 GN=SERPING1 PE=2 SV=1	8	2	4	2	333	37.3	8	9.64	2
Q63HR1	Uncharacterized protein DKFZp686P17171 OS=Homo sapiens OX=9606 GN=DKFZp686P17171 PE=2 SV=1	4	1	1	1	387	42.4	8.44	3.22	1
Q641Q3	Meteorin-like protein OS=Homo sapiens OX=9606 GN=METRNL PE=2 SV=1	12	2	6	2	311	34.4	8.35	18.33	2
Q6EMK4	Vasorin OS=Homo sapiens OX=9606 GN=VASN PE=1 SV=1	13	5	14	5	673	71.7	7.39	40.85	5
Q6FHC9	STC2 protein (Fragment) OS=Homo sapiens OX=9606 GN=STC2 PE=2 SV=1	24	3	7	3	302	33.2	7.3	27.38	3
Q6FHE1	FST protein OS=Homo sapiens OX=9606 GN=FST PE=2 SV=1	22	4	10	4	317	34.8	7.65	26.69	4
Q6FHW3	DF protein OS=Homo sapiens OX=9606 GN=DF PE=2 SV=1	28	5	13	5	228	24.4	7.24	44.24	5
Q6FHZ0	Malate dehydrogenase OS=Homo sapiens OX=9606 GN=MDH2 PE=2 SV=1	4	1	2	1	338	35.5	8.68	4.69	1
Q6I9S7	C-X-C motif chemokine OS=Homo sapiens OX=9606 GN=CXCL5 PE=2 SV=1	32	3	8	2	114	12	8.88	21.94	3
Q6IAW5	CALU protein OS=Homo sapiens OX=9606 GN=CALU PE=2 SV=1	27	5	8	5	315	37.1	4.64	24.78	5
Q6UXB8	Peptidase inhibitor 16 OS=Homo sapiens OX=9606 GN=PI16 PE=1 SV=1	15	4	22	1	463	49.4	5.39	67.03	4
Q6YHK3	CD109 antigen OS=Homo sapiens OX=9606 GN=CD109 PE=1 SV=2	7	7	15	7	1445	161.6	5.85	35.32	7
Q75MU2	Uncharacterized protein WBSCR1 (Fragment) OS=Homo sapiens OX=9606 GN=WBSCR1 PE=4 SV=1	8	1	1	1	156	17.1	5.31	2.11	1
Q7Z7M9	Polypeptide N-acetylgalactosaminyltransferase 5 OS=Homo sapiens OX=9606 GN=GALNT5 PE=1 SV=1	1	1	1	1	940	106.2	9.47	2.53	1
Q86UD1	Out at first protein homolog OS=Homo sapiens OX=9606 GN=OAF PE=2 SV=1	14	2	5	2	273	30.7	6.84	12.85	2
Q86X91	Inactive tyrosine-protein kinase 7 OS=Homo sapiens OX=9606 GN=PTK7 PE=1 SV=1	3	1	2	1	459	50.4	6.65	5.03	1
Q86Z22	Epididymis secretory protein Li 297 OS=Homo sapiens OX=9606 GN=HEL-S-297 PE=2 SV=1	7	1	3	1	226	23.8	9.06	11.41	1
Q8IUX7	Adipocyte enhancer-binding protein 1 OS=Homo sapiens OX=9606 GN=AEBP1 PE=1 SV=1	13	10	24	10	1158	130.8	5.11	63.62	10
Q8NBP7	Proprotein convertase subtilisin/kexin type 9 OS=Homo sapiens OX=9606 GN=PCSK9 PE=1 SV=3	18	6	13	6	692	74.2	6.61	35.37	6
Q92520	Protein FAM3C OS=Homo sapiens OX=9606 GN=FAM3C PE=1 SV=1	26	4	11	4	227	24.7	8.29	28.26	4
Q92626	Peroxidasin homolog OS=Homo sapiens OX=9606 GN=PXDN PE=1 SV=2	19	17	33	17	1479	165.2	7.17	97.04	17

Accession	Description	Coverage [%][a]	# Peptides[b]	# PSMs[c]	# Unique Peptides[d]	# AAs[e]	MW [kDa]	Calc. pI[f]	Score Sequest HT[g]	# Peptides: Sequest HT[h]
Q92820	Gamma-glutamyl hydrolase OS=Homo sapiens OX=9606 GN=GGH PE=1 SV=2	4	1	1	1	318	35.9	7.11	1.94	1
Q96CE4	Stathmin OS=Homo sapiens OX=9606 GN=STMN1 PE=1 SV=1	9	1	3	1	149	17.3	5.97	7.77	1
Q99715	Collagen alpha-1(XII) chain OS=Homo sapiens OX=9606 GN=COL12A1 PE=1 SV=2	35	68	209	68	3063	332.9	5.53	587.83	68
Q9BUD6	Spondin-2 OS=Homo sapiens OX=9606 GN=SPON2 PE=1 SV=3	13	3	5	3	331	35.8	5.52	11.08	3
V9HW74	Ubiquitin carboxyl-terminal hydrolase OS=Homo sapiens OX=9606 GN=HEL-117 PE=2 SV=1	16	2	5	2	223	24.8	5.48	15.46	2
V9HWB4	Epididymis secretory sperm binding protein Li 89n OS=Homo sapiens OX=9606 GN=HEL-S-89n PE=2 SV=1	18	8	20	7	654	72.3	5.16	53.41	8
V9HWC6	Peptidyl-prolyl cis-trans isomerase OS=Homo sapiens OX=9606 GN=HEL-S-39 PE=2 SV=1	28	5	13	5	208	22.7	9.32	32.09	5
V9HWF4	Phosphoglycerate kinase OS=Homo sapiens OX=9606 GN=HEL-S-68p PE=2 SV=1	4	1	2	1	417	44.6	8.1	5.43	1
V9HWI5	Cofilin 1 (Non-muscle), isoform CRA_b OS=Homo sapiens OX=9606 GN=HEL-S-15 PE=2 SV=1	8	1	2	1	166	18.5	8.09	4.63	1
V9HWK1	Triosephosphate isomerase OS=Homo sapiens OX=9606 GN=HEL-S-49 PE=2 SV=1	32	5	14	5	249	26.7	6.9	38.02	5
V9HWN7	Fructose-bisphosphate aldolase OS=Homo sapiens OX=9606 GN=HEL-S-87p PE=2 SV=1	30	6	15	5	364	39.4	8.09	42.41	6
V9HWP2	Epididymis luminal protein 35 OS=Homo sapiens OX=9606 GN=HEL-S-125m PE=2 SV=1	5	3	4	2	803	92.4	4.84	9.04	3

[a] Coverage is the percentage of the sequence covered by identifications (PSMs only) from the included searches. [b] # Peptides is the number of distinct peptide sequences in the protein. [c] # PSMs is number of identified peptide spectrum matches identified from all included searches, including those redundantly identified. [d] # Unique peptide is the total number of distinct peptide sequences unique to the protein group. [e] #AAs is length of the protein sequence. [f] Calc pI (isoelectric point) number is theoretically calculated isoelectric point for the protein that is, the pH at which a particular molecule carries no net electrical charge. [g] Score Sequest HT is a protein score calculated by summing the individual scores for each peptide. The higher this score, the higher the individual score of the peptide, allowing for better identification. [h] # Peptides: Sequest HT is the number of distinct peptide sequences in the identified protein.

Supplementary Table 1-5: Detailed information on commonly secreted proteins by 3 synovial membrane (SM) mesenchymal stem cells (MSCs) under normal condition.

Accession	Description	Coverage [%][a]	# Peptides[b]	# PSMs[c]	# Unique Peptides[d]	# AAs[e]	MW [kDa]	Calc. pI[f]	Score Sequest HT[g]	# Peptides: Sequest HT[h]
A0A024QYT5	Serpin peptidase inhibitor, clade E (Nexin, plasminogen activator inhibitor type 1), member 1, isoform CRA_b OS=Homo sapiens OX=9606 GN=SERPINE1 PE=3 SV=1	29	9	17	9	402	45	7.2	42.88	9
A0A024R1U8	Insulin-like growth factor binding protein 4, isoform CRA_a OS=Homo sapiens OX=9606 GN=IGFBP4 PE=4 SV=1	29	4	25	4	258	27.9	7.15	68.59	4
A0A024R2W4	Dystroglycan 1 (Dystrophin-associated glycoprotein 1), isoform CRA_a OS=Homo sapiens OX=9606 GN=DAG1 PE=4 SV=1	2	1	3	1	895	97.5	8.56	8.99	1
A0A024R319	Laminin, beta 2 (Laminin S), isoform CRA_a OS=Homo sapiens OX=9606 GN=LAMB2 PE=4 SV=1	7	6	6	6	1798	195.9	6.52	10.83	6
A0A024R433	Insulin-like growth factor binding protein 5, isoform CRA_a OS=Homo sapiens OX=9606 GN=IGFBP5 PE=4 SV=1	27	5	41	5	272	30.6	8.21	114.04	5
A0A024R462	Fibronectin 1, isoform CRA_n OS=Homo sapiens OX=9606 GN=FN1 PE=4 SV=1	55	68	413	3	2355	259	5.73	1234.77	68
A0A024R498	Serpin peptidase inhibitor, clade E (Nexin, plasminogen activator inhibitor type 1), member 2, isoform CRA_b OS=Homo sapiens OX=9606 GN=SERPINE2 PE=3 SV=1	26	6	15	6	397	44	9.38	39.53	6
A0A024R6R4	Matrix metallopeptidase 2 (Gelatinase A, 72kDa gelatinase, 72kDa type IV collagenase), isoform CRA_a OS=Homo sapiens OX=9606 GN=MMP2 PE=3 SV=1	47	18	59	18	660	73.8	5.47	163.2	18
A0A024R8E5	Collagen, type V, alpha 1, isoform CRA_a OS=Homo sapiens OX=9606 GN=COL5A1 PE=4 SV=1	7	6	18	6	1838	183.4	5.06	51.67	6
A0A024R8V7	TIMP metallopeptidase inhibitor 2, isoform CRA_b OS=Homo sapiens OX=9606 GN=TIMP2 PE=4 SV=1	37	4	22	4	179	20.2	7.17	60.63	4
A0A024R962	HCG40889, isoform CRA_b OS=Homo sapiens OX=9606 GN=hCG_40889 PE=4 SV=1	3	2	2	2	1231	139	6.62	4.93	2
A0A024R969	Chitinase 3-like 1 (Cartilage glycoprotein-39), isoform CRA_a OS=Homo sapiens OX=9606 GN=CHI3L1 PE=3 SV=1	40	11	31	11	383	42.6	8.46	80.05	11
A0A024RDW8	Collagen, type IV, alpha 2, isoform CRA_a OS=Homo sapiens OX=9606 GN=COL4A2 PE=4 SV=1	8	7	11	7	1712	167.4	8.66	26.63	7
A0A087WTA8	Collagen alpha-2(I) chain OS=Homo sapiens OX=9606 GN=COL1A2 PE=1 SV=1	57	44	486	44	1364	129.1	9.01	1443.37	44
A0A087WY35	Integrin beta-like protein 1 OS=Homo sapiens OX=9606 GN=ITGBL1 PE=1 SV=1	7	1	1	1	445	48.5	5.4	2.71	1
A0A087X0S5	Collagen alpha-1(VI) chain OS=Homo sapiens OX=9606 GN=COL6A1 PE=1 SV=1	38	21	106	21	1026	108.3	5.43	308.92	21
A0A0A0MT01	Gelsolin OS=Homo sapiens OX=9606 GN=GSN PE=1 SV=1	10	4	8	4	767	84.7	5.83	19.96	4
A0A0F7G8J1	Plasminogen OS=Homo sapiens OX=9606 GN=PLG PE=2 SV=1	1	1	3	1	809	90.6	7.36	7.17	1
A0A140VJI7	Testicular tissue protein Li 61 OS=Homo sapiens OX=9606 PE=2 SV=1	11	4	6	4	540	60.6	6.71	16.86	4
A0A161I202	Lactoferrin OS=Homo sapiens OX=9606 GN=LTF PE=2 SV=1	3	2	6	2	711	78.3	8.18	12.53	2
A0A172Q381	Endosialin (Fragment) OS=Homo sapiens OX=9606 GN=TEM1 PE=2 SV=1	4	2	2	2	668	71.2	5.01	4.2	2
A0AV88	ADAM10 protein OS=Homo sapiens OX=9606 GN=ADAM10 PE=2 SV=1	3	1	1	1	512	57.9	7.2	2.63	1
A1L4H1	Soluble scavenger receptor cysteine-rich domain-containing protein SSC5D OS=Homo sapiens OX=9606 GN=SSC5D PE=1 SV=3	1	1	2	1	1573	165.6	6.13	4.36	1
A4D2D2	Procollagen C-endopeptidase enhancer OS=Homo sapiens OX=9606 GN=PCOLCE PE=4 SV=1	27	9	21	9	449	47.9	7.43	52.52	9
A6XND1	Insulin-like growth factor binding protein 3 isoform b OS=Homo sapiens OX=9606 GN=IGFBP3 PE=1 SV=1	30	5	15	5	263	29	8.46	38.71	5
A8K2H4	cDNA FLJ78235 OS=Homo sapiens OX=9606 PE=2 SV=1	17	4	5	4	339	37.8	6.3	13.52	4
A8K7Q1	cDNA FLJ77770, highly similar to Homo sapiens nucleobindin 1 (NUCB1), mRNA OS=Homo sapiens OX=9606 PE=2 SV=1	23	6	14	6	461	53.9	5.25	40.81	6

Accession	Description	Coverage [%][a]	# Peptides[b]	# PSMs[c]	# Unique Peptides[d]	# AAs[e]	MW [kDa]	Calc. pI[f]	Score Sequest HT[g]	# Peptides: Sequest HT[h]
A8KAJ3	cDNA FLJ77823, highly similar to Homo sapiens EGF-containing fibulin-like extracellular matrix protein 1, transcript variant 3, mRNA OS=Homo sapiens OX=9606 PE=2 SV=1	46	16	70	16	493	54.6	5.14	196.55	16
B2R582	cDNA, FLJ92374, highly similar to Homo sapiens C-type lectin domain family 3, member B (CLEC3B), mRNA OS=Homo sapiens OX=9606 PE=2 SV=1	49	7	19	7	202	22.5	6.04	46.94	7
B2R5M9	cDNA, FLJ92537, highly similar to Homo sapiens procollagen-lysine, 2-oxoglutarate 5-dioxygenase (lysine hydroxylase, Ehlers-Danlos syndrome type VI) (PLOD), mRNA OS=Homo sapiens OX=9606 PE=2 SV=1	3	2	4	2	727	83.5	7.02	8.59	2
B2R950	cDNA, FLJ94213, highly similar to Homo sapiens pregnancy-zone protein (PZP), mRNA OS=Homo sapiens OX=9606 PE=2 SV=1	1	2	3	1	1482	163.8	6.38	6.99	2
B2RBS8	cDNA, FLJ95666, highly similar to Homo sapiens albumin (ALB), mRNA OS=Homo sapiens OX=9606 PE=2 SV=1	4	3	25	3	609	69.3	6.28	59.38	3
B2RCM5	cDNA, FLJ96160, highly similar to Homo sapiens EGF-containing fibulin-like extracellular matrix protein 2 (EFEMP2), mRNA OS=Homo sapiens OX=9606 PE=2 SV=1	6	2	2	2	443	49.3	4.94	3.93	2
B2RDW0	cDNA, FLJ96792, highly similar to Homo sapiens calmodulin 2 (phosphorylase kinase, delta) (CALM2), mRNA OS=Homo sapiens OX=9606 PE=2 SV=1	21	2	4	2	149	16.8	4.22	12.03	2
B4DDQ2	Biglycan OS=Homo sapiens OX=9606 PE=2 SV=1	34	9	38	9	334	37.6	7.97	107.39	9
B4DID6	cDNA FLJ52545, highly similar to Dickkopf-related protein 3 OS=Homo sapiens OX=9606 PE=2 SV=1	11	2	2	2	364	39.8	4.67	4.72	2
B4DNG0	cDNA FLJ58142, highly similar to Olfactomedin-like protein 3 OS=Homo sapiens OX=9606 GN=OLFML3 PE=1 SV=1	9	2	3	2	345	39	6.11	7.29	2
B4DPH4	cDNA FLJ58778, highly similar to Plasminogen OS=Homo sapiens OX=9606 PE=2 SV=1	4	1	1	1	407	44.9	7.46	2.51	1
B4DPN0	cDNA FLJ51265, moderately similar to Beta-2-glycoprotein 1 (Beta-2-glycoprotein I) OS=Homo sapiens OX=9606 PE=2 SV=1	3	1	1	1	274	30.3	7.85	1.9	1
B4DPQ0	Complement C1r subcomponent OS=Homo sapiens OX=9606 GN=C1R PE=1 SV=1	22	9	25	9	719	81.8	6.37	77.12	9
B4DPZ5	cDNA FLJ53495, highly similar to Polymerase I and transcript release factor OS=Homo sapiens OX=9606 PE=2 SV=1	8	2	2	2	363	40.5	5.25	5.08	2
B4DRT4	cDNA FLJ51535, highly similar to Phosphatidylethanolamine-binding protein 1 OS=Homo sapiens OX=9606 PE=2 SV=1	22	2	2	2	155	17.3	6	4.13	2
B4DU16	cDNA FLJ54550, highly similar to Homo sapiens fibronectin 1 (FN1), transcript variant 6, mRNA OS=Homo sapiens OX=9606 PE=2 SV=1	61	28	182	1	894	98.1	6.24	543.36	28
B4E324	cDNA FLJ60397, highly similar to Lysosomal protective protein OS=Homo sapiens OX=9606 PE=2 SV=1	3	1	1	1	480	54.2	6.98	2.7	1
B4E3Q1	cDNA FLJ61580, highly similar to Calsyntenin-1 OS=Homo sapiens OX=9606 PE=2 SV=1	9	5	5	5	962	107.7	4.97	12.13	5
D0PNI2	Lysyl oxidase OS=Homo sapiens OX=9606 GN=LOX PE=4 SV=1	3	1	1	1	417	46.9	8.09	1.94	1
D1MGQ2	Alpha-2 globin chain OS=Homo sapiens OX=9606 GN=HBA2 PE=3 SV=1	22	2	5	2	142	15.2	8.68	14.81	2
D3DNU8	Kininogen 1, isoform CRA_a OS=Homo sapiens OX=9606 GN=KNG1 PE=4 SV=1	2	1	2	1	427	47.8	6.65	4.19	1
D3DTX7	Collagen, type I, alpha 1, isoform CRA_a OS=Homo sapiens OX=9606 GN=COL1A1 PE=4 SV=1	63	31	212	2	885	84.7	6.24	622.06	31
D3YTG3	Target of Nesh-SH3 OS=Homo sapiens OX=9606 GN=ABI3BP PE=1 SV=1	4	5	9	5	1777	195.2	9.73	22.08	5
D6RD99	Scrapie-responsive protein 1 OS=Homo sapiens OX=9606 GN=SCRG1 PE=1 SV=1	20	1	1	1	92	10.5	7.31	2.25	1
D6RF35	Vitamin D-binding protein OS=Homo sapiens OX=9606 GN=GC PE=1 SV=1	8	2	7	2	476	53	5.52	22.05	2
D9ZGG2	Vitronectin OS=Homo sapiens OX=9606 GN=VTN PE=4 SV=1	3	1	1	1	478	54.3	5.8	2.54	1
E5RJA8	Carboxypeptidase Q (Fragment) OS=Homo sapiens OX=9606 GN=CPQ PE=1 SV=1	6	1	1	1	186	20.7	8.56	1.91	1

Accession	Description	Coverage [%][a]	# Peptides[b]	# PSMs[c]	# Unique Peptides[d]	# AAs[e]	MW [kDa]	Calc. pI[f]	Score Sequest HT[g]	# Peptides: Sequest HT[h]
E7EUF1	Ectonucleotide pyrophosphatase/phosphodiesterase family member 2 OS=Homo sapiens OX=9606 GN=ENPP2 PE=1 SV=1	5	3	5	3	884	101.5	7.53	12.94	3
E9PL83	ADM OS=Homo sapiens OX=9606 GN=ADM PE=1 SV=1	10	1	1	1	138	15	10.76	2.22	1
F8VR42	Dynein regulatory complex subunit 2 (Fragment) OS=Homo sapiens OX=9606 GN=CCDC65 PE=1 SV=1	3	1	5	1	373	44	9.25	9.76	1
F8W6I7	Heterogeneous nuclear ribonucleoprotein A1 OS=Homo sapiens OX=9606 GN=HNRNPA1 PE=1 SV=2	3	1	3	1	307	33.1	9.13	6.57	1
H0YGS3	Microfibrillar-associated protein 5 (Fragment) OS=Homo sapiens OX=9606 GN=MFAP5 PE=1 SV=1	34	2	6	2	79	9.4	9.47	16.69	2
H6VRF8	Keratin 1 OS=Homo sapiens OX=9606 GN=KRT1 PE=3 SV=1	11	6	10	5	644	66	8.12	21.4	6
H7BZJ3	Protein disulfide-isomerase A3 (Fragment) OS=Homo sapiens OX=9606 GN=PDIA3 PE=1 SV=1	20	2	2	1	123	13.5	7.3	4.03	2
H7C0V9	Amyloid-beta A4 protein (Fragment) OS=Homo sapiens OX=9606 GN=APP PE=1 SV=1	2	1	2	1	485	55.1	4.82	4.82	1
I4AY87	Macrophage migration inhibitory factor (Fragment) OS=Homo sapiens OX=9606 GN=MIF PE=1 SV=1	8	1	2	1	115	12.5	7.88	4.33	1
O00391	Sulfhydryl oxidase 1 OS=Homo sapiens OX=9606 GN=QSOX1 PE=1 SV=3	21	10	22	10	747	82.5	8.92	56.21	10
O76076	WNT1-inducible-signaling pathway protein 2 OS=Homo sapiens OX=9606 GN=WISP2 PE=1 SV=1	7	1	2	1	250	26.8	7.88	5.16	1
P00338	L-lactate dehydrogenase A chain OS=Homo sapiens OX=9606 GN=LDHA PE=1 SV=2	8	2	4	2	332	36.7	8.27	9.09	2
P00441	Superoxide dismutase [Cu-Zn] OS=Homo sapiens OX=9606 GN=SOD1 PE=1 SV=2	16	1	2	1	154	15.9	6.13	5.53	1
P01008	Antithrombin-III OS=Homo sapiens OX=9606 GN=SERPINC1 PE=1 SV=1	4	2	3	2	464	52.6	6.71	6.97	2
P01023	Alpha-2-macroglobulin OS=Homo sapiens OX=9606 GN=A2M PE=1 SV=3	1	2	5	1	1474	163.2	6.46	10.33	2
P01024	Complement C3 OS=Homo sapiens OX=9606 GN=C3 PE=1 SV=2	1	1	1	1	1663	187	6.4	2.66	1
P01033	Metalloproteinase inhibitor 1 OS=Homo sapiens OX=9606 GN=TIMP1 PE=1 SV=1	50	6	33	6	207	23.2	8.1	112.85	6
P01034	Cystatin-C OS=Homo sapiens OX=9606 GN=CST3 PE=1 SV=1	30	3	7	3	146	15.8	8.75	16.82	3
P02452	Collagen alpha-1(I) chain OS=Homo sapiens OX=9606 GN=COL1A1 PE=1 SV=5	47	38	223	9	1464	138.9	5.8	631.24	38
P02461	Collagen alpha-1(III) chain OS=Homo sapiens OX=9606 GN=COL3A1 PE=1 SV=4	28	28	81	28	1466	138.5	6.61	209.29	28
P02647	Apolipoprotein A-I OS=Homo sapiens OX=9606 GN=APOA1 PE=1 SV=1	6	1	2	1	267	30.8	5.76	5	1
P05387	60S acidic ribosomal protein P2 OS=Homo sapiens OX=9606 GN=RPLP2 PE=1 SV=1	11	1	1	1	115	11.7	4.54	2.18	1
P05997	Collagen alpha-2(V) chain OS=Homo sapiens OX=9606 GN=COL5A2 PE=1 SV=3	15	13	26	13	1499	144.8	6.46	66.89	13
P07585	Decorin OS=Homo sapiens OX=9606 GN=DCN PE=1 SV=1	38	10	39	10	359	39.7	8.54	105.71	10
P07737	Profilin-1 OS=Homo sapiens OX=9606 GN=PFN1 PE=1 SV=2	30	3	7	3	140	15	8.27	16.32	3
P07942	Laminin subunit beta-1 OS=Homo sapiens OX=9606 GN=LAMB1 PE=1 SV=2	4	5	5	5	1786	197.9	4.94	9.74	5
P07996	Thrombospondin-1 OS=Homo sapiens OX=9606 GN=THBS1 PE=1 SV=2	20	17	33	15	1170	129.3	4.94	71.58	17
P08670	Vimentin OS=Homo sapiens OX=9606 GN=VIM PE=1 SV=4	29	13	35	13	466	53.6	5.12	84.34	13

Accession	Description	Coverage [%][a]	# Peptides[b]	# PSMs[c]	# Unique Peptides[d]	# AAs[e]	MW [kDa]	Calc. pI[f]	Score Sequest HT[g]	# Peptides: Sequest HT[h]
P08697	Alpha-2-antiplasmin OS=Homo sapiens OX=9606 GN=SERPINF2 PE=1 SV=3	2	1	3	1	491	54.5	6.29	6.87	1
P09382	Galectin-1 OS=Homo sapiens OX=9606 GN=LGALS1 PE=1 SV=2	35	3	6	3	135	14.7	5.5	14.15	3
P09486	SPARC OS=Homo sapiens OX=9606 GN=SPARC PE=1 SV=1	56	13	143	2	303	34.6	4.84	466.48	13
P09871	Complement C1s subcomponent OS=Homo sapiens OX=9606 GN=C1S PE=1 SV=1	27	12	25	12	688	76.6	4.96	61.74	12
P10599	Thioredoxin OS=Homo sapiens OX=9606 GN=TXN PE=1 SV=3	12	1	2	1	105	11.7	4.92	4.01	1
P11047	Laminin subunit gamma-1 OS=Homo sapiens OX=9606 GN=LAMC1 PE=1 SV=3	9	9	19	9	1609	177.5	5.12	46.3	9
P12110	Collagen alpha-2(VI) chain OS=Homo sapiens OX=9606 GN=COL6A2 PE=1 SV=4	17	11	25	11	1019	108.5	6.21	73.86	11
P14543	Nidogen-1 OS=Homo sapiens OX=9606 GN=NID1 PE=1 SV=3	9	7	11	7	1247	136.3	5.29	26.2	7
P18206	Vinculin OS=Homo sapiens OX=9606 GN=VCL PE=1 SV=4	3	3	4	3	1134	123.7	5.66	7.79	3
P21333	Filamin-A OS=Homo sapiens OX=9606 GN=FLNA PE=1 SV=4	7	11	14	11	2647	280.6	6.06	34.19	11
P24592	Insulin-like growth factor-binding protein 6 OS=Homo sapiens OX=9606 GN=IGFBP6 PE=1 SV=1	24	3	10	3	240	25.3	7.81	30.63	3
P26022	Pentraxin-related protein PTX3 OS=Homo sapiens OX=9606 GN=PTX3 PE=1 SV=3	39	11	42	11	381	41.9	5.01	109.86	11
P30041	Peroxiredoxin-6 OS=Homo sapiens OX=9606 GN=PRDX6 PE=1 SV=3	15	2	2	2	224	25	6.38	4.52	2
P35442	Thrombospondin-2 OS=Homo sapiens OX=9606 GN=THBS2 PE=1 SV=2	17	13	21	11	1172	129.9	4.83	54.8	13
P35527	Keratin, type I cytoskeletal 9 OS=Homo sapiens OX=9606 GN=KRT9 PE=1 SV=3	3	1	2	1	623	62	5.24	5.76	1
P35555	Fibrillin-1 OS=Homo sapiens OX=9606 GN=FBN1 PE=1 SV=3	12	25	48	25	2871	312	4.93	125.72	25
P36955	Pigment epithelium-derived factor OS=Homo sapiens OX=9606 GN=SERPINF1 PE=1 SV=4	9	4	6	4	418	46.3	6.38	17.75	4
P48061	Stromal cell-derived factor 1 OS=Homo sapiens OX=9606 GN=CXCL12 PE=1 SV=1	15	1	3	1	93	10.7	9.88	7.39	1
P50454	Serpin H1 OS=Homo sapiens OX=9606 GN=SERPINH1 PE=1 SV=2	31	10	29	10	418	46.4	8.69	72.02	10
P51884	Lumican OS=Homo sapiens OX=9606 GN=LUM PE=1 SV=2	29	7	42	7	338	38.4	6.61	113.38	7
P55083	Microfibril-associated glycoprotein 4 OS=Homo sapiens OX=9606 GN=MFAP4 PE=1 SV=2	15	2	3	2	255	28.6	5.63	6.32	2
P55290	Cadherin-13 OS=Homo sapiens OX=9606 GN=CDH13 PE=1 SV=1	2	1	3	1	713	78.2	4.98	8.11	1
P62328	Thymosin beta-4 OS=Homo sapiens OX=9606 GN=TMSB4X PE=1 SV=2	27	1	1	1	44	5.1	5.06	2.21	1
P63261	Actin, cytoplasmic 2 OS=Homo sapiens OX=9606 GN=ACTG1 PE=1 SV=1	48	10	33	1	375	41.8	5.48	82.22	10
P63313	Thymosin beta-10 OS=Homo sapiens OX=9606 GN=TMSB10 PE=1 SV=2	34	1	1	1	44	5	5.36	2.45	1
Q01995	Transgelin OS=Homo sapiens OX=9606 GN=TAGLN PE=1 SV=4	20	3	5	3	201	22.6	8.84	11.96	3
Q08629	Testican-1 OS=Homo sapiens OX=9606 GN=SPOCK1 PE=1 SV=1	18	6	9	6	439	49.1	6.1	19.97	6
Q0Z944	Beta globin (Fragment) OS=Homo sapiens OX=9606 GN=HBB PE=3 SV=1	10	1	3	1	105	11.5	6.37	6.81	1

Accession	Description	Coverage [%][a]	# Peptides[b]	# PSMs[c]	# Unique Peptides[d]	# AAs[e]	MW [kDa]	Calc. pI[f]	Score Sequest HT[g]	# Peptides: Sequest HT[h]
Q10471	Polypeptide N-acetylgalactosaminyltransferase 2 OS=Homo sapiens OX=9606 GN=GALNT2 PE=1 SV=1	4	1	1	1	571	64.7	8.35	2.17	1
Q12841	Follistatin-related protein 1 OS=Homo sapiens OX=9606 GN=FSTL1 PE=1 SV=1	40	8	35	8	308	35	5.52	99.97	8
Q14767	Latent-transforming growth factor beta-binding protein 2 OS=Homo sapiens OX=9606 GN=LTBP2 PE=1 SV=3	1	1	2	1	1821	194.9	5.19	4.44	1
Q15582	Transforming growth factor-beta-induced protein ig-h3 OS=Homo sapiens OX=9606 GN=TGFBI PE=1 SV=1	46	19	88	19	683	74.6	7.71	267.44	19
Q16270	Insulin-like growth factor-binding protein 7 OS=Homo sapiens OX=9606 GN=IGFBP7 PE=1 SV=1	33	7	28	7	282	29.1	7.9	75.23	7
Q16778	Histone H2B type 2-E OS=Homo sapiens OX=9606 GN=HIST2H2BE PE=1 SV=3	12	1	2	1	126	13.9	10.32	5.54	1
Q53FA4	Cysteine-rich, angiogenic inducer, 61 variant (Fragment) OS=Homo sapiens OX=9606 PE=2 SV=1	12	2	2	2	381	42	8.31	5.27	2
Q53G99	Beta actin variant (Fragment) OS=Homo sapiens OX=9606 PE=2 SV=1	48	10	34	1	375	41.7	5.59	84.73	10
Q59GA0	Thy-1 cell surface antigen variant (Fragment) OS=Homo sapiens OX=9606 PE=2 SV=1	10	1	1	1	145	15.9	9	2.02	1
Q5M8T4	Connective tissue growth factor OS=Homo sapiens OX=9606 GN=CTGF PE=2 SV=1	11	3	5	3	349	38	7.94	10.18	3
Q6EMK4	Vasorin OS=Homo sapiens OX=9606 GN=VASN PE=1 SV=1	11	4	5	4	673	71.7	7.39	12.17	4
Q6FHC9	STC2 protein (Fragment) OS=Homo sapiens OX=9606 GN=STC2 PE=2 SV=1	13	2	6	2	302	33.2	7.3	21.24	2
Q6FHW3	DF protein OS=Homo sapiens OX=9606 GN=DF PE=2 SV=1	24	3	6	3	228	24.4	7.24	16.93	3
Q6IAW5	CALU protein OS=Homo sapiens OX=9606 GN=CALU PE=2 SV=1	25	5	8	5	315	37.1	4.64	22.42	5
Q6YHK3	CD109 antigen OS=Homo sapiens OX=9606 GN=CD109 PE=1 SV=2	1	1	2	1	1445	161.6	5.85	4.71	1
Q8IUX7	Adipocyte enhancer-binding protein 1 OS=Homo sapiens OX=9606 GN=AEBP1 PE=1 SV=1	10	8	13	8	1158	130.8	5.11	32.22	8
Q8TB73	Protein NDNF OS=Homo sapiens OX=9606 GN=NDNF PE=1 SV=2	10	4	8	4	568	64.6	8.94	17.18	4
Q99715	Collagen alpha-1(XII) chain OS=Homo sapiens OX=9606 GN=COL12A1 PE=1 SV=2	7	17	27	17	3063	332.9	5.53	65.31	17
Q99985	Semaphorin-3C OS=Homo sapiens OX=9606 GN=SEMA3C PE=2 SV=2	1	1	1	1	751	85.2	8.69	2.15	1
Q9UHI8	A disintegrin and metalloproteinase with thrombospondin motifs 1 OS=Homo sapiens OX=9606 GN=ADAMTS1 PE=1 SV=4	3	1	1	1	967	105.3	6.83	2.92	1
V9HWC6	Peptidyl-prolyl cis-trans isomerase OS=Homo sapiens OX=9606 GN=HEL-S-39 PE=2 SV=1	28	5	12	5	208	22.7	9.32	26.88	5
V9HWE8	Epididymis secretory sperm binding protein Li 47e OS=Homo sapiens OX=9606 GN=HEL-S-47e PE=2 SV=1	7	1	1	1	204	23.2	5.11	2.25	1
V9HWI5	Cofilin 1 (Non-muscle), isoform CRA_b OS=Homo sapiens OX=9606 GN=HEL-S-15 PE=2 SV=1	20	2	4	2	166	18.5	8.09	8.95	2
V9HWK1	Triosephosphate isomerase OS=Homo sapiens OX=9606 GN=HEL-S-49 PE=2 SV=1	12	2	3	2	249	26.7	6.9	7.05	2
V9HWN7	Fructose-bisphosphate aldolase OS=Homo sapiens OX=9606 GN=HEL-S-87p PE=2 SV=1	20	5	11	5	364	39.4	8.09	28	5
X6RJP6	Transgelin-2 (Fragment) OS=Homo sapiens OX=9606 GN=TAGLN2 PE=1 SV=1	14	2	4	2	187	21.1	7.81	9.65	2

[a] Coverage is the percentage of the sequence covered by identifications (PSMs only) from the included searches. [b] # Peptides is the number of distinct peptide sequences in the protein. [c] # PSMs is number of identified peptide spectrum matches identified from all included searches, including those redundantly identified. [d] # Unique peptide is the total number of distinct peptide sequences unique to the protein group. [e] #AAs is length of the protein sequence. [f] Calc pI (isoelectric point) number is theoretically calculated isoelectric point for the protein that is, the pH at which a particular molecule carries no net electrical charge. [g] Score Sequest HT is a protein score calculated by summing the individual scores for each peptide. The higher this score, the higher the individual

score of the peptide, allowing for better identification. [h] # Peptides: Sequest HT is the number of distinct peptide sequences in the identified protein.

Supplementary Table 1-6: Detailed information on commonly secreted proteins by 3 synovial membrane (SM) mesenchymal stem cells (MSCs) under inflammatory condition.

Accession	Description	Coverage [%][a]	# Peptides[b]	# PSMs[c]	# Unique Peptides[d]	# AAs[e]	MW [kDa]	Calc. pI[f]	Score Sequest HT[g]	# Peptides: Sequest HT[h]
A0A024QYT5	Serpin peptidase inhibitor, clade E (Nexin, plasminogen activator inhibitor type 1), member 1, isoform CRA_b OS=Homo sapiens OX=9606 GN=SERPINE1 PE=3 SV=1	22	6	10	6	402	45	7.2	24.61	6
A0A024QZL1	Proteoglycan 1, secretory granule, isoform CRA_a OS=Homo sapiens OX=9606 GN=PRG1 PE=4 SV=1	8	1	1	1	158	17.6	4.86	2.06	1
A0A024R1U8	Insulin-like growth factor binding protein 4, isoform CRA_a OS=Homo sapiens OX=9606 GN=IGFBP4 PE=4 SV=1	25	4	10	4	258	27.9	7.15	27.64	4
A0A024R222	Phosphoserine aminotransferase OS=Homo sapiens OX=9606 GN=PSAT1 PE=3 SV=1	4	1	3	1	370	40.4	7.66	6.75	1
A0A024R462	Fibronectin 1, isoform CRA_n OS=Homo sapiens OX=9606 GN=FN1 PE=4 SV=1	33	44	146	44	2355	259	5.73	424.23	44
A0A024R5Z7	Annexin OS=Homo sapiens OX=9606 GN=ANXA2 PE=3 SV=1	23	7	16	7	339	38.6	7.75	41	7
A0A024R6R4	Matrix metallopeptidase 2 (Gelatinase A, 72kDa gelatinase, 72kDa type IV collagenase), isoform CRA_a OS=Homo sapiens OX=9606 GN=MMP2 PE=3 SV=1	23	10	21	10	660	73.8	5.47	63.07	10
A0A024R8V7	TIMP metallopeptidase inhibitor 2, isoform CRA_b OS=Homo sapiens OX=9606 GN=TIMP2 PE=4 SV=1	26	3	5	3	179	20.2	7.17	11.45	3
A0A024R969	Chitinase 3-like 1 (Cartilage glycoprotein-39), isoform CRA_a OS=Homo sapiens OX=9606 GN=CHI3L1 PE=3 SV=1	42	13	38	13	383	42.6	8.46	114.96	13
A0A024RDA5	Multifunctional fusion protein OS=Homo sapiens OX=9606 GN=IL8 PE=3 SV=1	16	1	8	1	99	11.1	8.84	22.59	1
A0A087WTA8	Collagen alpha-2(I) chain OS=Homo sapiens OX=9606 GN=COL1A2 PE=1 SV=1	32	25	67	25	1364	129.1	9.01	185.98	25
A0A087X0S5	Collagen alpha-1(VI) chain OS=Homo sapiens OX=9606 GN=COL6A1 PE=1 SV=1	14	9	22	9	1026	108.3	5.43	56.05	9
A0A0A0MT01	Gelsolin OS=Homo sapiens OX=9606 GN=GSN PE=1 SV=1	5	2	3	2	767	84.7	5.83	8.25	2
A0A0B4U5E3	Granulocyte-colony stimulating factor (Fragment) OS=Homo sapiens OX=9606 PE=2 SV=1	9	1	5	1	174	18.7	6.04	17.05	1
A0A0H3W617	Stanniocalcin 1 OS=Homo sapiens OX=9606 GN=stc1 PE=2 SV=1	25	3	8	3	178	20.5	8.72	21.14	3
A0A0S2Z3G9	Actinin alpha 4 isoform 1 (Fragment) OS=Homo sapiens OX=9606 GN=ACTN4 PE=2 SV=1	12	9	25	3	911	104.8	5.44	59.72	9
A0A0S2Z4G6	Tropomyosin 1 (Alpha), isoform CRA_o (Fragment) OS=Homo sapiens OX=9606 GN=TPM1 PE=1 SV=1	12	5	13	3	284	32.7	4.74	31.18	5
A0A161I202	Lactoferrin OS=Homo sapiens OX=9606 GN=LTF PE=2 SV=1	2	1	1	1	711	78.3	8.18	2.51	1
A0A172Q381	Endosialin (Fragment) OS=Homo sapiens OX=9606 GN=TEM1 PE=2 SV=1	2	1	1	1	668	71.2	5.01	2.16	1
A0A1B0GU92	Uncharacterized protein OS=Homo sapiens OX=9606 PE=1 SV=1	4	1	1	1	557	59.8	6.62	2.04	1
A0A1U9X7H4	CFB OS=Homo sapiens OX=9606 PE=3 SV=1	1	1	2	1	764	85.5	6.96	4.22	1
A4D1W7	Inhibin, beta A (Activin A, activin AB alpha polypeptide) OS=Homo sapiens OX=9606 GN=INHBA PE=2 SV=1	11	3	4	3	426	47.4	8.03	9.94	3
A4D2D2	Procollagen C-endopeptidase enhancer OS=Homo sapiens OX=9606 GN=PCOLCE PE=4 SV=1	10	3	5	3	449	47.9	7.43	12.96	3
A5GZ70	Matrix metalloproteinase 3 (Fragment) OS=Homo sapiens OX=9606 GN=MMP3 PE=2 SV=1	42	3	13	1	71	8.1	6.67	31.82	3
A5PLM9	Cathepsin L1 OS=Homo sapiens OX=9606 GN=CTSL1 PE=2 SV=1	4	1	3	1	333	37.5	5.45	8.55	1
A6XND1	Insulin-like growth factor binding protein 3 isoform b OS=Homo sapiens OX=9606 GN=IGFBP3 PE=1 SV=1	30	5	8	5	263	29	8.46	20.08	5
A8K7Q1	cDNA FLJ77770, highly similar to Homo sapiens nucleobindin 1 (NUCB1), mRNA OS=Homo sapiens OX=9606 PE=2 SV=1	3	1	1	1	461	53.9	5.25	1.92	1

Accession	Description	Coverage [%][a]	# Peptides[b]	# PSMs[c]	# Unique Peptides[d]	# AAs[e]	MW [kDa]	Calc. pI[f]	Score Sequest HT[g]	# Peptides: Sequest HT[h]
B2R4R0	Histone H4 OS=Homo sapiens OX=9606 GN=HIST1H4H PE=2 SV=1	41	4	13	4	103	11.4	11.36	35.67	4
B2R5H0	Protein S100 OS=Homo sapiens OX=9606 PE=2 SV=1	24	2	2	2	105	11.7	7.18	3.94	2
B2R5J8	C-C motif chemokine OS=Homo sapiens OX=9606 PE=2 SV=1	12	1	2	1	91	10	9.07	3.95	1
B2RBS8	cDNA, FLJ95666, highly similar to Homo sapiens albumin (ALB), mRNA OS=Homo sapiens OX=9606 PE=2 SV=1	2	2	9	2	609	69.3	6.28	24.5	2
B2RCM5	cDNA, FLJ96160, highly similar to Homo sapiens EGF-containing fibulin-like extracellular matrix protein 2 (EFEMP2), mRNA OS=Homo sapiens OX=9606 PE=2 SV=1	3	1	1	1	443	49.3	4.94	1.9	1
B2RDE1	cDNA, FLJ96568, highly similar to Homo sapiens tropomyosin 3 (TPM3), mRNA OS=Homo sapiens OX=9606 PE=2 SV=1	15	5	10	3	248	29	4.75	22.99	5
B2RDW0	cDNA, FLJ96792, highly similar to Homo sapiens calmodulin 2 (phosphorylase kinase, delta) (CALM2), mRNA OS=Homo sapiens OX=9606 PE=2 SV=1	21	2	3	2	149	16.8	4.22	7.48	2
B3KQT9	Protein disulfide-isomerase OS=Homo sapiens OX=9606 PE=2 SV=1	8	2	5	2	480	54.1	7.21	18.56	2
B4DDQ2	Biglycan OS=Homo sapiens OX=9606 PE=2 SV=1	18	5	16	5	334	37.6	7.97	40.41	5
B4DLV7	Rab GDP dissociation inhibitor OS=Homo sapiens OX=9606 PE=2 SV=1	10	3	4	3	449	51.1	8.18	9.47	3
B4DM82	Peptidyl-prolyl cis-trans isomerase OS=Homo sapiens OX=9606 PE=2 SV=1	34	3	8	3	129	14.1	8.47	21.75	3
B4DMR3	cDNA FLJ51896, highly similar to Glia-derived nexin OS=Homo sapiens OX=9606 PE=2 SV=1	40	12	46	12	334	37.1	9.52	121.2	12
B4DN59	cDNA FLJ52702, highly similar to Homo sapiens CD44 antigen (homing function and Indian blood group system) (CD44), transcript variant 4, mRNA OS=Homo sapiens OX=9606 PE=2 SV=1	3	1	1	1	329	36	5.9	2.16	1
B4DPQ0	Complement C1r subcomponent OS=Homo sapiens OX=9606 GN=C1R PE=1 SV=1	4	2	3	2	719	81.8	6.37	7.55	2
B4DPW5	cDNA FLJ59374, highly similar to Homo sapiens caldesmon 1 (CALD1), transcript variant 2, mRNA OS=Homo sapiens OX=9606 PE=2 SV=1	12	4	4	4	497	57.4	8.22	8.27	4
B4DPZ5	cDNA FLJ53495, highly similar to Polymerase I and transcript release factor OS=Homo sapiens OX=9606 PE=2 SV=1	8	2	4	2	363	40.5	5.25	10.39	2
B4E324	cDNA FLJ60397, highly similar to Lysosomal protective protein OS=Homo sapiens OX=9606 PE=2 SV=1	3	1	2	1	480	54.2	6.98	4.48	1
B4E3Q1	cDNA FLJ61580, highly similar to Calsyntenin-1 OS=Homo sapiens OX=9606 PE=2 SV=1	1	1	2	1	962	107.7	4.97	4.4	1
B4E3S0	Coronin OS=Homo sapiens OX=9606 GN=CORO1C PE=1 SV=1	4	1	1	1	369	41.6	6.73	2.25	1
B5MCZ3	Interleukin-6 OS=Homo sapiens OX=9606 GN=IL6 PE=1 SV=1	38	6	27	6	189	21.5	6.57	72.57	6
C9JIZ6	Prosaposin OS=Homo sapiens OX=9606 GN=PSAP PE=1 SV=2	6	2	4	2	527	58.4	5.17	10.47	2
D0PNI1	Epididymis luminal protein 4 OS=Homo sapiens OX=9606 GN=YWHAZ PE=2 SV=1	17	3	9	2	245	27.7	4.79	19	3
D3DTX7	Collagen, type I, alpha 1, isoform CRA_a OS=Homo sapiens OX=9606 GN=COL1A1 PE=4 SV=1	8	5	9	5	885	84.7	6.24	21.26	5
D3YTG3	Target of Nesh-SH3 OS=Homo sapiens OX=9606 GN=ABI3BP PE=1 SV=1	3	3	6	3	1777	195.2	9.73	15.57	3
D6RF92	C-X-C motif chemokine OS=Homo sapiens OX=9606 GN=CXCL6 PE=1 SV=1	28	3	15	2	113	11.9	9.76	34.57	3
D9ZGF2	Collagen, type VI, alpha 3 OS=Homo sapiens OX=9606 GN=COL6A3 PE=4 SV=1	6	15	21	15	3177	343.5	6.68	44.6	15
E5RGS7	Uncharacterized protein OS=Homo sapiens OX=9606 PE=4 SV=1	8	1	1	1	132	14.4	7.34	2.04	1
E5RI99	60S ribosomal protein L30 (Fragment) OS=Homo sapiens OX=9606 GN=RPL30 PE=1 SV=1	11	1	1	1	114	12.6	9.55	1.9	1

Accession	Description	Coverage [%][a]	# Peptides[b]	# PSMs[c]	# Unique Peptides[d]	# AAs[e]	MW [kDa]	Calc. pI[f]	Score Sequest HT[g]	# Peptides: Sequest HT[h]
E9PKE3	Heat shock cognate 71 kDa protein OS=Homo sapiens OX=9606 GN=HSPA8 PE=1 SV=1	14	5	13	4	627	68.8	5.52	32.21	5
F6RFD5	Destrin OS=Homo sapiens OX=9606 GN=DSTN PE=1 SV=1	18	2	3	2	135	15.4	8.59	6.73	2
F8W6I7	Heterogeneous nuclear ribonucleoprotein A1 OS=Homo sapiens OX=9606 GN=HNRNPA1 PE=1 SV=2	8	2	3	2	307	33.1	9.13	7.16	2
H0Y3T6	45 kDa calcium-binding protein (Fragment) OS=Homo sapiens OX=9606 GN=SDF4 PE=1 SV=1	5	1	1	1	243	28.1	5.1	2.03	1
I4AY87	Macrophage migration inhibitory factor (Fragment) OS=Homo sapiens OX=9606 GN=MIF PE=1 SV=1	10	1	1	1	115	12.5	7.88	3.24	1
M0QZH0	Reticulocalbin-3 (Fragment) OS=Homo sapiens OX=9606 GN=RCN3 PE=1 SV=1	17	2	2	2	169	19.8	4.98	4.04	2
O00300	Tumor necrosis factor receptor superfamily member 11B OS=Homo sapiens OX=9606 GN=TNFRSF11B PE=1 SV=3	7	2	5	2	401	46	8.29	11.84	2
O00391	Sulfhydryl oxidase 1 OS=Homo sapiens OX=9606 GN=QSOX1 PE=1 SV=3	11	6	7	6	747	82.5	8.92	20.01	6
O14992	HS24/P52 OS=Homo sapiens OX=9606 GN=HS24/p52 PE=2 SV=1	3	1	2	1	474	52.3	8.21	4.09	1
P01024	Complement C3 OS=Homo sapiens OX=9606 GN=C3 PE=1 SV=2	8	9	13	9	1663	187	6.4	32.25	9
P01033	Metalloproteinase inhibitor 1 OS=Homo sapiens OX=9606 GN=TIMP1 PE=1 SV=1	54	7	38	7	207	23.2	8.1	133.67	7
P01034	Cystatin-C OS=Homo sapiens OX=9606 GN=CST3 PE=1 SV=1	8	1	1	1	146	15.8	8.75	2.15	1
P02461	Collagen alpha-1(III) chain OS=Homo sapiens OX=9606 GN=COL3A1 PE=1 SV=4	3	3	8	3	1466	138.5	6.61	23.05	3
P02545	Prelamin-A/C OS=Homo sapiens OX=9606 GN=LMNA PE=1 SV=1	13	7	13	7	664	74.1	7.02	30.38	7
P04083	Annexin A1 OS=Homo sapiens OX=9606 GN=ANXA1 PE=1 SV=2	12	3	3	3	346	38.7	7.02	6.42	3
P04792	Heat shock protein beta-1 OS=Homo sapiens OX=9606 GN=HSPB1 PE=1 SV=2	15	1	2	1	205	22.8	6.4	5.49	1
P05387	60S acidic ribosomal protein P2 OS=Homo sapiens OX=9606 GN=RPLP2 PE=1 SV=1	14	1	1	1	115	11.7	4.54	2.41	1
P05388	60S acidic ribosomal protein P0 OS=Homo sapiens OX=9606 GN=RPLP0 PE=1 SV=1	4	1	1	1	317	34.3	5.97	1.99	1
P07585	Decorin OS=Homo sapiens OX=9606 GN=DCN PE=1 SV=1	27	7	17	7	359	39.7	8.54	44.76	7
P07737	Profilin-1 OS=Homo sapiens OX=9606 GN=PFN1 PE=1 SV=2	40	4	12	4	140	15	8.27	30.75	4
P07996	Thrombospondin-1 OS=Homo sapiens OX=9606 GN=THBS1 PE=1 SV=2	5	4	5	4	1170	129.3	4.94	10.86	4
P08254	Stromelysin-1 OS=Homo sapiens OX=9606 GN=MMP3 PE=1 SV=2	29	12	83	9	477	53.9	6.16	236.92	12
P08670	Vimentin OS=Homo sapiens OX=9606 GN=VIM PE=1 SV=4	45	19	75	19	466	53.6	5.12	202.46	19
P09238	Stromelysin-2 OS=Homo sapiens OX=9606 GN=MMP10 PE=1 SV=1	13	6	11	4	476	54.1	5.8	26.27	6
P09341	Growth-regulated alpha protein OS=Homo sapiens OX=9606 GN=CXCL1 PE=1 SV=1	38	4	50	3	107	11.3	10.43	145.53	4
P09382	Galectin-1 OS=Homo sapiens OX=9606 GN=LGALS1 PE=1 SV=2	35	3	15	3	135	14.7	5.5	43.06	3
P09486	SPARC OS=Homo sapiens OX=9606 GN=SPARC PE=1 SV=1	28	7	26	7	303	34.6	4.84	69.65	7
P09871	Complement C1s subcomponent OS=Homo sapiens OX=9606 GN=C1S PE=1 SV=1	6	3	6	3	688	76.6	4.96	14.52	3

Accession	Description	Coverage [%][a]	# Peptides[b]	# PSMs[c]	# Unique Peptides[d]	# AAs[e]	MW [kDa]	Calc. pI[f]	Score Sequest HT[g]	# Peptides: Sequest HT[h]
P10599	Thioredoxin OS=Homo sapiens OX=9606 GN=TXN PE=1 SV=3	31	3	8	3	105	11.7	4.92	20.45	3
P11047	Laminin subunit gamma-1 OS=Homo sapiens OX=9606 GN=LAMC1 PE=1 SV=3	2	2	5	2	1609	177.5	5.12	11.08	2
P12110	Collagen alpha-2(VI) chain OS=Homo sapiens OX=9606 GN=COL6A2 PE=1 SV=4	7	5	8	5	1019	108.5	6.21	21.22	5
P13500	C-C motif chemokine 2 OS=Homo sapiens OX=9606 GN=CCL2 PE=1 SV=1	11	1	5	1	99	11	9.25	12.44	1
P14543	Nidogen-1 OS=Homo sapiens OX=9606 GN=NID1 PE=1 SV=3	2	2	2	2	1247	136.3	5.29	4.64	2
P15018	Leukemia inhibitory factor OS=Homo sapiens OX=9606 GN=LIF PE=1 SV=1	10	2	2	2	202	22	9.35	4.56	2
P18206	Vinculin OS=Homo sapiens OX=9606 GN=VCL PE=1 SV=4	7	6	11	6	1134	123.7	5.66	24.42	6
P19875	C-X-C motif chemokine 2 OS=Homo sapiens OX=9606 GN=CXCL2 PE=1 SV=1	37	3	24	1	107	11.4	10.55	77.89	3
P19876	C-X-C motif chemokine 3 OS=Homo sapiens OX=9606 GN=CXCL3 PE=1 SV=1	38	3	25	1	107	11.3	10.37	81.71	3
P20809	Interleukin-11 OS=Homo sapiens OX=9606 GN=IL11 PE=1 SV=1	25	3	8	1	199	21.4	10.62	20.16	3
P21333	Filamin-A OS=Homo sapiens OX=9606 GN=FLNA PE=1 SV=4	9	13	18	13	2647	280.6	6.06	43.5	13
P24592	Insulin-like growth factor-binding protein 6 OS=Homo sapiens OX=9606 GN=IGFBP6 PE=1 SV=1	6	1	2	1	240	25.3	7.81	6.95	1
P26022	Pentraxin-related protein PTX3 OS=Homo sapiens OX=9606 GN=PTX3 PE=1 SV=3	20	6	17	6	381	41.9	5.01	46.62	6
P26038	Moesin OS=Homo sapiens OX=9606 GN=MSN PE=1 SV=3	12	5	8	5	577	67.8	6.4	17.85	5
P26447	Protein S100-A4 OS=Homo sapiens OX=9606 GN=S100A4 PE=1 SV=1	11	1	1	1	101	11.7	6.11	2.04	1
P30044	Peroxiredoxin-5, mitochondrial OS=Homo sapiens OX=9606 GN=PRDX5 PE=1 SV=4	5	1	1	1	214	22.1	8.7	2.21	1
P35442	Thrombospondin-2 OS=Homo sapiens OX=9606 GN=THBS2 PE=1 SV=2	3	2	2	2	1172	129.9	4.83	4.49	2
P35555	Fibrillin-1 OS=Homo sapiens OX=9606 GN=FBN1 PE=1 SV=3	6	11	18	11	2871	312	4.93	47.21	11
P48307	Tissue factor pathway inhibitor 2 OS=Homo sapiens OX=9606 GN=TFPI2 PE=1 SV=1	17	3	4	3	235	26.9	8.53	9.47	3
P50454	Serpin H1 OS=Homo sapiens OX=9606 GN=SERPINH1 PE=1 SV=2	14	5	10	5	418	46.4	8.69	24.55	5
P51884	Lumican OS=Homo sapiens OX=9606 GN=LUM PE=1 SV=2	20	4	15	4	338	38.4	6.61	46.15	4
P51991	Heterogeneous nuclear ribonucleoprotein A3 OS=Homo sapiens OX=9606 GN=HNRNPA3 PE=1 SV=2	6	2	3	2	378	39.6	9.01	6.34	2
P62328	Thymosin beta-4 OS=Homo sapiens OX=9606 GN=TMSB4X PE=1 SV=2	34	2	4	2	44	5.1	5.06	9.8	2
P63267	Actin, gamma-enteric smooth muscle OS=Homo sapiens OX=9606 GN=ACTG2 PE=1 SV=1	14	4	13	1	376	41.9	5.48	34.07	4
P63313	Thymosin beta-10 OS=Homo sapiens OX=9606 GN=TMSB10 PE=1 SV=2	34	1	4	1	44	5	5.36	10.17	1
P84243	Histone H3.3 OS=Homo sapiens OX=9606 GN=H3F3A PE=1 SV=2	5	1	2	1	136	15.3	11.27	3.9	1
P98066	Tumor necrosis factor-inducible gene 6 protein OS=Homo sapiens OX=9606 GN=TNFAIP6 PE=1 SV=2	9	2	2	2	277	31.2	6.79	4.57	2
P98160	Basement membrane-specific heparan sulfate proteoglycan core protein OS=Homo sapiens OX=9606 GN=HSPG2 PE=1 SV=4	0	1	1	1	4391	468.5	6.51	2.16	1

Accession	Description	Coverage [%][a]	# Peptides[b]	# PSMs[c]	# Unique Peptides[d]	# AAs[e]	MW [kDa]	Calc. pI[f]	Score Sequest HT[g]	# Peptides: Sequest HT[h]
Q12841	Follistatin-related protein 1 OS=Homo sapiens OX=9606 GN=FSTL1 PE=1 SV=1	26	6	17	6	308	35	5.52	50.44	6
Q16270	Insulin-like growth factor-binding protein 7 OS=Homo sapiens OX=9606 GN=IGFBP7 PE=1 SV=1	33	6	17	6	282	29.1	7.9	48.49	6
Q16777	Histone H2A type 2-C OS=Homo sapiens OX=9606 GN=HIST2H2AC PE=1 SV=4	22	2	7	2	129	14	10.9	21.95	2
Q16778	Histone H2B type 2-E OS=Homo sapiens OX=9606 GN=HIST2H2BE PE=1 SV=3	12	1	3	1	126	13.9	10.32	9.88	1
Q53FR6	Cartilage oligomeric matrix protein variant (Fragment) OS=Homo sapiens OX=9606 PE=2 SV=1	9	4	8	4	757	82.7	4.61	20.47	4
Q53G71	Calreticulin variant (Fragment) OS=Homo sapiens OX=9606 PE=2 SV=1	6	1	2	1	406	46.9	4.45	6.38	1
Q53G75	Matrix metalloproteinase 1 preproprotein variant (Fragment) OS=Homo sapiens OX=9606 PE=2 SV=1	29	10	30	10	405	46.5	7.03	75.1	10
Q53G99	Beta actin variant (Fragment) OS=Homo sapiens OX=9606 PE=2 SV=1	42	10	35	1	375	41.7	5.59	100.89	10
Q53GY0	Plastin 3 variant (Fragment) OS=Homo sapiens OX=9606 PE=2 SV=1	1	1	1	1	630	70.7	5.68	2.16	1
Q53HQ7	Elongation factor 1-alpha (Fragment) OS=Homo sapiens OX=9606 PE=2 SV=1	21	6	13	6	462	50.2	8.94	37.39	6
Q5UGI6	Serine/cysteine proteinase inhibitor clade G member 1 splice variant 2 (Fragment) OS=Homo sapiens OX=9606 GN=SERPING1 PE=2 SV=1	11	3	5	3	333	37.3	8	12.15	3
Q6EMK4	Vasorin OS=Homo sapiens OX=9606 GN=VASN PE=1 SV=1	4	2	2	2	673	71.7	7.39	5.16	2
Q6FHZ0	Malate dehydrogenase OS=Homo sapiens OX=9606 GN=MDH2 PE=2 SV=1	4	1	2	1	338	35.5	8.68	5.17	1
Q6IAW5	CALU protein OS=Homo sapiens OX=9606 GN=CALU PE=2 SV=1	25	5	8	5	315	37.1	4.64	21.1	5
Q75MU2	Uncharacterized protein WBSCR1 (Fragment) OS=Homo sapiens OX=9606 GN=WBSCR1 PE=4 SV=1	8	1	2	1	156	17.1	5.31	4.53	1
Q86Z22	Epididymis secretory protein Li 297 OS=Homo sapiens OX=9606 GN=HEL-S-297 PE=2 SV=1	7	1	5	1	226	23.8	9.06	14.6	1
Q8IUX7	Adipocyte enhancer-binding protein 1 OS=Homo sapiens OX=9606 GN=AEBP1 PE=1 SV=1	1	1	1	1	1158	130.8	5.11	2.08	1
Q96AY3	Peptidyl-prolyl cis-trans isomerase FKBP10 OS=Homo sapiens OX=9606 GN=FKBP10 PE=1 SV=1	4	2	2	2	582	64.2	5.62	4.23	2
Q99715	Collagen alpha-1(XII) chain OS=Homo sapiens OX=9606 GN=COL12A1 PE=1 SV=2	1	2	5	2	3063	332.9	5.53	11.09	2
Q9H4F8	SPARC-related modular calcium-binding protein 1 OS=Homo sapiens OX=9606 GN=SMOC1 PE=1 SV=1	3	1	3	1	434	48.1	8.22	7.66	1
S4R3N1	HSPE1-MOB4 readthrough OS=Homo sapiens OX=9606 GN=HSPE1-MOB4 PE=3 SV=1	5	1	2	1	261	29.7	6.16	4.33	1
V9HWB4	Epididymis secretory sperm binding protein Li 89n OS=Homo sapiens OX=9606 GN=HEL-S-89n PE=2 SV=1	16	7	15	6	654	72.3	5.16	41.96	7
V9HWC6	Peptidyl-prolyl cis-trans isomerase OS=Homo sapiens OX=9606 GN=HEL-S-39 PE=2 SV=1	23	4	4	4	208	22.7	9.32	9.47	4
V9HWI5	Cofilin 1 (Non-muscle), isoform CRA_b OS=Homo sapiens OX=9606 GN=HEL-S-15 PE=2 SV=1	33	3	7	3	166	18.5	8.09	18.78	3
V9HWK1	Triosephosphate isomerase OS=Homo sapiens OX=9606 GN=HEL-S-49 PE=2 SV=1	45	6	12	6	249	26.7	6.9	32.09	6
V9HWN7	Fructose-bisphosphate aldolase OS=Homo sapiens OX=9606 GN=HEL-S-87p PE=2 SV=1	20	5	11	5	364	39.4	8.09	31.33	5
V9HWP2	Epididymis luminal protein 35 OS=Homo sapiens OX=9606 GN=HEL-S-125m PE=2 SV=1	6	4	6	4	803	92.4	4.84	13.86	4

[a] Coverage is the percentage of the sequence covered by identifications (PSMs only) from the included searches. [b] # Peptides is the number of distinct peptide sequences in the protein. [c] # PSMs is number of identified peptide spectrum matches identified from

all included searches, including those redundantly identified. [d] # Unique peptide is the total number of distinct peptide sequences unique to the protein group. [e] #AAs is length of the protein sequence. [f] Calc pI (isoelectric point) number is theoretically calculated isoelectric point for the protein that is, the pH at which a particular molecule carries no net electrical charge. [g] Score Sequest HT is a protein score calculated by summing the individual scores for each peptide. The higher this score, the higher the individual score of the peptide, allowing for better identification. [h] # Peptides: Sequest HT is the number of distinct peptide sequences in the identified protein.
